# Supplementary material for: Association of obstructive sleep apnoea with the risk of vascular outcomes and all-cause mortality: a meta-analysis
Source: BMJ Open. 2017 Dec 22;7(12):e013983. doi: 10.1136/bmjopen-2016-013983 (PMC5770910; doi:10.1136/bmjopen-2016-013983)
Supplement: Supplementary file 1 [file bmjopen-2016-013983supp001.pdf]

Title:

	**Search strategy**
**#1**	“Sleep Apnea, Obstructive” [Mesh] OR “OSA” [All fields] OR “OHS” [All fields]
**#2**	Apneas, Obstructive Sleep OR Obstructive Sleep Apneas OR Sleep Apneas, Obstructive OR Obstructive Sleep Apnea Syndrome OR Obstructive Sleep Apnea OR OSAHS OR Syndrome, Sleep Apnea, Obstructive OR Sleep Apnea Syndrome, Obstructive OR Apnea, Obstructive Sleep OR Sleep Apnea Hypopnea Syndrome OR Syndrome, Obstructive Sleep Apnea OR Upper Airway Resistance Sleep Apnea Syndrome OR Syndrome, Upper Airway Resistance, Sleep Apnea OR Hypoventilation Syndrome, Obesity OR Syndrome, Obesity Hypoventilation OR Pickwickian Syndrome OR Syndrome, Pickwickian OR Obesity-Hypoventilation Syndrome
**#3**	“Sleep Apnea Syndromes” [Mesh] OR “SAS” [All fields]
**#4**	Apnea Syndrome, Sleep OR Apnea Syndromes, Sleep OR Sleep Apnea Syndrome OR Apnea, Sleep OR Apneas, Sleep OR Sleep Apnea OR Sleep Apneas OR Sleep Hypopnea OR Hypopnea, Sleep OR Hypopneas, Sleep OR Sleep Hypopneas OR Sleep-Disordered Breathing OR Breathing, Sleep-Disordered OR Sleep Disordered Breathing OR Sleep Apnea, Mixed Central and Obstructive OR Mixed Central and Obstructive Sleep Apnea OR Sleep Apnea, Mixed OR Mixed Sleep Apnea OR Mixed Sleep Apneas OR Sleep Apneas, Mixed OR Hypersomnia with Periodic Respiration
**#5**	“Sleep Apnea, Central” [Mesh] OR “CSA”[All fields]
**#6**	Apneas, Central Sleep OR Central Sleep Apneas OR Sleep Apneas, Central OR Apnea, Central OR Apneas, Central OR Central Apnea OR Central Apneas OR Apnea, Central Sleep OR Apnea, Sleep, Central OR Sleep Apnea, Lethal Central OR Central Sleep Apnea OR Central Sleep Apnea Syndrome OR Central Sleep Disordered Breathing OR Hypoventilation, Central Alveolar OR Alveolar Hypoventilation, Central OR Alveolar Hypoventilations, Central OR Central Alveolar Hypoventilation OR Hypoventilations, Central Alveolar OR Ondine Syndrome OR Sleep-Disordered Breathing, Central OR Breathing, Central Sleep-Disordered OR Breathings, Central Sleep-Disordered OR Central Sleep-Disordered Breathing OR Central Sleep-Disordered Breathings OR Sleep Disordered Breathing, Central OR Sleep-Disordered Breathings, Central OR Central Alveolar Hypoventilation Syndrome OR Central Sleep Apnea, Secondary OR Secondary Central Sleep Apnea OR Sleep Apnea, Newborn, Primary OR Primary Sleep Apneas of Newborn OR Newborn Primary Sleep Apneas OR Central Sleep Apnea, Primary OR Primary Central Sleep Apnea
**#7**	“Continuous Positive Airway Pressure” [Mesh] OR “CPAP” [All fields] OR “Continuous Positive Airway Pressure/therapy” [Mesh]
**#8**	CPAP Ventilation OR Ventilation, CPAP OR Biphasic Continuous Positive Airway Pressure OR Bilevel Continuous Positive Airway Pressure OR Nasal Continuous Positive Airway Pressure OR nCPAP Ventilation OR Ventilation, nCPAP OR Airway Pressure Release Ventilation OR APRV Ventilation Mode OR APRV Ventilation Modes OR Ventilation Mode, APRV OR Ventilation Modes, APRV

**#9**	#1 OR #2 OR #3 OR #4 OR #5 OR #6 OR #7 OR #8
#10	“Cardiovascular System” [Mesh]
#11	“Death” [Mesh] OR Determination of Death OR Near-Death Experience OR Cardiac Death OR Death, Cardiac OR “Mortality” [Mesh] OR “mortality” [All fields] OR “Disease” [Mesh] OR “disease*”
**#12**	#10 AND #11
**#13**	“Cardiovascular Diseases” [Mesh] OR “CVD” OR Cardiovascular Disease OR Disease, Cardiovascular OR Diseases, Cardiovascular
**#14**	“Myocardial Infarction” [Mesh] OR “MI” OR Infarction, Myocardial OR Infarctions, Myocardial OR Myocardial Infarctions OR Cardiovascular Stroke OR Cardiovascular Strokes OR Stroke, Cardiovascular OR Strokes, Cardiovascular OR Heart Attack OR Heart Attacks OR Myocardial Infarct OR Infarct, Myocardial OR Infarcts, Myocardial OR Myocardial Infarcts
**#15**	“Angina Pectoris” [Mesh] OR “Angina, Stable” [Mesh] OR “Microvascular Angina” [Mesh] OR “Angina, Unstable” [Mesh] OR Stenocardia OR Stenocardias OR Angor Pectoris OR “angina” [All fields] OR “Coronary Artery Disease” [Mesh] OR “CAD” OR “ischemic heart disease” [All fields] OR “Heart Failure” [Mesh] OR “Heart Failure, Diastolic” [Mesh] OR “Heart Failure, Systolic” [Mesh]
**#16**	“Cerebrovascular Disorders” [Mesh] OR “cerebrovascular” [All fields] OR “stroke*”
**#17**	“Death” [Mesh] OR Determination of Death OR Near-Death Experience OR Cardiac Death OR Death, Cardiac OR “Mortality” [Mesh] OR “mortality” [All fields] OR “mortality*”
**#18**	#12 OR #13 OR #14 OR #15 OR #16 OR #17
**#19**	“Prospective Studies” [Mesh] OR “Cohort Studies” [Mesh] OR “Follow-Up Studies” [Mesh] OR “prospective study” OR “cohort study” OR “follow-up study”
**#20**	#9 AND #18 AND #19
