# Supplementary material for: Association of obstructive sleep apnoea with the risk of vascular outcomes and all-cause mortality: a meta-analysis
Source: BMJ Open. 2017 Dec 22;7(12):e013983. doi: 10.1136/bmjopen-2016-013983 (PMC5770910; doi:10.1136/bmjopen-2016-013983)
Supplement: Supplementary file 2 [file bmjopen-2016-013983supp002.pdf]

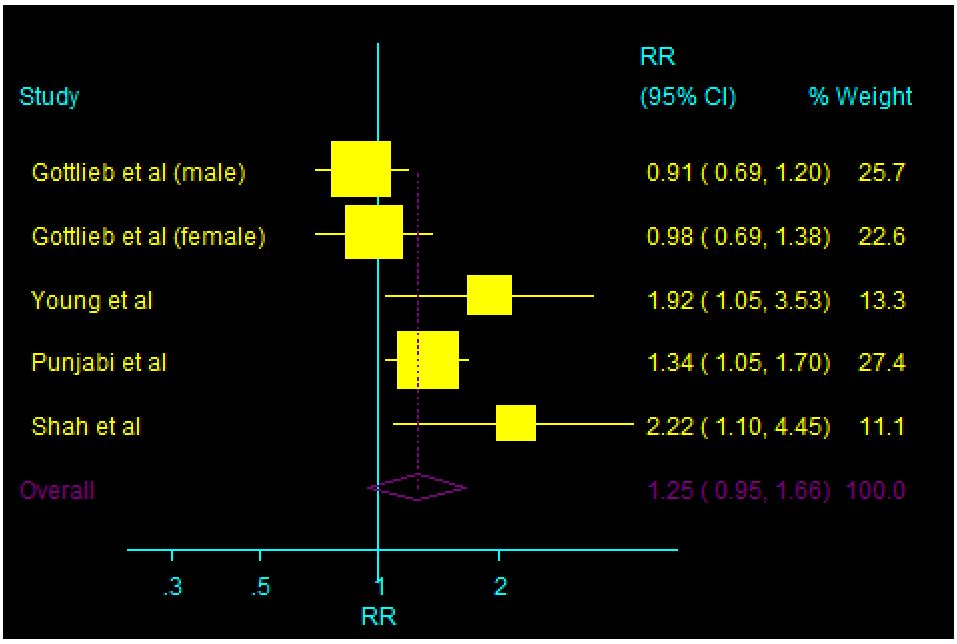

Figure S1. Association between mild OSA and CHD.

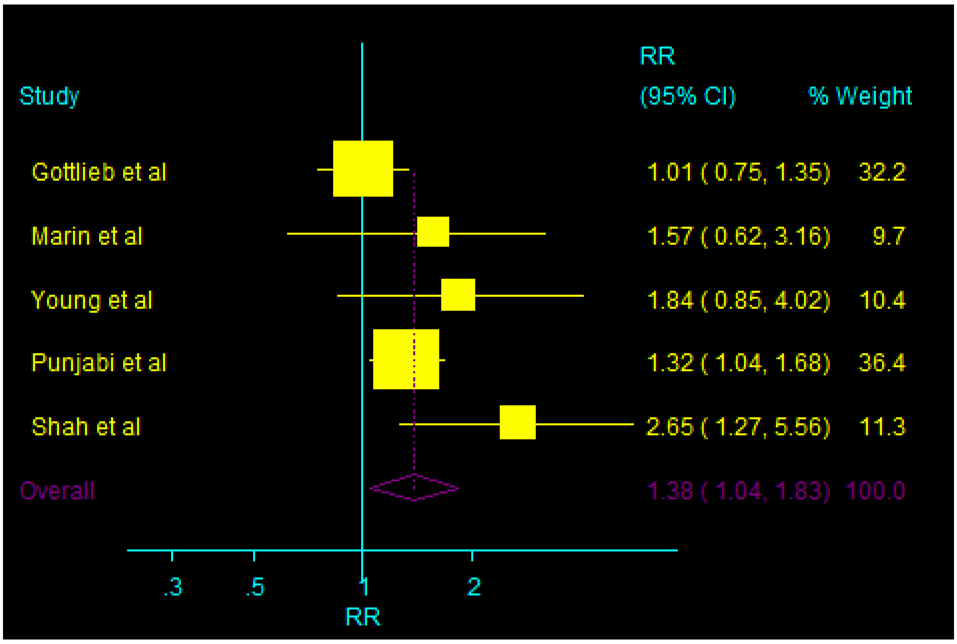

Figure S2. Association between moderate OSA and CHD.

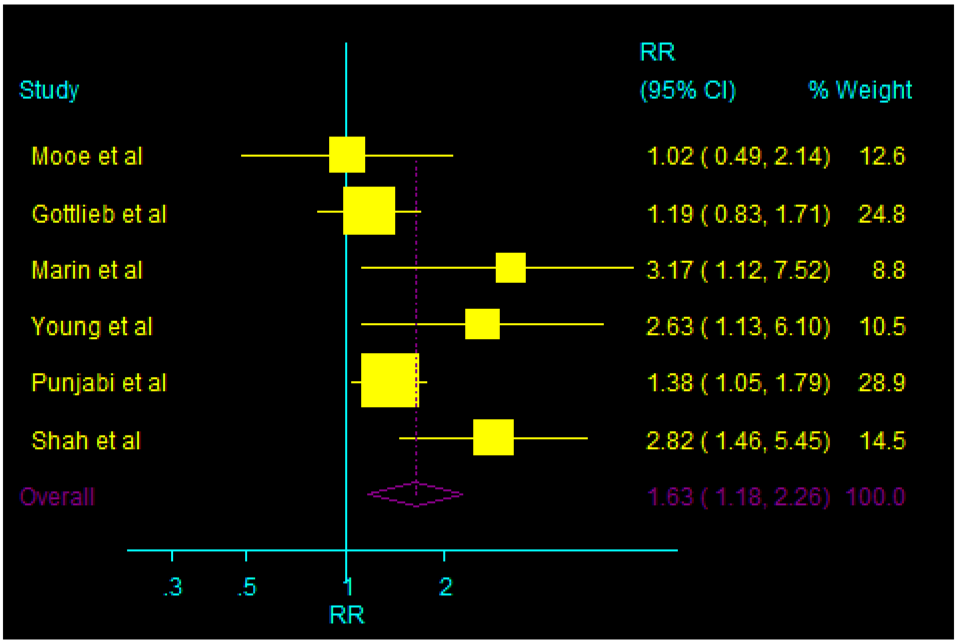

Figure S3. Association between severe OSA and CHD.

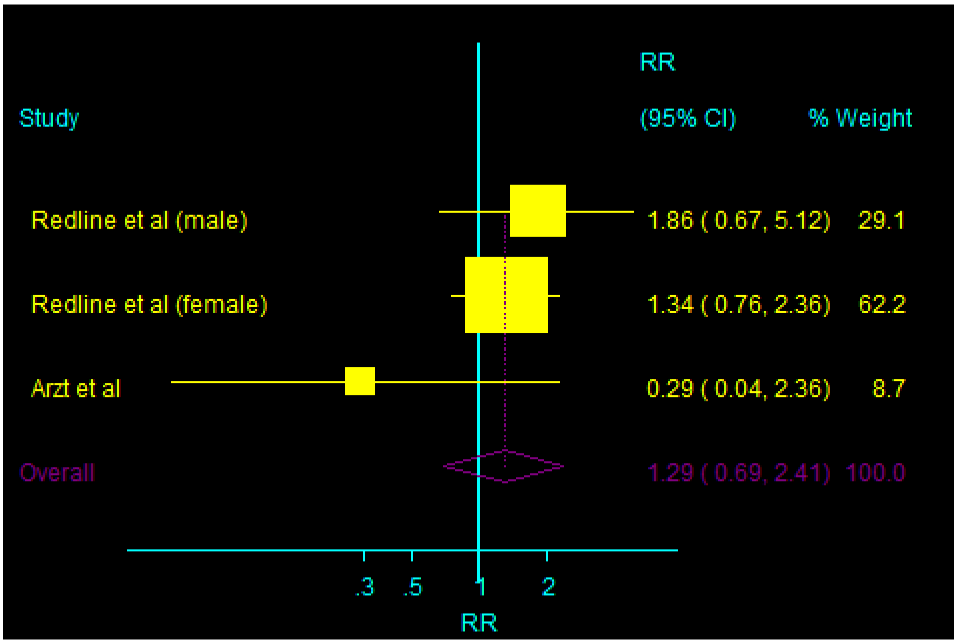

Figure S4. Association between mild OSA and stroke.

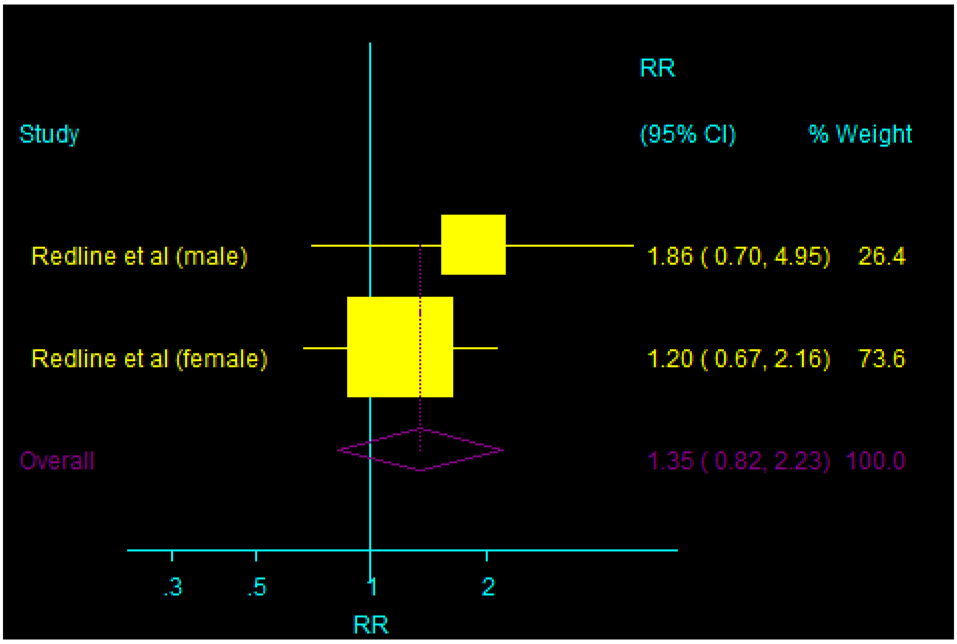

Figure S5. Association between moderate OSA and stroke.

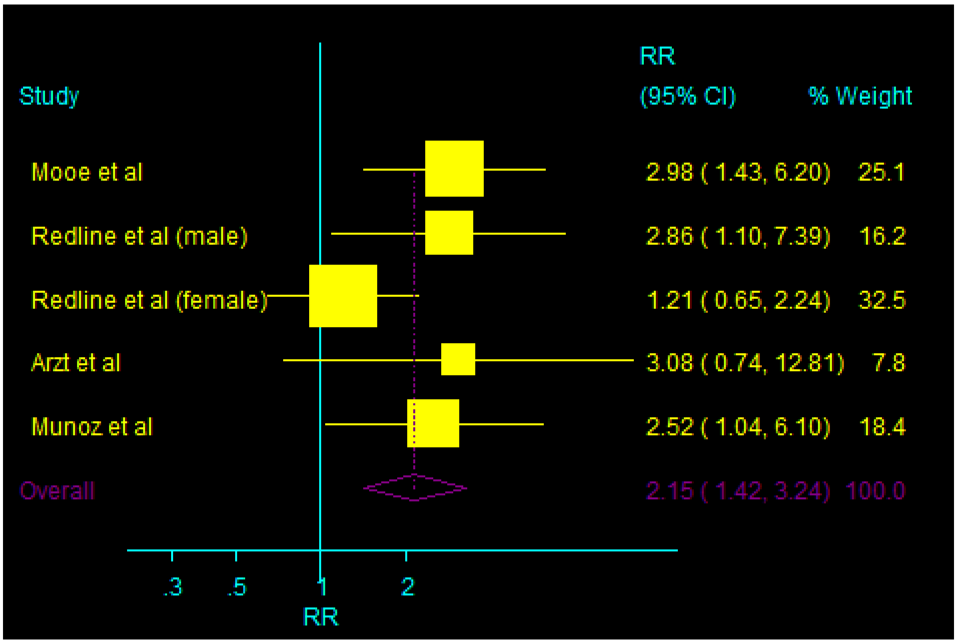

Figure S6. Association between severe OSA and stroke

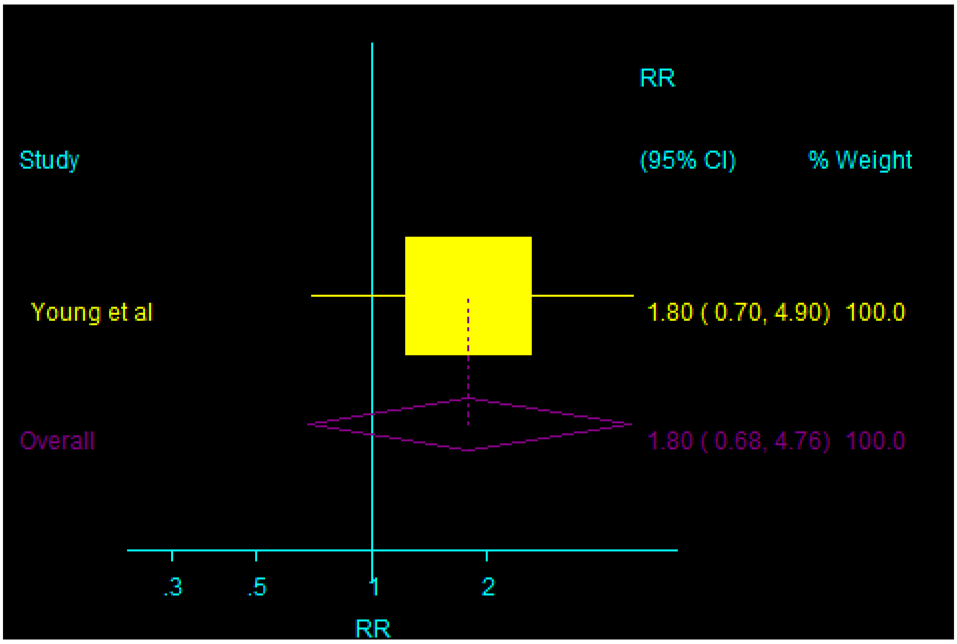

Figure S7. Association between mild OSA and cardiac death.

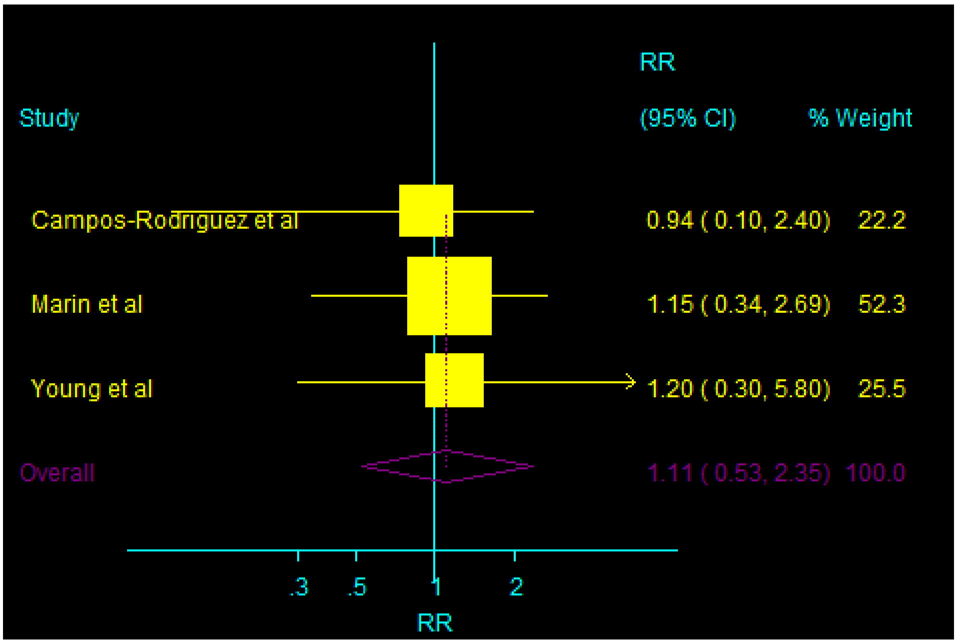

Figure S8. Association between moderate OSA and cardiac death.

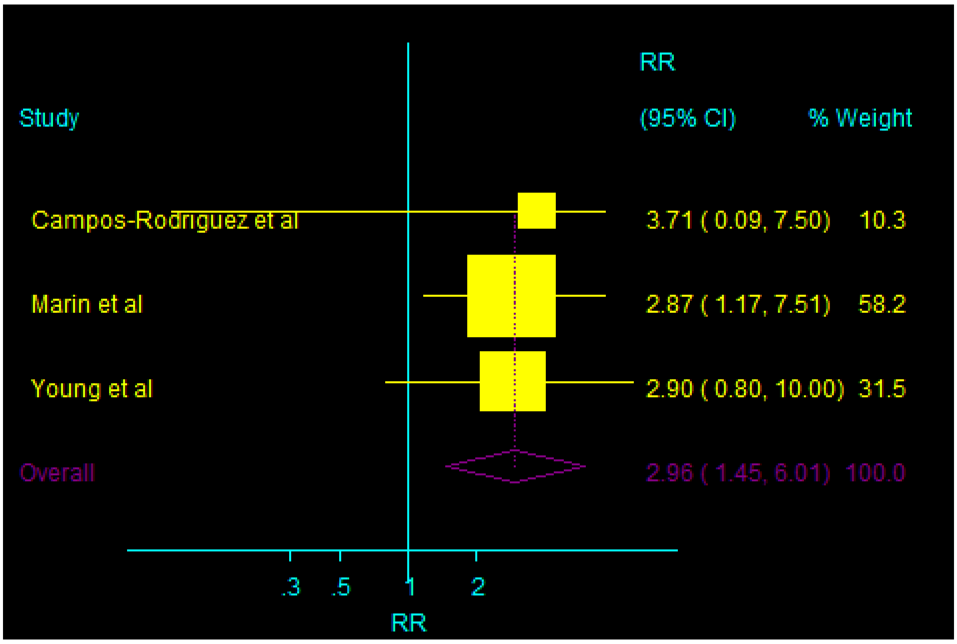

Figure S9. Association between severe OSA and cardiac death.

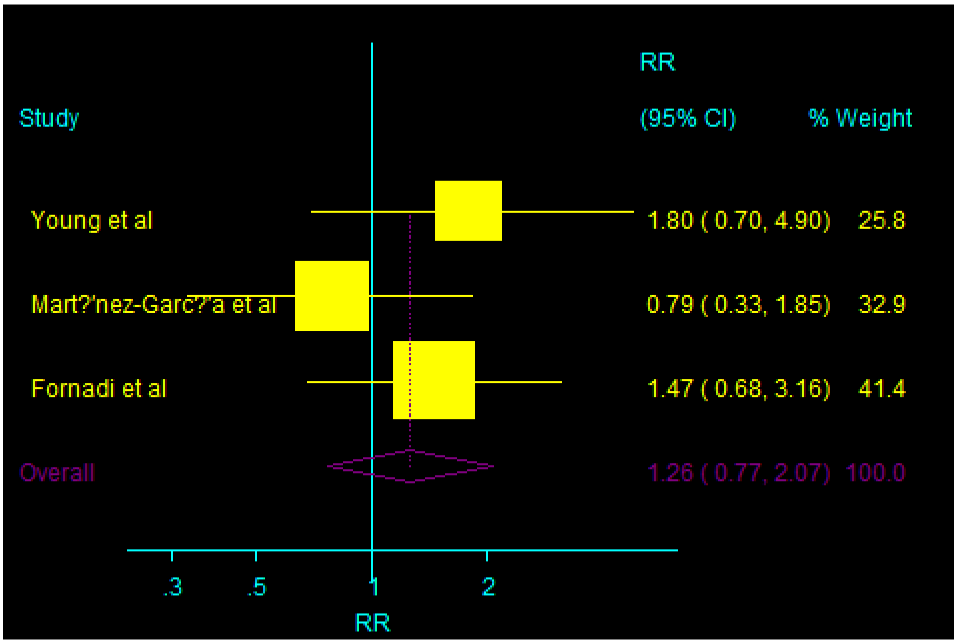

Figure S10. Association between mild OSA and all-cause death.

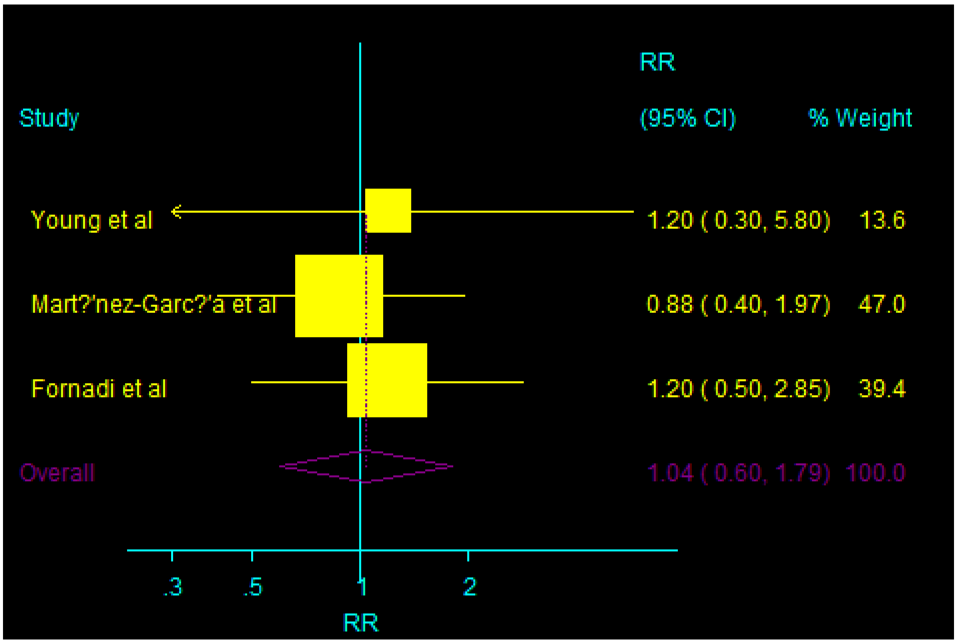

Figure S11. Association between moderate OSA and all-cause death.

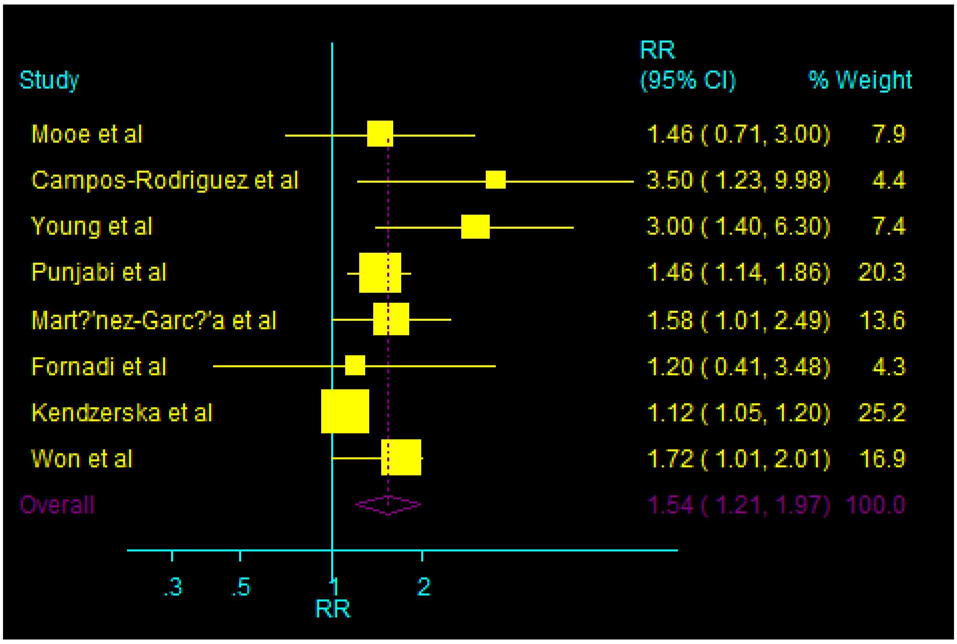

Figure S12. Association between severe OSA and all-cause death.

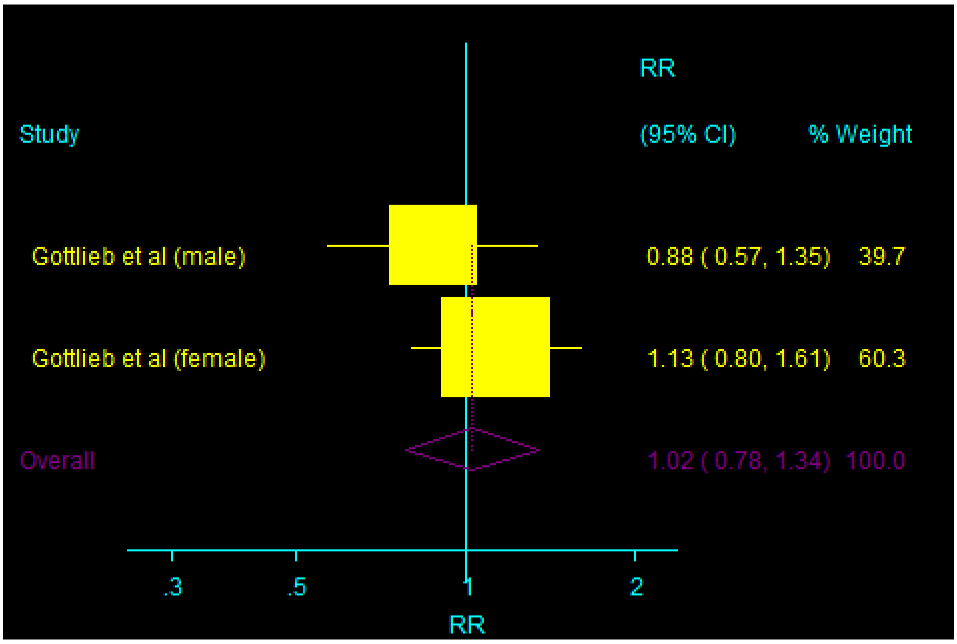

Figure S13. Association between mild OSA and heart failure.

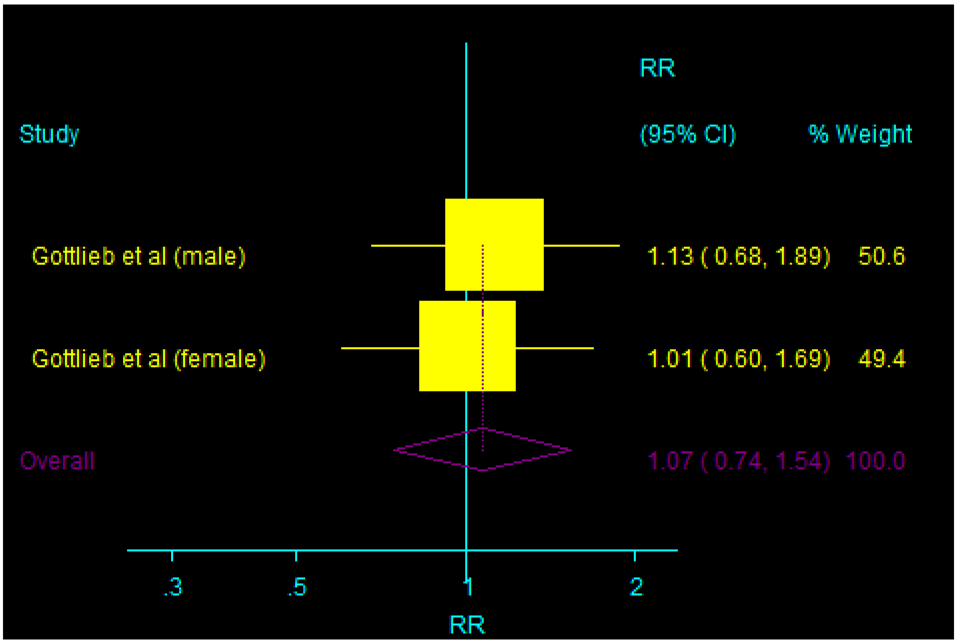

Figure S14. Association between moderate OSA and heart failure.

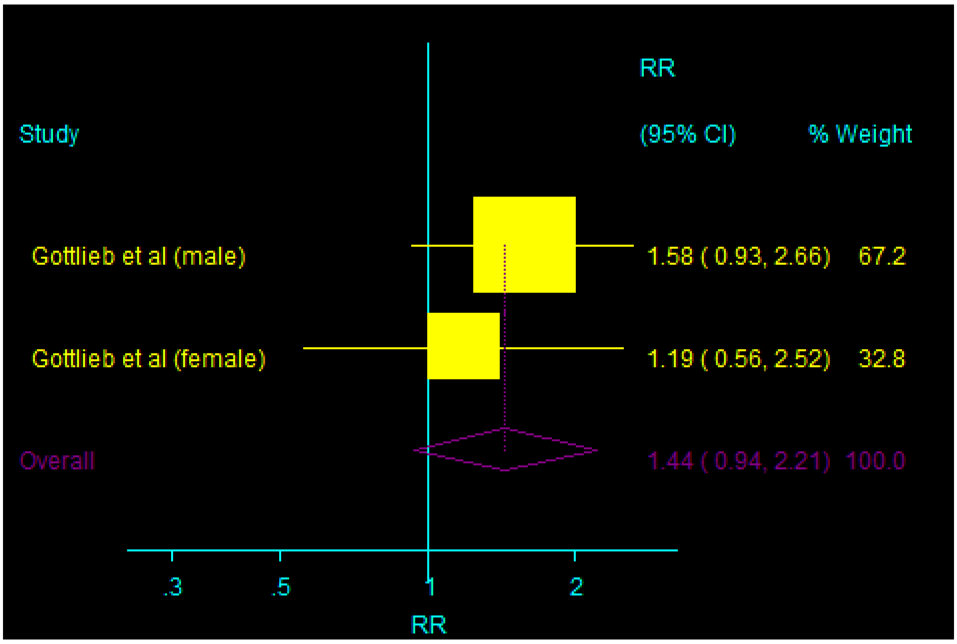

Figure S15. Association between severe OSA and heart failure.
