# Supplementary material for: Association of obstructive sleep apnoea with the risk of vascular outcomes and all-cause mortality: a meta-analysis
Source: BMJ Open. 2017 Dec 22;7(12):e013983. doi: 10.1136/bmjopen-2016-013983 (PMC5770910; doi:10.1136/bmjopen-2016-013983)
Supplement: Author's manuscript [file bmjopen-2016-013983.draft_revisions.pdf]

BMJ Open is committed to open peer review. As part of this commitment we make the peer review history of every article we publish publicly available.

When an article is published we post the peer reviewers' comments and the authors' responses online. We also post the versions of the paper that were used during peer review. These are the versions that the peer review comments apply to.

The versions of the paper that follow are the versions that were submitted during the peer review process. They are not the versions of record or the final published versions. They should not be cited or distributed as the published version of this manuscript.

BMJ Open is an open access journal and the full, final, typeset and author-corrected version of record of the manuscript is available on our site with no access controls, subscription charges or pay-per-view fees (http://bmjopen.bmj.com).

If you have any questions on BMJ Open's open peer review process please email editorial.bmjopen@bmj.com

Association of obstructive sleep apnea with the risk of vascular outcomes and total mortality: a meta-analysis

Journal:	*BMJ Open*
Manuscript ID	bmjopen-2016-013983
Article Type:	Research
Date Submitted by the Author:	30-Aug-2016
Complete List of Authors:	Xie, Chengjuan; The First Affiliated Hospital of Anhui Medical University, Department of Neurology<br>Zhu, Ruolin; The First Affiliated Hospital of Anhui Medical University, Department of Neurology<br>Tian, Yanghua; The First Affiliated Hospital of Anhui Medical University, Department of Neurology<br>Wang, Kai; Department of Neurology
**Primary Subject Heading**:	Neurology
Secondary Subject Heading:	Cardiovascular medicine
Keywords:	meta-analysis, mortality, obstructive sleep apnea, vascular outcome

SCHOLARONE™
Manuscripts

Association of obstructive sleep apnea with the risk of vascular outcomes and total mortality: a meta-analysis

Chengjuan Xie[1], Ruolin Zhu[1], Yanghua Tian[1,2], Kai Wang[1, 2, 3,*]

[1]Department of Neurology, The First Affiliated Hospital of Anhui Medical University, Hefei, China

[2]Collaborative Innovation Centre of Neuropsychiatric Disorders and Mental Health, Anhui Province, China

[3]Department of Medical Psychology, Anhui Medical University, Hefei, China

***Correspondence author:**

Kai Wang,

Department of Neurology, The First Affiliated Hospital of Anhui Medical University, Hefei, 230022,China;Collaborative Innovation Centre of Neuropsychiatric Disorders and Mental Health, Anhui Province, 230022,China;Department of Medical Psychology, Anhui Medical University, Hefei, 230022,China

Tel: +86-0551-62922418,

Fax:+86-021-64085875,

E-mail: xiecj2016@163.com.

Word count:5459

For peer review only

Abstract

Objective: This study aimed to conduct a meta-analysis to explore and summarize the evidence regarding the association between obstructive sleep apnea (OSA) and the subsequent risk for vascular outcomes and total mortality.

Methods: Electronic databases PubMed, EmBase, and the Cochrane Library were searched to identify studies conducted through May 2016. Prospective cohort studies that reported effect estimates with 95% confidence intervals of major adverse cardiac events (MACEs), coronary heart disease (CHD), stroke, cardiac death, total mortality, and heart failure for different levels versus the lowest level of OSA were included.

Results: A total of 16 cohort studies reporting data on 36,363 individuals were included. Severe OSA was associated with a greater risk of MACEs ($P < 0.001$), CHD ($P = 0.003$), stroke ($P < 0.001$), cardiac death ($P = 0.003$), and total mortality ($P < 0.001$); moderate OSA had a harmful impact on MACEs ($P = 0.034$) and CHD ($P = 0.026$); and no significant association was found between mild OSA and the risk of vascular outcomes or total mortality ($P > 0.05$). Finally, no evidence of a factor-specific difference in the risk ratio for MACEs among participants with different levels of OSA compared with those with the lowest level of OSA was found.

Conclusions: This study indicated that severe and moderate OSA were associated with an increased risk of vascular outcomes and total mortality. This relationship might differ between genders. Therefore, further large-scale prospective studies are needed to verify this difference.

For peer review only

Key words: meta-analysis; mortality, obstructive sleep apnea, vascular outcome

Article Summary:

Strengths and limitations of this study

1. Degree of association of OSA to fatal and non-fatal CDs is gender specific.

2. Statistical evidence on association of moderate-to-severe OSA with MACEs.

3. Peculiar study design assesses risk-ratios as per patient characteristics.

4. Quantitative data to emphasize association of OSA as a poignant factor for CDs.

Introduction

Obstructive sleep apnea (OSA) affects 24% of middle-aged men and 9% of women in the USA, but daytime sleepiness was reported in 17% and 22% of these subjects, respectively[1]. OSA is an increasingly prevalent condition characterized by repetitive obstruction of the upper airway during sleep accompanied by episodic hypoxia, arousal, and sleep fragmentation[2]. Previous studies suggest that OSA was associated with increased risk of glaucoma, diabetic kidney disease, and metabolic syndrome[3-5]. However, data on the association between OSA and the risk of subsequent vascular outcomes and mortality are both limited and inconclusive. Furthermore, whether these relationships differ according to the characteristics of patients with OSA also needs to be verified.

Several meta-analyses have illustrated that continuous positive airway pressure (CPAP) interventions aimed at OSA may reduce the risk of cardiovascular outcomes.

For peer review only

Kim et al[6] showed that CPAP treatment for OSA was associated with a lower incidence of stroke and cardiac events. Furthermore, Bratton et al[7] indicated that among patients with OSA, use of both CPAP and mandibular advancement devices was associated with reductions in blood pressure. Nadeem et al[8] suggested that CPAP treatment for OSA seemed to improve dyslipidemia (decrease in total cholesterol and low-density lipoprotein, and increase in high-density lipoprotein), whereas it does not appear to affect the triglyceride levels. These studies recommend that patients with OSA who receive interventions have a reduced risk of cardiovascular diseases. Therefore, clarifying the relationship between OSA and vascular outcomes is particularly important as it has not been definitively determined. This study attempted to perform a large-scale examination of the available prospective studies to determine the association of OSA with the potential risk of vascular outcomes and total mortality.

Methods

Data sources, search strategy, and selection criteria

This study was conducted and reported according to the Meta-analysis of Observational Studies in Epidemiology protocol[9].

Any prospective cohort study that examined the relationship between OSA and vascular outcomes or total mortality was eligible for inclusion in this study, and no restrictions were placed on language or publication status (e.g., published, in press, or in progress). Electronic databases PubMed, EmBase, and the Cochrane Library were

For peer review only

searched for articles published through May 2016, using the terms “sleep apnea” OR “obstructive sleep apneas” AND (“cardiovascular disease” OR “stroke” OR “cardiac death”OR “mortality” OR “death” OR “CVD” OR “myocardial infarction” OR “coronary events”) AND “clinical trials” AND “human” as the search terms (Supplemental 1). Manual searches of reference lists were also conducted from all the relevant original and reviewed articles to identify additional eligible studies. The medical subject heading, methods, patient population, design, exposure, and outcome variables of these articles were used to identify the relevant studies.

The literature search was independently undertaken by two authors using a standardized approach. Any inconsistencies between these two authors were settled by the primary author until a consensus was reached. The study was eligible for inclusion if the following criteria were met: (1) the study had a prospective cohort design; (2) the study investigated the association between OSA and the risk of major adverse cardiac events (MACEs), coronary heart disease (CHD), stroke, cardiac death, total mortality, and heart failure; and (3) the authors reported effect estimates [risk ratio (RR), hazard ratio (HR), or odds ratio (OR)] and 95% confidence intervals (CIs) for comparisons of different levels of OSA versus lowest OSA level. All case-control studies were excluded because various confounding factors could bias the results.

Data Collection and Quality Assessment

The data collected included the first author’s name, publication year, country, sample size, mean age at baseline, percentage of male patients, body mass index

For peer review only

(BMI), disease status, assessment of OSA, follow-up duration, effect estimate and its 95% CI, reported endpoints, and covariates in the fully adjusted model. For studies that reported several multivariable adjusted RRs, the effect estimate that was maximally adjusted for potential confounders was selected.

The Newcastle–Ottawa Scale (NOS), which is quite comprehensive and has been partially validated for evaluating the quality of observational studies in the meta-analysis, was used to evaluate the methodological quality[10]. The NOS is based on the following three subscales: selection (4 items), comparability (1 item), and outcome (3 items). A “star system” (range, 0–9) was developed for assessment (Table 1). The data extraction and quality assessment were conducted independently by two authors. Information was examined and adjudicated independently by an additional author referring to the original studies.

Statistical analysis

The relationship between OSA and risk of vascular outcomes or total mortality based on the effect estimate (OR, RR, or HR) and its 95% CI was examined in each study. To analyze the trend between OSA levels and vascular outcomes or total mortality risk, a semi-parametric method was first used to evaluate the association between mild OSA [apnea–hypopnea index (AHI): 5–15], moderate OSA (AHI: 15–30), severe OSA (AHI > 30), and the risk of vascular outcomes or total mortality[11]. Each category of AHI levels was established based on its calculated median, and the control category was composed of participants with lowest AHI or

For peer review only

normal participants in each study. Furthermore, when more than one median of AHI levels in each study was classified into one of these three categories, the fixed-effects model was used to calculate their summary RRs and 95% CIs for effect estimates of each category[12]. The random-effects model was then used to calculate summary RRs and 95% CIs for mild, moderate, and severe OSA versus normal[13]. Finally, the relative risk ratios and the corresponding 95% CIs were estimated using specific RRs and 95% CIs after considering the country, mean age, gender, BMI, disease status, and duration of the follow-up period[14].

Heterogeneity between studies was investigated using the Q-statistic, and *P* values <0.10 was considered as indicative of significant heterogeneity[15 16]. Subgroup analyses were conducted for mild, moderate, and severe OSA, and the risk of MACEs based on the country, mean age, gender, BMI, disease status, and duration of the follow-up period. A sensitivity analysis was also performed by removing each individual study from the meta-analysis[17]. Several methods were used to check for potential publication bias. Visual inspections of funnel plots for MACEs were conducted. The Egger[18] and Begg[19] tests were also used to statistically assess publication bias for MACEs. All reported *P* values were two sided, and *P* values <0.05 were considered statistically significant for all included studies. Statistical analyses were performed using the STATA software (version 12.0; Stata Corporation, TX, USA).

Results

For peer review only

Literature Search

The results of study-selection process are shown in Figure 1. An initial electronic search yielded 3282 articles, of which 3236 duplicates and irrelevant studies were excluded, and 46 potentially eligible studies were selected. After detailed evaluations, 16 prospective studies were selected for the final meta-analysis[20-35]. No new studies qualified for inclusion after a manual search of the reference lists of these studies. The general characteristics of the included studies are presented in Table 1.

Study Characteristics

Sixteen studies with a total of 36,363 individuals qualified for this study. The follow-up period for participants was 2.9–18.0 years, while 73–6294 individuals were included in each study. Eight studies were conducted in the USA, four in Spain, one in Sweden, one in Portugal, one in Hungary, and one in Canada. Furthermore, 11 studies reported healthy participants, and the remaining 5 studies reported participants with different diseases. The mean BMI ranged from 26.8 to 34.0 kg/m^2. Fourteen studies used polysomnography (PSG), and the remaining one study used limited PSG to assess the levels of OSA. The study quality was assessed using the NOS (Table 1). Overall, one study had a score of 9, six studies had a score of 8, seven studies had a score of 7, and the remaining two studies had a score of 6.

For peer review only

Table 1. Baseline characteristic of studies included in the systematic review and meta-analysis

Study	Country	Sample size	Mean age	Percentage male (%)	BMI	Disease status	Assessment OSA	Follow-up duration (year)	Reported outcomes	Adjusted factors	NOS score
Mooe et al 2000[20]	Sweden	408	59.1	58.4	27.0	CAD	Limited PSG	5.1	CHD, stroke, total mortality	Age, sex, BMI, hypertension, DM, LVF, and coronary intervention	7
Gottlieb et al 2010[21]	USA	4422	62.4	43.5	28.2	Healthy	PSG	8.7	CHD, HF	Age, race, BMI, smoking, DM, SBP, DBP, TC, HDL-C, lipid-lowering medications, and antihypertensive medications	8

For peer review only

Campos-Rodriguez et al 2012[22]	Spain	1116	56.1	0.0	36.6	Healthy	PSG	6.0	Cardiac death	Age, BMI, DM, hypertension, and previous CVD	8
Marin et al 2005[23]	Spain	1729	49.9	100	28.7	Healthy	PSG	10.1	Cardiac death and CHD	Age, diagnostic group, presence of CVD, DM, hypertension, lipid disorders, smoking, alcohol, SBP DBP, blood glucose, TC, TG, and use of antihypertensive, lipid-lowering and antidiabetic drugs	9
Young et al 2008[24]	USA	1522	48.0	55.0	28.6	Healthy	PSG	18.0	Cardiac death, total mortality,	Age, age-squared, sex, BMI, and BMI-squared	8

For peer review only

									and CHD		
Redline et al 2010[25]	USA	5422	62.9	45.4	27.8	Healthy	PSG	8.7	Stroke	Age, BMI, race, smoking, SBP, DM, and antihypertensive medications	8
Arzt et al 2005[26]	USA	1189	47.0	55.0	30.0	Healthy	PSG	4.0	Stroke	Age, sex, and BMI	7
Punjabi et al 2008[27]	USA	6294	62.5	47.0	27.8	Healthy	PSG	8.2	CHD, total mortality	Age, sex, race, BMI, SBP, DBP, smoking, prevalent hypertension, DM, and CVD	8
Shah et al 2010[28]	USA	1436	59.7	69.4	32.9	Healthy	PSG	2.9	CHD, cardiac death	Age, race, sex, smoking, alcohol, BMI, AF, DM, hypertension,	7

For peer review only

										and hyperlipidemia	
Yaggi et al 2005[29]	USA	1022	60.2	71.3	32.8	Healthy	PSG	3.4	Stroke and total mortality	Age, sex, race, smoking, alcohol, BMI, DM, hyperlipidemia, AF, and hypertension	8
Martı´nez-Garcı´a et al 2009[30]	Spain	166	73.3	59.0	28.1	Ischemic Stroke	PSG	5.0	Total mortality	Age, sex, Barthel index, AHI, and CPAP treatment groups, previous stroke or TIA, diabetes, hypercholesterolemia, BMI, smoking, arterial hypertension, atrial fibrillation, significant carotid stenosis, and fibrinogen levels	7

For peer review only

Munoz et al 2006[31]	Spain	1034	79.8	57.0	26.8	Healthy	PSG	6.0	Stroke	Sex	7
Leão et al 2016[32]	Portugal	73	62.4	75.0	27.6	Acute coronary syndrome	PSG	6.3	CHD	Sex	7
Fornadi et al 2014[33]	Hungary	100	51.0	56.8	26.8	Kidney transplant recipients	PSG	6.3	Total mortality	Unadjusted	6
Kendzerska et al 2014[34]	Canada	10149	49.9	62.0	30.1	Healthy	PSG	5.7	Total mortality	Traditional CV risk factors	7

For peer review only

Won et al 2013[35]	USA	281	65.0	98.0	34.0	Ischemic heart disease and myocardial injury	PSG	4.1	Total mortality	NA	6

AF, atrial fibrillation; BMI, body mass index; CAD, coronary artery disease; CHD, coronary heart disease; CPAP, continuous positive airway pressure; CV, cardiovascular; DBP, diastolic blood pressure; DM, diabetes mellitus; HDL-C, high-density lipoprotein cholesterol; HF, heart failure; LVF, left ventricular function; OSA, obstructive sleep apnea; PSG, polysomnography; SBP, systolic blood pressure; TC, total cholesterol; TG, triglyceride; TIA, transient ischemic attack.

For peer review only

OSA and MACEs risk

The summary RRs showed that mild OSA was not associated with MACEs (RR: 0.98; 95% CI: 0.87–1.11; *P* = 0.741; Fig. 2 and Table 2). Furthermore, the pooled analysis results for moderate and severe OSA indicated that they had a harmful effect on the risk of MACEs (moderate: RR: 1.16; 95% CI: 1.01–1.33; *P* = 0.034; Fig. 3 and Table 2; severe: RR: 2.04; 95% CI: 1.56–2.66; *P* < 0.001; Fig. 4 and Table 2). Subgroup analysis for MACEs was conducted to minimize heterogeneity among the included studies and evaluate the relationship of OSA and MACEs in specific subpopulations (Table 3). Overall, participants with moderate OSA were associated with an increased risk of MACEs if individuals did not have other diseases (RR: 1.16; 95%CI: 1.01–1.33; *P* = 0.034). Furthermore, no significant association was found between severe OSA and MACEs if the study included only women (RR: 1.98; 95% CI: 0.64–6.06; *P* = 0.234); in other subsets, severe OSA was associated with increased risk of MACEs (Table 3). Finally, no evidence of a factor-specific difference was found in the RR for MACEs among participants with OSA compared with controls (Table 3).

Table 2. Summary of the relative risks of all outcomes evaluated

Outcomes	Mild OSA	*P* value	Moderate OSA	*P* value	Severe OSA	*P* value
MACEs	0.98 (0.87–1.11)	0.741	1.16 (1.01–1.33)	0.034	2.04 (1.56–2.66)	<0.001
CHD	1.25 (0.95–1.66)	0.117	1.38 (1.04–1.83)	0.026	1.63 (1.18–2.26)	0.003

For peer review only

Stroke	1.29 (0.69–2.41)	0.424	1.35 (0.82–2.23)	0.245	2.15 (1.42–3.24)	<0.001
Cardiac death	1.80 (0.68–4.76)	0.236	1.11 (0.53–2.35)	0.781	2.96 (1.45–6.01)	0.003
Total mortality	1.26 (0.77–2.07)	0.354	1.04 (0.60–1.79)	0.895	1.54 (1.21–1.97)	<0.001
Heart failure	1.02 (0.78–1.34)	0.868	1.07 (0.74–1.54)	0.719	1.44 (0.94–2.21)	0.097

CHD, Coronary heart disease; MACE, major cardiovascular event; OSA, obstructive sleep apnea.

Table 3. Subgroup analyses for MACEs

Variable	Subgroup	Mild OSA	*P* value	Moderate OSA	*P* value	Severe OSA	*P* value
Country	USA	1.00 (0.85–1.17)	0.977	1.14 (0.99–1.32)	0.064	**1.90 (1.35–2.67)**	**<0.001**
	Other	1.02 (0.19–5.52)	0.982	1.44 (0.83–2.50)	0.198	**2.35 (1.52–3.65)**	**<0.001**
	USA vs other	0.98 (0.18–5.32)	0.982	0.79 (0.45–1.40)	0.422	0.81 (0.46–1.41)	0.453
Mean age	≥60	0.96 (0.86–1.08)	0.540	1.13 (0.97–1.33)	0.117	**1.78 (1.23–2.57)**	**0.002**
	<60	1.40 (0.73–2.70)	0.315	1.51 (0.94–2.41)	0.086	**2.31 (1.64–3.24)**	**<0.001**
	≥60 vs <60	0.69 (0.35–1.33)	0.265	0.75 (0.46–1.23)	0.252	0.77 (0.47–1.27)	0.309
Gender	Male	0.92 (0.73–1.15)	0.455	1.10 (0.85–1.42)	0.449	**1.81 (1.14–2.89)**	**0.012**
	Female	1.97 (0.47–8.25)	0.353	1.36 (0.67–2.76)	0.399	1.98 (0.64–6.06)	0.234
	Male vs female	0.47 (0.11–1.99)	0.304	0.81 (0.38–1.72)	0.581	0.91 (0.27–3.08)	0.885

For peer review only

BMI	≥30	1.75 (0.88–3.49)	0.111	1.70 (0.94–3.07)	0.079	**2.72 (1.80–4.10)**	**<0.001**
	<30	0.96 (0.86–1.07)	0.449	1.14 (0.99–1.31)	0.078	**1.80 (1.36–2.38)**	**<0.001**
	≥30 vs <30	1.82 (0.91–3.66)	0.092	1.49 (0.81–2.74)	0.198	1.51 (0.92–2.49)	0.104
Disease statues	Healthy	1.00 (0.85–1.17)	0.977	**1.16 (1.01–1.33)**	**0.034**	**2.12 (1.53–2.94)**	**<0.001**
	Other	1.02 (0.19–5.52)	0.982	–	-	**1.96 (1.01–3.81)**	**0.047**
	Healthy vs Other	0.98 (0.18–5.32)	0.982	–	-	1.08 (0.52–2.27)	0.835
Follow-up duration	≥6	0.96 (0.86–1.07)	0.449	1.14 (0.99–1.31)	0.064	**2.06 (1.43–2.95)**	**<0.001**
	<6	1.75 (0.88–3.49)	0.111	1.74 (0.87–3.49)	0.120	**2.10 (1.39–3.17)**	**<0.001**
	≥6 vs <6	0.55 (0.27–1.10)	0.092	0.66 (0.32–1.33)	0.242	0.98 (0.57–1.70)	0.945

OSA, obstructive sleep apnea.

OSA and CHD risk

The pooled data of meta-analysis showed that mild OSA was not associated with the risk of CHD (RR: 1.25; 95% CI: 0.95–1.66; *P* = 0.117; Table 2), whereas moderate OSA (RR: 1.38; 95% CI: 1.04–1.83; *P* = 0.026; Table 2) and severe OSA (RR: 1.63; 95% CI: 1.18–2.26; *P* = 0.003; Table 2) were associated with a significantly increased risk of CHD. Stratified analyses according to gender was conducted for different levels of OSA versus normal group, and it was found that patients with severe OSA had significantly increased risk of CHD in men (RR: 1.65;

For peer review only

95% CI: 1.06–2.57; *P* = 0.027). No other significant differences were detected (Table 4).

Table 4. Gender difference for other outcomes

Outcome	Subgroup	Mild OSA	*P* value	Moderate OSA	*P* value	Severe OSA	*P* value
CHD	Men	0.93 (0.72–1.21)	0.596	1.09 (0.80–1.48)	0.582	**1.65 (1.06–2.57)**	**0.027**
	Women	1.92 (0.43–8.64)	0.394	1.51 (0.38–5.97)	0.559	1.10 (0.12–9.87)	0.933
	Men vs women	0.48 (0.11–2.22)	0.351	0.72 (0.18–2.96)	0.651	1.50 (0.16–14.22)	0.724
Stroke	Men	1.86 (0.67–5.14)	0.232	1.86 (0.70–4.95)	0.214	**2.86 (1.10–7.41)**	**0.031**
	Women	1.34 (0.76–2.36)	0.311	1.20 (0.67–2.15)	0.542	1.21 (0.65–2.25)	0.546
	Men vs women	1.39 (0.43–4.45)	0.581	1.55 (0.50–4.84)	0.451	2.36 (0.76–7.38)	0.138
Cardiac death	Men	–	–	1.15 (0.41–3.23)	0.791	**2.87 (1.13–7.27)**	**0.026**
	Women	–	–	0.94 (0.19–4.61)	0.939	3.71 (0.41–33.87)	0.245
	Men vs women	–	–	1.22 (0.18–8.17)	0.935	0.77 (0.07–8.49)	0.834
Total mortality	Men	–	–	–	–	**1.72 (1.22–2.43)**	**0.002**
	Women	–	–	–	–	**3.50 (1.23–9.97)**	**0.019**
	Men vs women	–	–	–	–	0.49 (0.16–1.48)	0.206
Heart	Men	0.88 (0.57–1.35)	0.561	1.13 (0.68–1.88)	0.639	1.58 (0.93–2.67)	0.088

For peer review only

failure	Women	1.13 (0.80–1.60)	0.493	1.01 (0.60–1.70)	0.970	1.19 (0.56–2.52)	0.650
	Men vs women	0.78 (0.45–1.35)	0.376	1.12 (0.54–2.32)	0.762	1.33 (0.53–3.33)	0.545

CHD, coronary heart disease; OSA, obstructive sleep apnea.

OSA and stroke risk

Pooled analysis results indicated no association between mild OSA (RR: 1.29; 95% CI: 0.69–2.41; P = 0.424) and moderate OSA (RR: 1.35; 95% CI: 0.82–2.23; P = 0.245) and stroke, whereas severe OSA was associated with an increased risk of stroke (RR: 2.15; 95% CI: 1.42–3.24; P < 0.001). Subgroup analysis on the basis of gender indicated that severe OSA had a harmful effect on the risk of stroke in men (RR: 2.86; 95% CI: 1.10–7.41; P = 0.031; Table 4).

OSA and cardiac death risk

The summary RRs showed that mild OSA (RR: 1.80; 95% CI: 0.68–4.76; P = 0.236) and moderate OSA (RR: 1.11; 95% CI: 0.53–2.35; P = 0.781) were not associated with cardiac death risk, whereas severe OSA significantly increased the risk of cardiac death (RR: 2.96; 95% CI: 1.45–6.01; P = 0.003; Table 2). Subgroup analysis showed that severe OSA was associated with an increased risk of cardiac death in men (RR: 2.87; 95% CI: 1.13–7.27; P = 0.026; Table 4).

OSA and total mortality risk

No significant association was found between mild OSA (RR: 1.26; 95% CI: 0.77–2.07; P = 0.354), moderate OSA (RR: 1.04; 95% CI: 0.60–1.79; P = 0.895), and

For peer review only

total mortality risk. However, severe OSA had a harmful impact on total mortality (RR: 1.54; 95% CI: 1.21–1.97; $P < 0.001$; Table 2). Stratified analysis suggested that severe OSA increased the risk of total mortality in men (RR: 1.72; 95% CI: 1.22–2.43; $P = 0.002$) and women (RR: 3.50; 95% CI: 1.23–9.97; $P = 0.019$; Table 4).

OSA and heart failure risk

The summary results indicated no significant differences between mild OSA (RR: 1.02; 95% CI: 0.78–1.34; $P = 0.868$), moderate OSA (RR: 1.07; 95% CI: 0.74–1.54; $P = 0.719$), severe OSA (RR: 1.44; 95% CI: 0.94–2.21; $P = 0.097$), and the risk of heart failure (Table 2). Subgroup analysis reported similar results compared with the overall analysis.

Publication bias

Review of the funnel plots could not rule out the potential publication bias for MACEs (Fig. 5). The Egger and Begg test results showed no evidence of publication bias for MACEs of mild OSA (*P* value for Egger: 0.132; *P* value for Begg: 0.221) and moderate OSA (*P* value for Egger: 0.052; *P* value for Begg: 0.452). Although the Begg test showed no evidence of publication bias for MACEs of severe OSA ($P = 0.118$), the Egger test showed potential evidence of publication bias for MACEs of severe OSA ($P < 0.001$). The conclusion did not change after adjustment for publication bias using the trim-and-fill method[36].

Discussion

The present study was based on prospective cohort studies and explored all possible

For peer review only

correlations between OSA and the outcomes of MACEs, CHD, stroke, cardiac death, total mortality, and heart failure. This large quantitative study included 36,363 individuals from 16 prospective cohort studies with a broad range of populations. The findings from the present meta-analysis suggested that mild OSA had no significant impact on the risk of vascular outcomes and total mortality, moderate OSA was associated with an increased risk of MACEs and CHD, and severe OSA had a harmful effect on the risk of MACEs, CHD, stroke, cardiac death, and total mortality.

A previous meta-analysis suggested that OSA was associated with stroke, but the relationship with ischemic heart disease and cardiovascular mortality needs further research[37]. However, this study could not illustrate the impact of different levels of OSA on the risk of serious cardiovascular outcomes. Further, Dong et al suggested that moderate-to-severe OSA significantly increased the risk of cardiovascular diseases, in particular, the risk of stroke[38]. Similarly, Ge et al indicated that severe OSA is a strong independent predictor of cardiovascular and all-cause mortality. CPAP treatment was associated with decreased cardiovascular mortality[39]. However, these two studies could not evaluate the association of OSA with the risk of vascular outcomes and total mortality in specific subpopulations. Finally, Wang et al suggested that severe OSA significantly increased the risk of CHD and stroke, and all-cause mortality. A positive association with CHD was observed for moderate OSA but not for mild OSA[40]. However, whether this relationship differs according to the characteristics of participants remains unclear. Therefore, a comprehensive meta-analysis of these prospective cohort studies was performed to evaluate any

For peer review only

possible correlates between OSA and vascular outcomes.

No significant difference was observed between mild OSA and the risk of vascular outcomes. However, several studies included in this study reported inconsistent results. Young et al suggested that mild OSA significantly increased the risk of CHD by 92%[24], whereas Punjabi et al indicated that mild OSA might have a harmful effect on the risk of CHD[27]. This might be because these two studies used healthy individuals as controls, which may make them more susceptible to acquired significant conclusion. Furthermore, most of these studies did not take into account potential confounders for the risk of cardiovascular disease. Moderate-to-severe OSA might play an important role in the risk of vascular outcomes. Shah et al concluded that OSA increased the risk of coronary events or death from cardiovascular causes[28]. Nearly all included studies reported adverse outcomes for severe OSA. Previous studies indicated that OSA was a cause of diabetes, which was an independent risk factor for MACEs. Multiple adjusted models might be biased as the adjusted variables are different, reflecting either mediation or confounding.

Subgroup analyses reported similar conclusions. Gender might have an impact on the relationship between OSA and CHD, stroke, or cardiac death, although the sex difference was not statistically significant. The possible reasons could be the lower prevalence of severe OSA in women and the later age of onset of OSA in women than in men. Furthermore, OSA in women always occurred after menopause. Physiological response to OSA is another reason for this nonsignificant difference. Finally, these conclusions might be unreliable because smaller cohorts were included in each subset.

For peer review only

Therefore, further large-scale studies were needed to verify this difference. Therefore, a relative result was given and a synthetic and comprehensive review was provided.

Three strengths of this study should be highlighted. First, only prospective studies were included, which eliminated selection and recall bias, and could be of concern in retrospective case–control studies. Second, the large sample size allowed us to quantitatively assess the association of OSA with the risk of vascular outcomes and mortality, and thus the findings were potentially more robust than those of any individual study. Third, the summary RRs were calculated to evaluate any potential difference between subsets according to the characteristics of participants.

The limitations of this study were as follows: (1) the adjusted models were different across the included studies, and these factors might have played an important role in the development of vascular outcomes; (2) in a meta-analysis of published studies, publication bias was an inevitable problem; and (3) the analysis used pooled data (individual data were not available), which restricted performing a more detailed relevant analysis and obtaining more comprehensive results.

The results of this study suggest that moderate-to-severe OSA might play an important role in the risk of vascular outcomes, especially for men. Future studies should focus on specific populations to analyze the gender difference to study the association between OSA and vascular outcomes.

Author Contributions:

Chengjuan Xie carried out the studies, participated in collecting data, and drafted the

For peer review only

manuscript. Ruolin Zhu performed the statistical analysis and participated in its design. Yanghua Tian, Kai Wang helped to draft the manuscript. All authors read and approved the final manuscript.

Conflict of interests: All authors declare that they have no conflict of interest.

Funding statement: This research was supported by the National Basic Research Program of China (nos. 973 Program 2015CB856405 and 2012CB720704) and the National Natural Science Foundation of China (nos. 31571149, 91432301, 81301176, and 81171273).

Data sharing statement : No additional data available.

For peer review only

References

1 Young T, Palta M, Dempsey J, et al. The occurrence of sleep-disordered breathing among middle-aged adults. *N Engl J Med* 1993;**328**(17):1230-5 doi: 10.1056/NEJM199304293281704published Online First: Epub Date]|.

2 Young T, Peppard PE, Taheri S. Excess weight and sleep-disordered breathing. *J Appl Physiol (1985)* 2005;**99**(4):1592-9 doi: 10.1152/japplphysiol.00587.2005published Online First: Epub Date]|.

3 Liu S, Lin Y, Liu X. Meta-Analysis of Association of Obstructive Sleep Apnea With Glaucoma. *J Glaucoma* 2016;**25**(1):1-7 doi: 10.1097/IJG.0000000000000357published Online First: Epub Date]|.

4 Leong WB, Jadhakhan F, Taheri S, et al. The Association between Obstructive Sleep Apnea on Diabetic Kidney Disease: A Systematic Review and Meta-Analysis. *Sleep* 2016;**39**(2):301-8 doi: 10.5665/sleep.5432published Online First: Epub Date]|.

5 Xu S, Wan Y, Xu M, et al. The association between obstructive sleep apnea and metabolic syndrome: a systematic review and meta-analysis. *BMC Pulm Med* 2015;**15**:105 doi: 10.1186/s12890-015-0102-3published Online First: Epub Date]|.

6 Kim Y, Koo YS, Lee HY, et al. Can Continuous Positive Airway Pressure Reduce the Risk of Stroke in Obstructive Sleep Apnea Patients? A Systematic Review and Meta-Analysis. *PLoS One* 2016;**11**(1):e0146317 doi: 10.1371/journal.pone.0146317published Online First: Epub Date]|.

For peer review only

7 Bratton DJ, Gaisl T, Wons AM, et al. CPAP vs Mandibular Advancement Devices and Blood Pressure in Patients With Obstructive Sleep Apnea: A Systematic Review and Meta-analysis. *JAMA* 2015;**314**(21):2280-93 doi: 10.1001/jama.2015.16303published Online First: Epub Date]|.

8 Nadeem R, Singh M, Nida M, et al. Effect of CPAP treatment for obstructive sleep apnea hypopnea syndrome on lipid profile: a meta-regression analysis. *J Clin Sleep Med* 2014;**10**(12):1295-302 doi: 10.5664/jcsm.4282published Online First: Epub Date]|.

9 Stroup DF, Berlin JA, Morton SC, et al. Meta-analysis of observational studies in epidemiology: a proposal for reporting. Meta-analysis Of Observational Studies in Epidemiology (MOOSE) group. *JAMA* 2000;**283**(15):2008-12

10 Wells G, Shea B, O'Connell D. The Newcastle-Ottawa Scale (NOS) for assessing the quality of nonrandomised studies in meta-analyses. *Ottawa Hospital Research Institute* 2009

11 Bradley TD, Floras JS. Obstructive sleep apnoea and its cardiovascular consequences. *Lancet* 2009;**373**(9657):82-93 doi: 10.1016/S0140-6736(08)61622-0published Online First: Epub Date]|.

12 DerSimonian R, Laird N. Meta-analysis in clinical trials. *Control Clin Trials* 1986;**7**(3):177-88

13 Ades AE, Lu G, Higgins JP. The interpretation of random-effects meta-analysis in decision models. *Med Decis Making* 2005;**25**(6):646-54 doi: 10.1177/0272989X05282643published Online First: Epub Date]|.

For peer review only

14 Huxley RR, Woodward M. Cigarette smoking as a risk factor for coronary heart disease in women compared with men: a systematic review and meta-analysis of prospective cohort studies. *Lancet* 2011;**378**(9799):1297-305 doi: 10.1016/S0140-6736(11)60781-2published Online First: Epub Date]|.

15 Deeks JJ, Higgins JP, Altman DG. *Analysing Data and Undertaking Meta‐Analyses*: John Wiley & Sons, Ltd, 2008.

16 Higgins JP, Thompson SG, Deeks JJ, et al. Measuring inconsistency in meta-analyses. *BMJ* 2003;**327**(7414):557-60 doi: 10.1136/bmj.327.7414.557published Online First: Epub Date]|.

17 Altman DG, Bland JM. Interaction revisited: the difference between two estimates. *BMJ* 2003;**326**(7382):219

18 Egger M, Davey Smith G, Schneider M, et al. Bias in meta-analysis detected by a simple, graphical test. *BMJ* 1997;**315**(7109):629-34

19 Begg CB, Mazumdar M. Operating characteristics of a rank correlation test for publication bias. *Biometrics* 1994;**50**(4):1088-101

20 Mooe T, Franklin KA, Wiklund U, et al. Sleep-disordered breathing and myocardial ischemia in patients with coronary artery disease. *Chest* 2000;**117**(6):1597-602

21 Gottlieb DJ, Yenokyan G, Newman AB, et al. Prospective study of obstructive sleep apnea and incident coronary heart disease and heart failure: the sleep heart health study. *Circulation* 2010;**122**(4):352-60 doi: 10.1161/CIRCULATIONAHA.109.901801published Online First: Epub

For peer review only

Date]|.

22 Campos-Rodriguez F, Martinez-Garcia MA, de la Cruz-Moron I, et al. Cardiovascular mortality in women with obstructive sleep apnea with or without continuous positive airway pressure treatment: a cohort study. *Ann Intern Med* 2012;**156**(2):115-22 doi: 10.7326/0003-4819-156-2-201201170-00006published Online First: Epub Date]|.

23 Marin JM, Carrizo SJ, Vicente E, et al. Long-term cardiovascular outcomes in men with obstructive sleep apnoea-hypopnoea with or without treatment with continuous positive airway pressure: an observational study. *Lancet* 2005;**365**(9464):1046-53 doi: 10.1016/S0140-6736(05)71141-7published Online First: Epub Date]|.

24 Young T, Finn L, Peppard PE, et al. Sleep disordered breathing and mortality: eighteen-year follow-up of the Wisconsin sleep cohort. *Sleep* 2008;**31**(8):1071-8

25 Redline S, Yenokyan G, Gottlieb DJ, et al. Obstructive sleep apnea-hypopnea and incident stroke: the sleep heart health study. *Am J Respir Crit Care Med* 2010;**182**(2):269-77 doi: 10.1164/rccm.200911-1746OCpublished Online First: Epub Date]|.

26 Arzt M, Young T, Finn L, et al. Association of sleep-disordered breathing and the occurrence of stroke. *Am J Respir Crit Care Med* 2005;**172**(11):1447-51 doi: 10.1164/rccm.200505-702OCpublished Online First: Epub Date]|.

For peer review only

27 Punjabi NM, Newman AB, Young TB, et al. Sleep-disordered breathing and cardiovascular disease: an outcome-based definition of hypopneas. *Am J Respir Crit Care Med* 2008;**177**(10):1150-5 doi: 10.1164/rccm.200712-1884OCpublished Online First: Epub Date]|.

28 Shah NA, Yaggi HK, Concato J, et al. Obstructive sleep apnea as a risk factor for coronary events or cardiovascular death. *Sleep Breath* 2010;**14**(2):131-6 doi: 10.1007/s11325-009-0298-7published Online First: Epub Date]|.

29 Yaggi HK, Concato J, Kernan WN, et al. Obstructive sleep apnea as a risk factor for stroke and death. *N Engl J Med* 2005;**353**(19):2034-41 doi: 10.1056/NEJMoa043104published Online First: Epub Date]|.

30 Martinez-Garcia MA, Soler-Cataluna JJ, Ejarque-Martinez L, et al. Continuous positive airway pressure treatment reduces mortality in patients with ischemic stroke and obstructive sleep apnea: a 5-year follow-up study. *Am J Respir Crit Care Med* 2009;**180**(1):36-41 doi: 10.1164/rccm.200808-1341OCpublished Online First: Epub Date]|.

31 Munoz R, Duran-Cantolla J, Martinez-Vila E, et al. Severe sleep apnea and risk of ischemic stroke in the elderly. *Stroke* 2006;**37**(9):2317-21 doi: 10.1161/01.STR.0000236560.15735.0fpublished Online First: Epub Date]|.

32 Leao S, Conde B, Fontes P, et al. Effect of Obstructive Sleep Apnea in Acute Coronary Syndrome. *Am J Cardiol* 2016;**117**(7):1084-7 doi: 10.1016/j.amjcard.2015.12.053published Online First: Epub Date]|.

33 Fornadi K, Ronai KZ, Turanyi CZ, et al. Sleep apnea is not associated with worse

For peer review only

outcomes in kidney transplant recipients. *Sci Rep* 2014;**4**:6987 doi: 10.1038/srep06987published Online First: Epub Date]|.

34 Kendzerska T, Gershon AS, Hawker G, et al. Obstructive sleep apnea and risk of cardiovascular events and all-cause mortality: a decade-long historical cohort study. *PLoS Med* 2014;**11**(2):e1001599 doi: 10.1371/journal.pmed.1001599published Online First: Epub Date]|.

35 Won CH, Chun HJ, Chandra SM, et al. Severe obstructive sleep apnea increases mortality in patients with ischemic heart disease and myocardial injury. *Sleep Breath* 2013;**17**(1):85-91 doi: 10.1007/s11325-012-0653-ypublished Online First: Epub Date]|.

36 Duval S, Tweedie R. A Nonparametric "Trim and Fill" Method of Accounting for Publication Bias in Meta-Analysis. *Journal of the American Statistical Association* 2000;**95**(449):89-98

37 Loke YK, Brown JW, Kwok CS, et al. Association of obstructive sleep apnea with risk of serious cardiovascular events: a systematic review and meta-analysis. *Circ Cardiovasc Qual Outcomes* 2012;**5**(5):720-8 doi: 10.1161/CIRCOUTCOMES.111.964783published Online First: Epub Date]|.

38 Dong JY, Zhang YH, Qin LQ. Obstructive sleep apnea and cardiovascular risk: meta-analysis of prospective cohort studies. *Atherosclerosis* 2013;**229**(2):489-95 doi: 10.1016/j.atherosclerosis.2013.04.026published Online First: Epub Date]|.

39 Ge X, Han F, Huang Y, et al. Is obstructive sleep apnea associated with

For peer review only

cardiovascular and all-cause mortality? *PLoS One* 2013;**8**(7):e69432 doi: 10.1371/journal.pone.0069432published Online First: Epub Date]|.

40 Wang X, Ouyang Y, Wang Z, et al. Obstructive sleep apnea and risk of cardiovascular disease and all-cause mortality: a meta-analysis of prospective cohort studies. *Int J Cardiol* 2013;**169**(3):207-14 doi: 10.1016/j.ijcard.2013.08.088published Online First: Epub Date]|.

For peer review only

Figure legends:

Figure 1. Study selection process

Figure 2. Association between mild OSA and MACEs

Figure 3. Association between moderate OSA and MACEs

Figure 4. Association between severe OSA and MACEs

Figure 5. Funnel plots

For peer review only

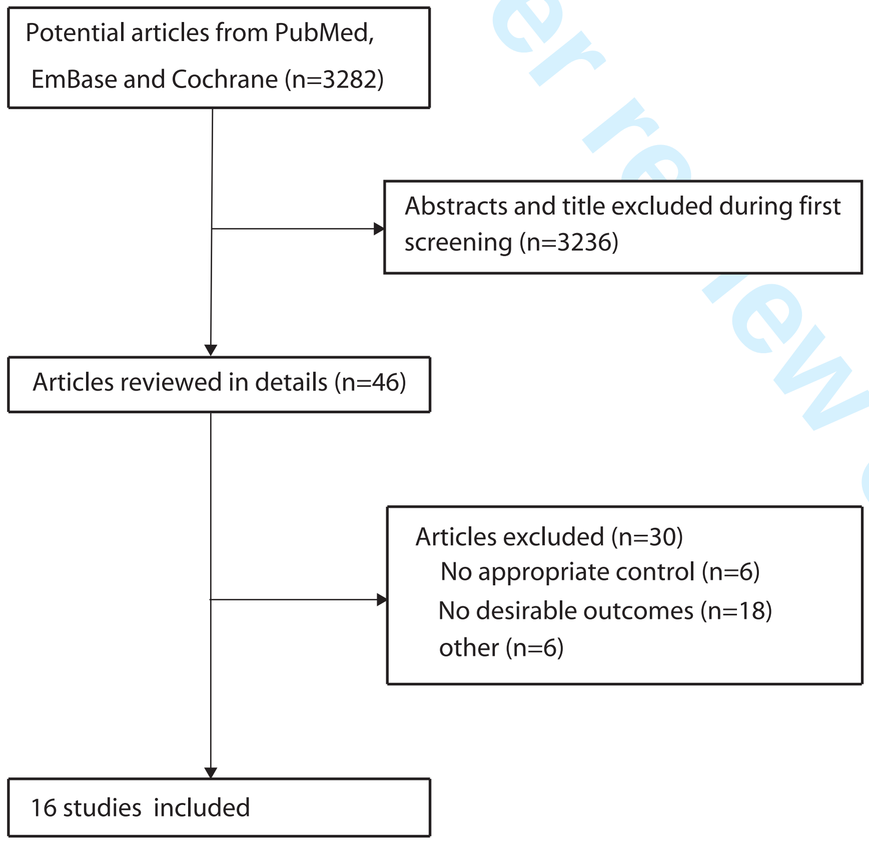

For peer review only

For peer review only

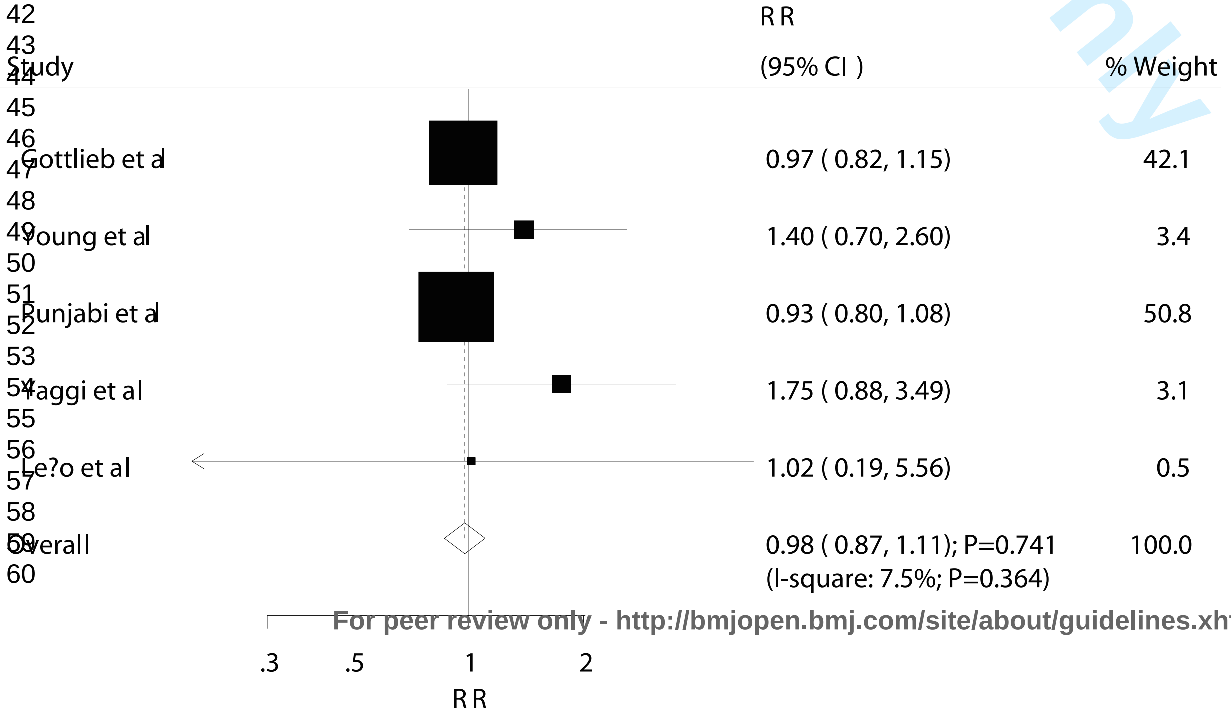

For peer review only

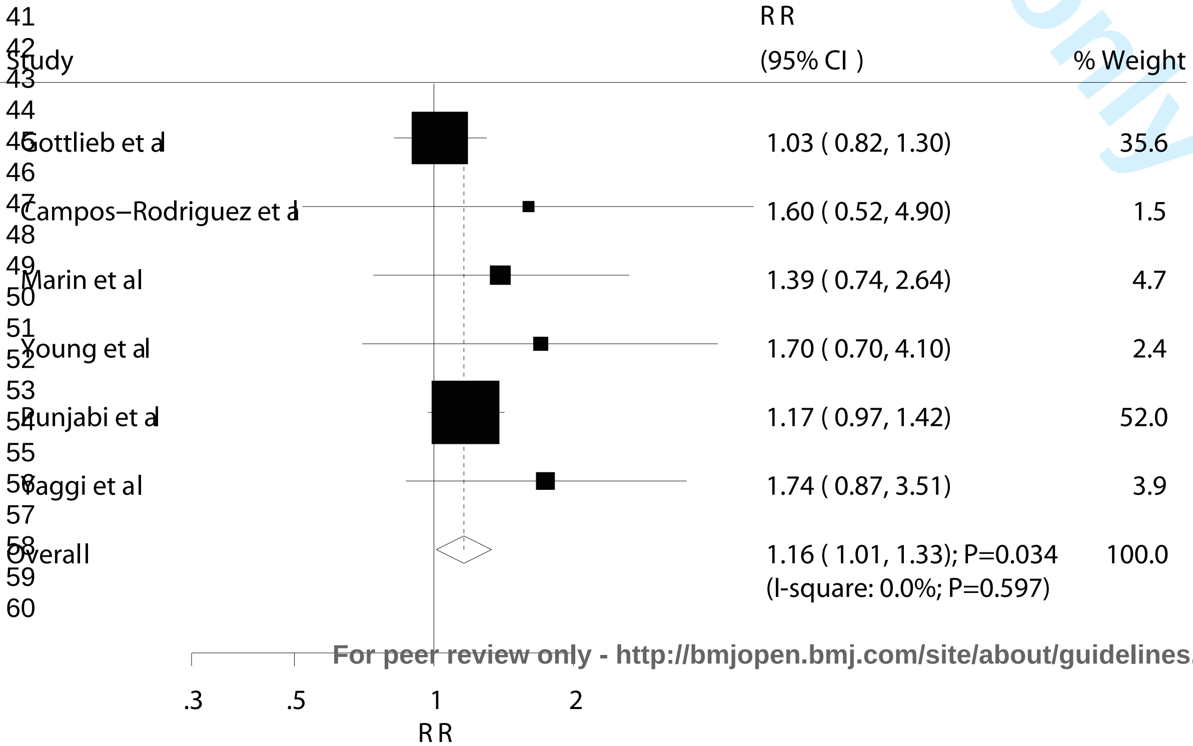

For peer review only

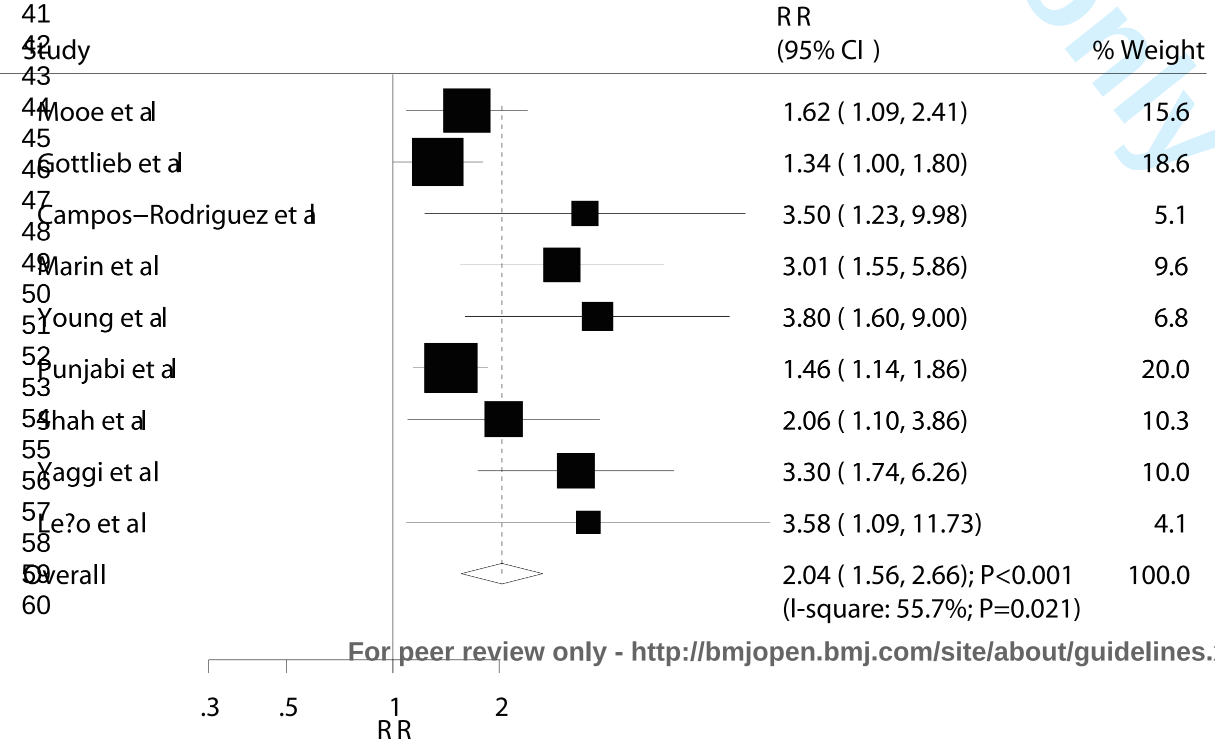

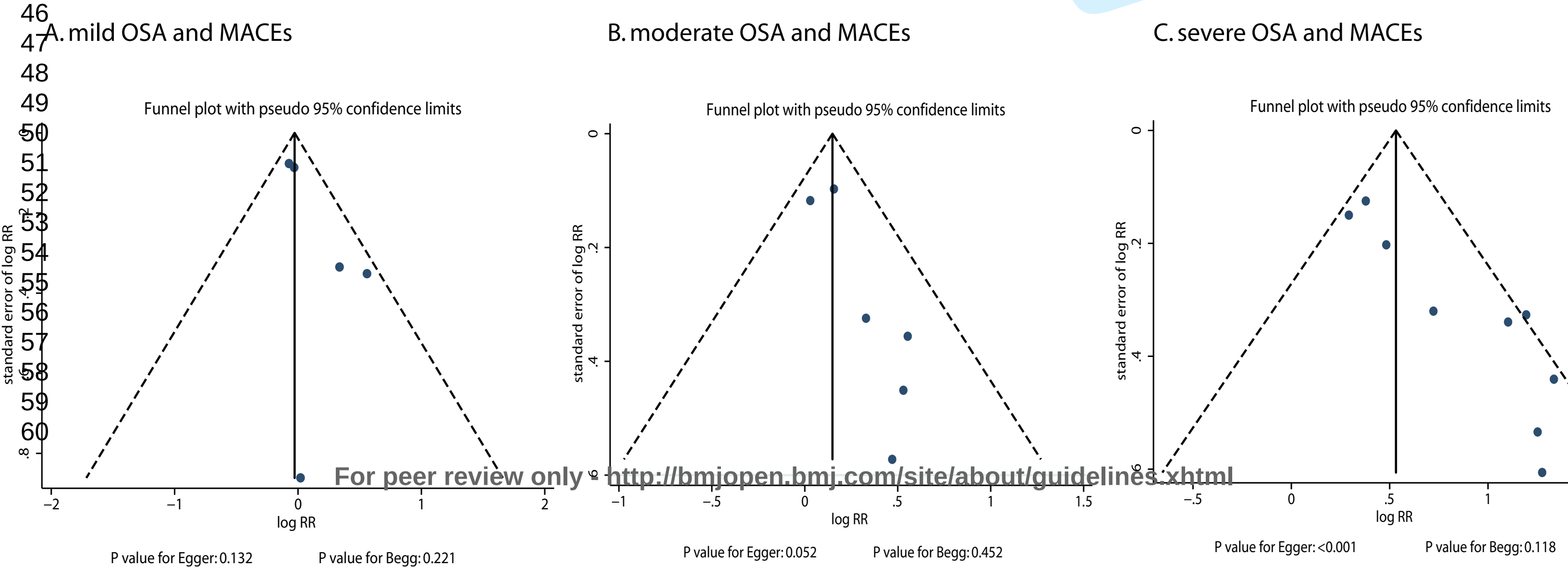

For peer review only

Title:

	**Search strategy**
**#1**	"Sleep Apnea, Obstructive" [Mesh] OR "OSA" [All fields] OR "OHS" [All fields]
**#2**	Apneas, Obstructive Sleep OR Obstructive Sleep Apneas OR Sleep Apneas, Obstructive OR Obstructive Sleep Apnea Syndrome OR Obstructive Sleep Apnea OR OSAHS OR Syndrome, Sleep Apnea, Obstructive OR Sleep Apnea Syndrome, Obstructive OR Apnea, Obstructive Sleep OR Sleep Apnea Hypopnea Syndrome OR Syndrome, Obstructive Sleep Apnea OR Upper Airway Resistance Sleep Apnea Syndrome OR Syndrome, Upper Airway Resistance, Sleep Apnea OR Hypoventilation Syndrome, Obesity OR Syndrome, Obesity Hypoventilation OR Pickwickian Syndrome OR Syndrome, Pickwickian OR Obesity-Hypoventilation Syndrome
**#3**	"Sleep Apnea Syndromes" [Mesh] OR "SAS" [All fields]
**#4**	Apnea Syndrome, Sleep OR Apnea Syndromes, Sleep OR Sleep Apnea Syndrome OR Apnea, Sleep OR Apneas, Sleep OR Sleep Apnea OR Sleep Apneas OR Sleep Hypopnea OR Hypopnea, Sleep OR Hypopneas, Sleep OR Sleep Hypopneas OR Sleep-Disordered Breathing OR Breathing, Sleep-Disordered OR Sleep Disordered Breathing OR Sleep Apnea, Mixed Central and Obstructive OR Mixed Central and Obstructive Sleep Apnea OR Sleep Apnea, Mixed OR Mixed Sleep Apnea OR Mixed Sleep Apneas OR Sleep Apneas, Mixed OR Hypersomnia with Periodic Respiration
**#5**	"Sleep Apnea, Central" [Mesh] OR "CSA"[All fields]
**#6**	Apneas, Central Sleep OR Central Sleep Apneas OR Sleep Apneas, Central OR Apnea, Central OR Apneas, Central OR Central Apnea OR Central Apneas OR Apnea, Central Sleep OR Apnea, Sleep, Central OR Sleep Apnea, Lethal Central OR Central Sleep Apnea OR Central Sleep Apnea Syndrome OR Central Sleep Disordered Breathing OR Hypoventilation, Central Alveolar OR Alveolar Hypoventilation, Central OR Alveolar Hypoventilations, Central OR Central Alveolar Hypoventilation OR Hypoventilations, Central Alveolar OR Ondine Syndrome OR Sleep-Disordered Breathing, Central OR Breathing, Central Sleep-Disordered OR Breathings, Central Sleep-Disordered OR Central Sleep-Disordered Breathing OR Central Sleep-Disordered Breathings OR Sleep Disordered Breathing, Central OR Sleep-Disordered Breathings, Central OR Central Alveolar Hypoventilation Syndrome OR Central Sleep Apnea, Secondary OR Secondary Central Sleep Apnea OR Sleep Apnea, Newborn, Primary OR Primary Sleep Apneas of Newborn OR Newborn Primary Sleep Apneas OR Central Sleep Apnea, Primary OR Primary Central Sleep Apnea
**#7**	"Continuous Positive Airway Pressure" [Mesh] OR "CPAP" [All fields] OR "Continuous Positive Airway Pressure/therapy" [Mesh]
**#8**	CPAP Ventilation OR Ventilation, CPAP OR Biphasic Continuous Positive Airway Pressure OR Bilevel Continuous Positive Airway Pressure OR Nasal Continuous Positive Airway Pressure OR nCPAP Ventilation OR Ventilation, nCPAP OR Airway Pressure Release Ventilation OR APRV Ventilation Mode OR APRV Ventilation Modes OR Ventilation Mode, APRV OR Ventilation Modes, APRV

For peer review only

**#9**	#1 OR #2 OR #3 OR #4 OR #5 OR #6 OR #7 OR #8
#10	"Cardiovascular System" [Mesh]
#11	"Death" [Mesh] OR Determination of Death OR Near-Death Experience OR Cardiac Death OR Death, Cardiac OR "Mortality" [Mesh] OR "mortality" [All fields] OR "Disease" [Mesh] OR "disease*"
**#12**	#10 AND #11
**#13**	"Cardiovascular Diseases" [Mesh] OR "CVD" OR Cardiovascular Disease OR Disease, Cardiovascular OR Diseases, Cardiovascular
**#14**	"Myocardial Infarction" [Mesh] OR "MI" OR Infarction, Myocardial OR Infarctions, Myocardial OR Myocardial Infarctions OR Cardiovascular Stroke OR Cardiovascular Strokes OR Stroke, Cardiovascular OR Strokes, Cardiovascular OR Heart Attack OR Heart Attacks OR Myocardial Infarct OR Infarct, Myocardial OR Infarcts, Myocardial OR Myocardial Infarcts
**#15**	"Angina Pectoris" [Mesh] OR "Angina, Stable" [Mesh] OR "Microvascular Angina" [Mesh] OR "Angina, Unstable" [Mesh] OR Stenocardia OR Stenocardias OR Angor Pectoris OR "angina" [All fields] OR "Coronary Artery Disease" [Mesh] OR "CAD" OR "ischemic heart disease" [All fields] OR "Heart Failure" [Mesh] OR "Heart Failure, Diastolic" [Mesh] OR "Heart Failure, Systolic" [Mesh]
**#16**	"Cerebrovascular Disorders" [Mesh] OR "cerebrovascular" [All fields] OR "stroke*"
**#17**	"Death" [Mesh] OR Determination of Death OR Near-Death Experience OR Cardiac Death OR Death, Cardiac OR "Mortality" [Mesh] OR "mortality" [All fields] OR "mortality*"
**#18**	#12 OR #13 OR #14 OR #15 OR #16 OR #17
**#19**	"Prospective Studies" [Mesh] OR "Cohort Studies" [Mesh] OR "Follow-Up Studies" [Mesh] OR "prospective study" OR "cohort study" OR "follow-up study"
**#20**	#9 AND #18 AND #19

For peer review only

STROBE 2007 (v4) checklist of items to be included in reports of observational studies in epidemiology*
Checklist for cohort, case-control, and cross-sectional studies (combined)

Section/Topic	Item #	Recommendation	Reported on page #
**Title and abstract**	1	(*a*) Indicate the study's design with a commonly used term in the title or the abstract	1
		(*b*) Provide in the abstract an informative and balanced summary of what was done and what was found	2
**Introduction**			3–4
Background/rationale	2	Explain the scientific background and rationale for the investigation being reported	3
Objectives	3	State specific objectives, including any pre-specified hypotheses	4
**Methods**			4–7
Study design	4	Present key elements of study design early in the paper	4
Setting	5	Describe the setting, locations, and relevant dates, including periods of recruitment, exposure, follow-up, and data collection	
Participants	6	(*a*) *Cohort study*—Give the eligibility criteria, and the sources and methods of selection of participants. Describe methods of follow-up<br>*Case-control study*—Give the eligibility criteria, and the sources and methods of case ascertainment and control selection. Give the rationale for the choice of cases and controls<br>*Cross-sectional study*—Give the eligibility criteria, and the sources and methods of selection of participants	
		(*b*) *Cohort study*—For matched studies, give matching criteria and number of exposed and unexposed<br>*Case-control study*—For matched studies, give matching criteria and the number of controls per case	
Variables	7	Clearly define all outcomes, exposures, predictors, potential confounders, and effect modifiers. Give diagnostic criteria, if applicable	
Data sources/ measurement	8*	For each variable of interest, give sources of data and details of methods of assessment (measurement). Describe comparability of assessment methods if there is more than one group	*4*
Bias	9	Describe any efforts to address potential sources of bias	
Study size	10	Explain how the study size was arrived at	
Quantitative variables	11	Explain how quantitative variables were handled in the analyses. If applicable, describe which groupings were chosen and why	5–6
Statistical methods	12	(*a*) Describe all statistical methods, including those used to control for confounding	6
		(*b*) Describe any methods used to examine subgroups and interactions	6
		(*c*) Explain how missing data were addressed	7
		(*d*) *Cohort study*—If applicable, explain how loss to follow-up was addressed<br>*Case-control study*—If applicable, explain how matching of cases and controls was addressed	7

For peer review only

		Cross-sectional study—If applicable, describe analytical methods taking account of sampling strategy	
		(*e*) Describe any sensitivity analyses	
**Results**			7–20
Participants	13*	(a) Report numbers of individuals at each stage of study—eg numbers potentially eligible, examined for eligibility, confirmed eligible, included in the study, completing follow-up, and analysed	
		(b) Give reasons for non-participation at each stage	
		(c) Consider use of a flow diagram	
Descriptive data	14*	(a) Give characteristics of study participants (eg demographic, clinical, social) and information on exposures and potential confounders	
		(b) Indicate number of participants with missing data for each variable of interest	
		(c) *Cohort study*—Summarise follow-up time (eg, average and total amount)	
Outcome data	15*	*Cohort study*—Report numbers of outcome events or summary measures over time	
		Case-control study—Report numbers in each exposure category, or summary measures of exposure	
		Cross-sectional study—Report numbers of outcome events or summary measures	
Main results	16	(*a*) Give unadjusted estimates and, if applicable, confounder-adjusted estimates and their precision (eg, 95% confidence interval). Make clear which confounders were adjusted for and why they were included	
		(*b*) Report category boundaries when continuous variables were categorized	
		(*c*) If relevant, consider translating estimates of relative risk into absolute risk for a meaningful time period	
Other analyses	17	Report other analyses done—eg analyses of subgroups and interactions, and sensitivity analyses	
**Discussion**			21–23
Key results	18	Summarise key results with reference to study objectives	22
Limitations	19	Discuss limitations of the study, taking into account sources of potential bias or imprecision. Discuss both direction and magnitude of any potential bias	
Interpretation	20	Give a cautious overall interpretation of results considering objectives, limitations, multiplicity of analyses, results from similar studies, and other relevant evidence	
Generalisability	21	Discuss the generalisability (external validity) of the study results	23
**Other information**			
Funding	22	Give the source of funding and the role of the funders for the present study and, if applicable, for the original study on which the present article is based	24

*Give information separately for cases and controls in case-control studies and, if applicable, for exposed and unexposed groups in cohort and cross-sectional studies.

Note: An Explanation and Elaboration article discusses each checklist item and gives methodological background and published examples of transparent reporting. The STROBE checklist is best used in conjunction with this article (freely available on the Web sites of PLoS Medicine at http://www.plosmedicine.org/, Annals of Internal Medicine at http://www.annals.org/, and Epidemiology at http://www.epidem.com/). Information on the STROBE Initiative is available at www.strobe-statement.org.

For peer review only

BMJ Open

Association of obstructive sleep apnea with the risk of vascular outcomes and all-cause mortality: a meta-analysis

Journal:	*BMJ Open*
Manuscript ID	bmjopen-2016-013983.R1
Article Type:	Research
Date Submitted by the Author:	24-Apr-2017
Complete List of Authors:	Xie, Chengjuan; The First Affiliated Hospital of Anhui Medical University, Department of Neurology<br>Zhu, Ruolin; The First Affiliated Hospital of Anhui Medical University, Department of Neurology<br>Tian, Yanghua; The First Affiliated Hospital of Anhui Medical University, Department of Neurology<br>Wang, Kai; Department of Neurology
**Primary Subject Heading**:	Neurology
Secondary Subject Heading:	Cardiovascular medicine
Keywords:	meta-analysis, mortality, obstructive sleep apnea, vascular outcome

SCHOLARONE™
Manuscripts

For peer review only

Association of obstructive sleep apnea with the risk of vascular outcomes and all-cause mortality: a meta-analysis

Chengjuan Xie[1], Ruolin Zhu[1], Yanghua Tian[1,2], Kai Wang[1, 2, 3,*]

[1]Department of Neurology, The First Affiliated Hospital of Anhui Medical University, Hefei, China

[2]Collaborative Innovation Centre of Neuropsychiatric Disorders and Mental Health, Anhui Province, China

[3]Department of Medical Psychology, Anhui Medical University, Hefei, China

***Correspondence author:**

Kai Wang,

Department of Neurology, The First Affiliated Hospital of Anhui Medical University, Hefei, 230022,China;Collaborative Innovation Centre of Neuropsychiatric Disorders and Mental Health, Anhui Province, 230022,China;Department of Medical Psychology, Anhui Medical University, Hefei, 230022,China

Tel: +86-0551-62922418,

Fax:+86-021-64085875,

E-mail: xiecj2016@163.com.

Word count: 5459

For peer review only

Abstract

Objective: This study aimed to conduct a meta-analysis to explore and summarize the evidence regarding the association between obstructive sleep apnea (OSA) and the subsequent risk of vascular outcomes and all-cause mortality.

Methods: Electronic databases PubMed, Embase, and the Cochrane Library were searched to identify studies conducted through May 2016. Prospective cohort studies that reported effect estimates with 95% confidence intervals of major adverse cardiac events (MACEs), coronary heart disease (CHD), stroke, cardiac death, all-cause mortality, and heart failure for different levels versus the lowest level of OSA were included.

Results: A total of 16 cohort studies reporting data on 24,308 individuals were included. Of these, 11 studies reported healthy participants, and the remaining 5 studies reported participants with different diseases. Severe OSA was associated with a greater risk of MACEs ($P < 0.001$), CHD ($P = 0.003$), stroke ($P < 0.001$), cardiac death ($P = 0.003$), and all-cause mortality ($P < 0.001$); moderate OSA had a harmful impact on MACEs ($P = 0.034$) and CHD ($P = 0.026$); and no significant association was found between mild OSA and the risk of vascular outcomes or all-cause mortality ($P > 0.05$). Finally, no evidence of a factor-specific difference in the risk ratio for MACEs among participants with different levels of OSA compared with those with the lowest level of OSA was found.

Conclusions: Severe and moderate OSAs were associated with an increased risk of

For peer review only

vascular outcomes and all-cause mortality. This relationship might differ between genders. Therefore, further large-scale prospective studies are needed to verify this difference.

Key words: Meta-analysis; mortality, obstructive sleep apnea, vascular outcome

For peer review only

Article Summary:

Strengths and limitations of this study:

1. The degree of association of OSA with fatal and nonfatal cardiovascular diseases (CVDs) was gender specific.

2. It provided statistical evidence on the association of moderate-to-severe OSA with MACEs.

3. The peculiar study design assessed risk ratios as per patient characteristics.

4. Quantitative data were used to emphasize the association of OSA as a poignant factor with CVDs.

Introduction

Obstructive sleep apnea (OSA) affects 24% of middle-aged men and 9% of women in the United States, but daytime sleepiness was reported in 17% and 22% of these

subjects, respectively [1]. OSA is an increasingly prevalent condition characterized by repetitive obstruction of the upper airway during sleep accompanied by episodic hypoxia, arousal, and sleep fragmentation [2]. Previous studies suggested that OSA was associated with an increased risk of glaucoma, diabetic kidney disease, and metabolic syndrome [3-5]. However, data on the association between OSA and the risk of subsequent vascular outcomes and mortality are both limited and inconclusive. Furthermore, whether these relationships differ according to the characteristics of patients with OSA also needs to be verified.

Several meta-analyses have illustrated that continuous positive airway pressure (CPAP) interventions aimed at OSA may reduce the risk of cardiovascular outcomes. Kim et al. [6] showed that CPAP treatment for OSA was associated with a lower incidence of stroke and cardiac events. Furthermore, Bratton et al. [7] indicated that use of both CPAP and mandibular advancement devices was associated with a reduction in the blood pressure among patients with OSA. Nadeem et al. [8] suggested that CPAP treatment for OSA seemed to improve dyslipidemia (decrease in total cholesterol and low-density lipoprotein, and increase in high-density lipoprotein), whereas it did not appear to affect the triglyceride levels. These studies demonstrated that patients with OSA who received interventions had a reduced risk of cardiovascular diseases. Therefore, clarifying the relationship between OSA and vascular outcomes is particularly important as it has not been definitively determined. This study attempted to perform a large-scale examination of the available prospective studies to determine the association of OSA with the potential risk of vascular

For peer review only

outcomes and all-cause mortality.

Methods

Data sources, search strategy, and selection criteria

This study was conducted and reported according to the Meta-analysis of Observational Studies in Epidemiology protocol [9].

Any prospective cohort study that examined the relationship between OSA and vascular outcomes or all-cause mortality was eligible for inclusion into this study, and no restrictions were placed on language or publication status (e.g., published, in press, or in progress). Electronic databases PubMed, Embase, and the Cochrane Library were searched for articles published through May 2016, using the terms "sleep apnea" OR "obstructive sleep apneas" AND ("cardiovascular disease" OR "stroke" OR "cardiac death" OR "mortality" OR "death" OR "CVD" OR "myocardial infarction" OR "coronary events") AND "clinical trials" AND "human" as the search terms (Supplemental 1). Manual searches of reference lists were also conducted from all the relevant original and reviewed articles to identify additional eligible studies. The medical subject heading, methods, patient population, design, exposure, and outcome variables of these articles were used to identify the relevant studies.

The literature search was independently undertaken by two authors using a standardized approach. Any inconsistencies between these two authors were settled by the primary author until a consensus was reached. The study was eligible for inclusion if the following criteria were met: (1) the study had a prospective cohort

For peer review only

design; (2) the study investigated the association between OSA and the risk of major adverse cardiac events (MACEs), coronary heart disease (CHD), stroke, cardiac death, all-cause mortality, and heart failure; and (3) the authors reported effect estimates [risk ratio (RR), hazard ratio (HR), or odds ratio (OR)] and 95% confidence intervals (CIs) for comparisons of different levels of OSA versus lowest OSA level. All case-control studies were excluded because various confounding factors could bias the results.

Data collection and quality assessment

The data collected included the first author's name, publication year, country, sample size, mean age at baseline, percentage of male patients, body mass index (BMI), disease status, assessment of OSA, follow-up duration, effect estimate and its 95% CI, reported endpoints, and covariates in the fully adjusted model. For studies that reported several multivariable adjusted RRs, the effect estimate that was maximally adjusted for potential confounders was selected.

The Newcastle–Ottawa Scale (NOS), which is quite comprehensive and has been partially validated for evaluating the quality of observational studies in the meta-analysis, was used to evaluate the methodological quality [10]. The NOS is based on the following three subscales: selection (4 items), comparability (1 item), and outcome (3 items). A "star system" (range, 0–9) was developed for assessment (Table 1). The data extraction and quality assessment were conducted independently by two authors. Information was examined and adjudicated independently by an

For peer review only

additional author referring to the original studies.

Statistical analysis

The relationship between OSA and the risk of vascular outcomes or all-cause mortality based on the effect estimate (OR, RR, or HR) and its 95% CI was examined in each study. HR was considered to be equivalent to RR in cohort studies. Given the low incidence of vascular outcomes and all-cause mortality, ORs could be considered as accurate estimates of RRs. A semi-parametric method was first used to evaluate the association between mild OSA [apnea–hypopnea index (AHI): 5–15], moderate OSA (AHI: 15–30), severe OSA (AHI > 30), and the risk of vascular outcomes or all-cause mortality to analyze the trend between OSA levels and vascular outcomes or all-cause mortality risk [11]. Each category of AHI levels was established based on its calculated median, and the control category was composed of participants with lowest AHI or normal participants in each study. Furthermore, when more than one median of AHI levels in each study was classified into one of these three categories, the fixed-effects model was used to calculate their summary RRs and 95% CIs for effect estimates of each category [12]. If the study data were not broken down by AHI, rather by ODI, each category of OSA was referred to the clinicians. The random-effects model was then used to calculate summary RRs and 95% CIs for mild, moderate, and severe OSA versus normal [13]. Finally, the relative RRs and the corresponding 95% CIs were estimated using specific RRs and 95% CIs after considering the country, mean age, gender, BMI, disease status, and duration of the follow-up period [14].

For peer review only

Heterogeneity between studies was investigated using the Q statistic, and P values <0.10 was considered as indicative of significant heterogeneity [15 16]. Subgroup analyses were conducted for mild, moderate, and severe OSA and the risk of MACEs based on the country, mean age, gender, BMI, disease status, and duration of the follow-up period. A sensitivity analysis was also performed by removing each individual study from the meta-analysis [17]. Several methods were used to check for potential publication bias. Visual inspections of funnel plots for MACEs were conducted. The Egger [18] and Begg [19] tests were also used to statistically assess publication bias for MACEs. All reported P values were two sided, and P values <0.05 were considered statistically significant for all included studies. Statistical analyses were performed using the STATA software (version 12.0; Stata Corporation, TX, USA).

Results

Literature search

The results of the study-selection process are shown in Figure 1. An initial electronic search yielded 3282 articles, of which 3236 duplicates and irrelevant studies were excluded, and 46 potentially eligible studies were selected. After detailed evaluations, 16 prospective studies were selected for the final meta-analysis [20-35]. No new studies qualified for inclusion after a manual search of the reference lists of these studies. The general characteristics of the included studies are presented in Table 1.

For peer review only

Study characteristics

A total of 16 studies with 24,308 individuals qualified for this study. The follow-up period for participants was 2.9–18.0 years, while 73–10,149 individuals were included in each study. Eight studies were conducted in the United States, four in Spain, one in Sweden, one in Portugal, one in Hungary, and one in Canada. Furthermore, 11 studies reported healthy participants, and the remaining 5 studies reported participants with different diseases. The mean BMI ranged from 26.8 to 34.0 kg/m^2. Fourteen studies used polysomnography (PSG), and the remaining one study used limited PSG to assess the levels of OSA. The study quality was assessed using the NOS (Table 1). Overall, one study had a score of 9, six studies had a score of 8, seven studies had a score of 7, and the remaining two studies had a score of 6.

For peer review only

Table 1. Baseline characteristic of studies included in the systematic review and meta-analysis

Study	Country	Sample size	Mean age	Percentage male (%)	BMI	Disease status	Assessment OSA	Follow-up duration (year)	Reported outcomes	Adjusted factors	NOS score
Mooe et al. 2000 [20]	Sweden	408	59.1	58.4	27.0	CAD	Limited PSG	5.1	CHD, stroke, all-cause mortality	Age, sex, BMI, hypertension, DM, LVF, and coronary intervention	7
Gottlieb et al. 2010 [21]	USA	4422	62.4	43.5	28.2	Healthy	PSG	8.7	HF	Age, race, BMI, smoking, DM, SBP, DBP, TC, HDL-C, lipid-lowering medications, and antihypertensive medications	8

For peer review only

Campos-Rodriguez et al. 2012 [22]	Spain	1116	56.1	0.0	36.6	Healthy	PSG	6.0	Cardiac death	Age, BMI, DM, hypertension, and previous CVD	8
Marin et al. 2005 [23]	Spain	1729	49.9	100	28.7	Healthy	PSG	10.1	Cardiac death and CHD	Age, diagnostic group, presence of CVD, DM, hypertension, lipid disorders, smoking, alcohol, SBP DBP, blood glucose, TC, TG, and use of antihypertensive, lipid-lowering and antidiabetic drugs	9
Young et	USA	1522	48.0	55.0	28.6	Healthy	PSG	18.0	Cardiac death,	Age, age-squared, sex, BMI, and	8

For peer review only

al. 2008 [24]									all-cause mortality, and CHD	BMI squared	
Redline et al. 2010 [25]	USA	5422	62.9	45.4	27.8	Healthy	PSG	8.7	Stroke	Age, BMI, race, smoking, SBP, DM, and antihypertensive medications	8
Arzt et al. 2005 [26]	USA	1189	47.0	55.0	30.0	Healthy	PSG	4.0	Stroke	Age, sex, and BMI	7
Punjabi et al. 2008 [27]	USA	6294	62.5	47.0	27.8	Healthy	PSG	8.2	CHD, all-cause mortality	Age, sex, race, BMI, SBP, DBP, smoking, prevalent hypertension, DM, and CVD	8

For peer review only

Shah et al. 2010 [28]	USA	1436	59.7	69.4	32.9	Healthy	PSG	2.9	CHD, cardiac death	Age, race, sex, smoking, alcohol, BMI, AF, DM, hypertension, and hyperlipidemia	7
Yaggi et al. 2005 [29]	USA	1022	60.2	71.3	32.8	Healthy	PSG	3.4	Stroke and all-cause mortality	Age, sex, race, smoking, alcohol, BMI, DM, hyperlipidemia, AF, and hypertension	8
Martı´nez-Garcı´a et al. 2009 [30]	Spain	166	73.3	59.0	28.1	Ischemic Stroke	PSG	5.0	All-cause mortality	Age, sex, Barthel index, AHI, and CPAP treatment groups, previous stroke or TIA, diabetes, hypercholesterolemia, BMI, smoking, arterial hypertension, atrial fibrillation, significant	7

										carotid stenosis, and fibrinogen levels	
Munoz et al. 2006 [31]	Spain	1034	79.8	57.0	26.8	Healthy	PSG	6.0	Stroke	Sex	7
Leão et al. 2016 [32]	Portugal	73	62.4	75.0	27.6	Acute coronary syndrome	PSG	6.3	CHD	Sex	7
Fornadi et al. 2014 [33]	Hungary	100	51.0	56.8	26.8	Kidney transplant recipients	PSG	6.3	All-cause mortality	Unadjusted	6

For peer review only

Kendzerska et al. 2014 [34]	Canada	10149	49.9	62.0	30.1	Healthy	PSG	5.7	All-cause mortality	Traditional CV risk factors	7
Won et al. 2013 [35]	USA	281	65.0	98.0	34.0	Ischemic heart disease and myocardial injury	PSG	4.1	All-cause mortality	NA	6

AF, atrial fibrillation; BMI, body mass index; CAD, coronary artery disease; CHD, coronary heart disease; CPAP, continuous positive airway pressure; CV, cardiovascular; DBP, diastolic blood pressure; DM, diabetes mellitus; HDL-C, high-density lipoprotein cholesterol; HF, heart failure; LVF, left ventricular function; OSA, obstructive sleep apnea; PSG, polysomnography; SBP, systolic blood pressure; TC, total cholesterol; TG, triglyceride; TIA, transient ischemic attack.

For peer review only

OSA and MACE risk

The summary RRs showed that mild OSA was not associated with MACEs (RR: 0.98; 95% CI: 0.87–1.11; P = 0.741; Fig. 2 and Table 2). Furthermore, the pooled analysis results for moderate and severe OSA indicated that they had a harmful effect on the risk of MACEs (moderate: RR, 1.16; 95% CI, 1.01–1.33; P = 0.034; Fig. 3 and Table 2; severe: RR, 2.04; 95% CI, 1.56–2.66; P < 0.001; Fig. 4 and Table 2). A subgroup analysis for MACEs was conducted to minimize heterogeneity among the included studies and evaluate the relationship between OSA and MACEs in specific subpopulations (Table 3). Overall, participants with moderate OSA were associated with an increased risk of MACEs if individuals did not have other diseases (RR: 1.16; 95% CI: 1.01–1.33; P = 0.034). Furthermore, no significant association was found between severe OSA and MACEs if the study included only women (RR: 1.98; 95% CI: 0.64–6.06; P = 0.234); in other subsets, severe OSA was associated with an increased risk of MACEs (Table 3). Finally, no evidence of a factor-specific difference was found in the RR for MACEs among participants with OSA compared with controls (Table 3).

Table 2. Summary of the relative risks of all outcomes evaluated

Outcomes	Mild OSA	*P* value	Moderate OSA	*P* value	Severe OSA	*P* value
MACEs	0.98 (0.87–1.11)	0.741	1.16 (1.01–1.33)	0.034	2.04 (1.56–2.66)	<0.001
CHD	1.25 (0.95–1.66)	0.117	1.38 (1.04–1.83)	0.026	1.63 (1.18–2.26)	0.003

For peer review only

Stroke	1.29 (0.69–2.41)	0.424	1.35 (0.82–2.23)	0.245	2.15 (1.42–3.24)	<0.001
Cardiac death	1.80 (0.68–4.76)	0.236	1.11 (0.53–2.35)	0.781	2.96 (1.45–6.01)	0.003
All-cause mortality	1.26 (0.77–2.07)	0.354	1.04 (0.60–1.79)	0.895	1.54 (1.21–1.97)	<0.001
Heart failure	1.02 (0.78–1.34)	0.868	1.07 (0.74–1.54)	0.719	1.44 (0.94–2.21)	0.097

CHD, Coronary heart disease; MACE, major cardiovascular event; OSA, obstructive sleep apnea.

Table 3. Subgroup analyses for MACEs

Variable	Subgroup	Mild OSA	*P* value	Moderate OSA	*P* value	Severe OSA	*P* value
Country	USA	1.00 (0.85–1.17)	0.977	1.14 (0.99–1.32)	0.064	**1.90 (1.35–2.67)**	**<0.001**
	Other	1.02 (0.19–5.52)	0.982	1.44 (0.83–2.50)	0.198	**2.35 (1.52–3.65)**	**<0.001**
	USA vs other	0.98 (0.18–5.32)	0.982	0.79 (0.45–1.40)	0.422	0.81 (0.46–1.41)	0.453
Mean age	≥60	0.96 (0.86–1.08)	0.540	1.13 (0.97–1.33)	0.117	**1.78 (1.23–2.57)**	**0.002**
	<60	1.40 (0.73–2.70)	0.315	1.51 (0.94–2.41)	0.086	**2.31 (1.64–3.24)**	**<0.001**
	≥60 vs <60	0.69 (0.35–1.33)	0.265	0.75 (0.46–1.23)	0.252	0.77 (0.47–1.27)	0.309
Gender	Male	0.92 (0.73–1.15)	0.455	1.10 (0.85–1.42)	0.449	**1.81 (1.14–2.89)**	**0.012**
	Female	1.97 (0.47–8.25)	0.353	1.36 (0.67–2.76)	0.399	1.98 (0.64–6.06)	0.234
	Male vs female	0.47 (0.11–1.99)	0.304	0.81 (0.38–1.72)	0.581	0.91 (0.27–3.08)	0.885

BMI	≥30	1.75 (0.88–3.49)	0.111	1.70 (0.94–3.07)	0.079	**2.72 (1.80–4.10)**	**<0.001**
	<30	0.96 (0.86–1.07)	0.449	1.14 (0.99–1.31)	0.078	**1.80 (1.36–2.38)**	**<0.001**
	≥30 vs <30	1.82 (0.91–3.66)	0.092	1.49 (0.81–2.74)	0.198	1.51 (0.92–2.49)	0.104
Disease statues	Healthy	1.00 (0.85–1.17)	0.977	**1.16 (1.01–1.33)**	**0.034**	**2.12 (1.53–2.94)**	**<0.001**
	Other	1.02 (0.19–5.52)	0.982	–	-	**1.96 (1.01–3.81)**	**0.047**
	Healthy vs Other	0.98 (0.18–5.32)	0.982	–	-	1.08 (0.52–2.27)	0.835
Follow-up duration	≥6	0.96 (0.86–1.07)	0.449	1.14 (0.99–1.31)	0.064	**2.06 (1.43–2.95)**	**<0.001**
	<6	1.75 (0.88–3.49)	0.111	1.74 (0.87–3.49)	0.120	**2.10 (1.39–3.17)**	**<0.001**
	≥6 vs <6	0.55 (0.27–1.10)	0.092	0.66 (0.32–1.33)	0.242	0.98 (0.57–1.70)	0.945

OSA, obstructive sleep apnea.

OSA and CHD risk

The pooled data of meta-analysis showed that mild OSA was not associated with the risk of CHD (RR: 1.25; 95% CI: 0.95–1.66; P = 0.117; Table 2 and Supplemental 2), whereas moderate OSA (RR: 1.38; 95% CI: 1.04–1.83; P = 0.026; Table 2 and Supplemental 2) and severe OSA (RR: 1.63; 95% CI: 1.18–2.26; P = 0.003; Table 2 and Supplemental 2) were associated with a significantly increased risk of CHD. Stratified analyses according to gender were conducted for different levels of OSA versus normal group, and it was found that patients with severe OSA had significantly

For peer review only

increased the risk of CHD in men (RR: 1.65; 95% CI: 1.06–2.57; *P* = 0.027). No other significant differences were detected (Table 4).

Table 4. Gender difference for other outcomes

Outcome	Subgroup	Mild OSA	*P* value	Moderate OSA	*P* value	Severe OSA	*P* value
CHD	Men	0.93 (0.72–1.21)	0.596	1.09 (0.80–1.48)	0.582	**1.65 (1.06–2.57)**	**0.027**
	Women	1.92 (0.43–8.64)	0.394	1.51 (0.38–5.97)	0.559	1.10 (0.12–9.87)	0.933
	Men vs women	0.48 (0.11–2.22)	0.351	0.72 (0.18–2.96)	0.651	1.50 (0.16–14.22)	0.724
Stroke	Men	1.86 (0.67–5.14)	0.232	1.86 (0.70–4.95)	0.214	**2.86 (1.10–7.41)**	**0.031**
	Women	1.34 (0.76–2.36)	0.311	1.20 (0.67–2.15)	0.542	1.21 (0.65–2.25)	0.546
	Men vs women	1.39 (0.43–4.45)	0.581	1.55 (0.50–4.84)	0.451	2.36 (0.76–7.38)	0.138
Cardiac death	Men	–	–	1.15 (0.41–3.23)	0.791	**2.87 (1.13–7.27)**	**0.026**
	Women	–	–	0.94 (0.19–4.61)	0.939	3.71 (0.41–33.87)	0.245
	Men vs women	–	–	1.22 (0.18–8.17)	0.935	0.77 (0.07–8.49)	0.834
All-cause mortality	Men	–	–	–	–	**1.72 (1.22–2.43)**	**0.002**
	Women	–	–	–	–	**3.50 (1.23–9.97)**	**0.019**
	Men vs women	–	–	–	–	0.49 (0.16–1.48)	0.206
Heart	Men	0.88 (0.57–1.35)	0.561	1.13 (0.68–1.88)	0.639	1.58 (0.93–2.67)	0.088

For peer review only

failure	Women	1.13 (0.80–1.60)	0.493	1.01 (0.60–1.70)	0.970	1.19 (0.56–2.52)	0.650
	Men vs women	0.78 (0.45–1.35)	0.376	1.12 (0.54–2.32)	0.762	1.33 (0.53–3.33)	0.545

CHD, coronary heart disease; OSA, obstructive sleep apnea.

OSA and stroke risk

Pooled analysis results indicated no association between mild OSA (RR: 1.29; 95% CI: 0.69–2.41; P = 0.424; Table 2 and Supplemental 2) and moderate OSA (RR: 1.35; 95% CI: 0.82–2.23; P = 0.245; Table 2 and Supplemental 2) and stroke, whereas severe OSA was associated with an increased risk of stroke (RR: 2.15; 95% CI: 1.42–3.24; P < 0.001; Table 2 and Supplemental 2). Subgroup analysis on the basis of gender indicated that severe OSA had a harmful effect on the risk of stroke in men (RR: 2.86; 95% CI: 1.10–7.41; P = 0.031; Table 4).

OSA and cardiac death risk

The summary RRs showed that mild OSA (RR: 1.80; 95% CI: 0.68–4.76; P = 0.236; Table 2 and Supplemental 2) and moderate OSA (RR: 1.11; 95% CI: 0.53–2.35; P = 0.781; Table 2 and Supplemental 2) were not associated with cardiac death risk, whereas severe OSA significantly increased the risk of cardiac death (RR: 2.96; 95% CI: 1.45–6.01; P = 0.003; Table 2 and Supplemental 2). Subgroup analysis showed that severe OSA was associated with an increased risk of cardiac death in men (RR: 2.87; 95% CI: 1.13–7.27; P = 0.026; Table 4).

OSA and all-cause mortality risk

For peer review only

No significant association was found between mild OSA (RR: 1.26; 95% CI: 0.77–2.07; *P* = 0.354; Table 2 and Supplemental 2), moderate OSA (RR: 1.04; 95% CI: 0.60–1.79; *P* = 0.895; Table 2 and Supplemental 2), and all-cause mortality risk. However, severe OSA had a harmful impact on the all-cause mortality (RR: 1.54; 95% CI: 1.21–1.97; *P* < 0.001; Table 2 and Supplemental 2). Stratified analysis suggested that severe OSA increased the risk of all-cause mortality in men (RR: 1.72; 95% CI: 1.22–2.43; *P* = 0.002) and women (RR: 3.50; 95% CI: 1.23–9.97; *P* = 0.019; Table 4).

OSA and heart failure risk

The summary results indicated no significant differences between mild OSA (RR: 1.02; 95% CI: 0.78–1.34; *P* = 0.868), moderate OSA (RR: 1.07; 95% CI: 0.74–1.54; *P* = 0.719), and severe OSA (RR: 1.44; 95% CI: 0.94–2.21; *P* = 0.097), and the risk of heart failure (Table 2 and Supplemental 2). Subgroup analysis reported similar results compared with the overall analysis (Table 4).

Publication bias

Review of the funnel plots could not rule out the potential publication bias for MACEs (Fig. 5). The Egger and Begg test results showed no evidence of publication bias for MACEs of mild OSA (*P* value for Egger: 0.132; *P* value for Begg: 0.221) and moderate OSA (*P* value for Egger: 0.052; *P* value for Begg: 0.452). Although the Begg test showed no evidence of publication bias for MACEs of severe OSA (*P* = 0.118), the Egger test showed potential evidence of publication bias for MACEs of

For peer review only

severe OSA (P < 0.001). The conclusion did not change after adjustment for publication bias using the trim-and-fill method [36].

Discussion

The present study was based on prospective cohort studies and explored all possible correlations between OSA and the outcomes of MACEs, CHD, stroke, cardiac death, all-cause mortality, and heart failure. This large quantitative study included 24,308 individuals from 16 prospective cohort studies with a broad range of populations. The findings from the present meta-analysis suggested that mild OSA had no significant impact on the risk of vascular outcomes and all-cause mortality, moderate OSA was associated with an increased risk of MACEs and CHD, and severe OSA had a harmful effect on the risk of MACEs, CHD, stroke, cardiac death, and all-cause mortality.

A previous meta-analysis suggested that OSA was associated with stroke, but its relationship with ischemic heart disease and cardiovascular mortality needs further research [37]. However, this study could not illustrate the impact of different levels of OSA on the risk of serious cardiovascular outcomes. Further, Dong et al. suggested that moderate-to-severe OSA significantly increased the risk of cardiovascular diseases, in particular, the risk of stroke [38]. Similarly, Ge et al. indicated that severe OSA is a strong independent predictor of cardiovascular and all-cause mortality. CPAP treatment was associated with decreased cardiovascular mortality [39]. However, these two studies could not evaluate the association of OSA with the risk of vascular outcomes and all-cause mortality in specific subpopulations. In addition,

For peer review only

Wang et al. suggested that severe OSA significantly increased the risk of CHD and stroke, and all-cause mortality. A positive association with CHD was observed for moderate OSA but not for mild OSA [40]. However, whether this relationship differs according to the characteristics of participants remains unclear. Finally, Xie et al. conducted a meta-analysis to evaluate the relationship between OSA and recurrent vascular events and all-cause mortality [41]. However, they just compared the highest AHI versus lowest AHI, whereas the degree of OSA and subsequent adverse outcomes were not available. Therefore, a comprehensive meta-analysis of these prospective cohort studies was performed to evaluate any possible correlates between OSA and vascular outcomes.

No significant difference was observed between mild OSA and the risk of vascular outcomes. However, several studies included in this study reported inconsistent results. Young et al. suggested that mild OSA significantly increased the risk of CHD by 92% [24], whereas Punjabi et al. indicated that mild OSA might have a harmful effect on the risk of CHD [27]. This might be because these two studies used healthy individuals as controls, which may make them more susceptible to acquired significant conclusion. Furthermore, most of these studies did not take into account potential confounders for the risk of cardiovascular disease. Moderate-to-severe OSA might play an important role in the risk of vascular outcomes. Shah et al. concluded that OSA increased the risk of coronary events or death from cardiovascular causes [28]. Nearly all included studies reported adverse outcomes for severe OSA. Previous studies indicated that OSA was a cause of diabetes, which was an independent risk

For peer review only

factor for MACEs. Multiple adjusted models might be biased as the adjusted variables are different, reflecting either mediation or confounding.

Subgroup analyses reported similar conclusions. Gender might have an impact on the relationship between OSA and CHD, stroke, or cardiac death, although the sex difference was not statistically significant. The possible reasons could be the lower prevalence of severe OSA in women and the later age of onset of OSA in women than in men. Furthermore, OSA in women always occurred after menopause. Physiological response to OSA is another reason for this nonsignificant difference. Finally, these conclusions might be unreliable because smaller cohorts were included in each subset. Therefore, further large-scale studies were needed to verify this difference. Therefore, a relative result was given, and a synthetic and comprehensive review was provided.

No significant difference was found between mild or moderate OSA and all-cause mortality, while severe OSA was associated with an increased risk of all-cause mortality. Further, these significant associations were also observed in men and women separately. Although the effect estimate in women was larger than that in men, no gender difference was found in the relationship between OSA and all-cause mortality. This might be because the number of studies that reported the relationship between severe OSA and all-cause mortality was smaller than expected, and a broad 95% CI was acquired. Therefore, the association of severe OSA with all-cause mortality in women was variable and should be verified in future large-scale prospective studies.

For peer review only

Three strengths of this study should be highlighted. First, only prospective studies were included, which eliminated selection and recall bias, and could be of concern in retrospective case–control studies. Second, the large sample size allowed us to quantitatively assess the association of OSA with the risk of vascular outcomes and mortality, and thus the findings were potentially more robust than those of any individual study. Third, the summary RRs were calculated to evaluate any potential difference between subsets according to the characteristics of participants.

The limitations of this study were as follows: (1) the adjusted models were different across the included studies, and these factors might have played an important role in developing vascular outcomes; (2) in a meta-analysis of published studies, publication bias was an inevitable problem; and (3) the analysis used pooled data (individual data were not available), which restricted performing a more detailed relevant analysis and obtaining more comprehensive results.

The results of this study suggested that moderate-to-severe OSA might play an important role in the risk of vascular outcomes, especially for men. Future studies should focus on specific populations to analyze the gender difference to study the association between OSA and vascular outcomes.

Author Contributions

Chengjuan Xie carried out the studies, participated in collecting data, and drafted the manuscript. Ruolin Zhu performed the statistical analysis and participated in its design. Yanghua Tian and Kai Wang helped to draft the manuscript. All authors read

For peer review only

and approved the final manuscript.

Conflict of interests: All authors declare no conflict of interest.

Funding statement: This research was supported by the National Basic Research Program of China (nos. 973 Program 2015CB856405 and 2012CB720704) and the National Natural Science Foundation of China (nos. 31571149, 91432301, 81301176, and 81171273).

Data sharing statement: No additional data available.

For peer review only

References

1 Young T, Palta M, Dempsey J, et al. The occurrence of sleep-disordered breathing among middle-aged adults. *N Engl J Med* 1993;**328**(17):1230-5 doi: 10.1056/NEJM199304293281704published Online First: Epub Date]|.

2 Young T, Peppard PE, Taheri S. Excess weight and sleep-disordered breathing. *J Appl Physiol (1985)* 2005;**99**(4):1592-9 doi: 10.1152/japplphysiol.00587.2005published Online First: Epub Date]|.

3 Liu S, Lin Y, Liu X. Meta-Analysis of Association of Obstructive Sleep Apnea With Glaucoma. *J Glaucoma* 2016;**25**(1):1-7 doi: 10.1097/IJG.0000000000000357published Online First: Epub Date]|.

4 Leong WB, Jadhakhan F, Taheri S, et al. The Association between Obstructive Sleep Apnea on Diabetic Kidney Disease: A Systematic Review and Meta-Analysis. *Sleep* 2016;**39**(2):301-8 doi: 10.5665/sleep.5432published Online First: Epub Date]|.

5 Xu S, Wan Y, Xu M, et al. The association between obstructive sleep apnea and metabolic syndrome: a systematic review and meta-analysis. *BMC Pulm Med* 2015;**15**:105 doi: 10.1186/s12890-015-0102-3published Online First: Epub Date]|.

6 Kim Y, Koo YS, Lee HY, et al. Can Continuous Positive Airway Pressure Reduce the Risk of Stroke in Obstructive Sleep Apnea Patients? A Systematic Review and Meta-Analysis. *PLoS One* 2016;**11**(1):e0146317 doi: 10.1371/journal.pone.0146317published Online First: Epub Date]|.

For peer review only

7 Bratton DJ, Gaisl T, Wons AM, et al. CPAP vs Mandibular Advancement Devices and Blood Pressure in Patients With Obstructive Sleep Apnea: A Systematic Review and Meta-analysis. *JAMA* 2015;**314**(21):2280-93 doi: 10.1001/jama.2015.16303published Online First: Epub Date]|.

8 Nadeem R, Singh M, Nida M, et al. Effect of CPAP treatment for obstructive sleep apnea hypopnea syndrome on lipid profile: a meta-regression analysis. *J Clin Sleep Med* 2014;**10**(12):1295-302 doi: 10.5664/jcsm.4282published Online First: Epub Date]|.

9 Stroup DF, Berlin JA, Morton SC, et al. Meta-analysis of observational studies in epidemiology: a proposal for reporting. Meta-analysis Of Observational Studies in Epidemiology (MOOSE) group. *JAMA* 2000;**283**(15):2008-12

10 Wells G, Shea B, O'Connell D. The Newcastle-Ottawa Scale (NOS) for assessing the quality of nonrandomised studies in meta-analyses. *Ottawa Hospital Research Institute* 2009

11 Bradley TD, Floras JS. Obstructive sleep apnoea and its cardiovascular consequences. *Lancet* 2009;**373**(9657):82-93 doi: 10.1016/S0140-6736(08)61622-0published Online First: Epub Date]|.

12 DerSimonian R, Laird N. Meta-analysis in clinical trials. *Control Clin Trials* 1986;**7**(3):177-88

13 Ades AE, Lu G, Higgins JP. The interpretation of random-effects meta-analysis in decision models. *Med Decis Making* 2005;**25**(6):646-54 doi: 10.1177/0272989X05282643published Online First: Epub Date]|.

For peer review only

14 Huxley RR, Woodward M. Cigarette smoking as a risk factor for coronary heart disease in women compared with men: a systematic review and meta-analysis of prospective cohort studies. *Lancet* 2011;**378**(9799):1297-305 doi: 10.1016/S0140-6736(11)60781-2published Online First: Epub Date]|.

15 Deeks JJ, Higgins JP, Altman DG. *Analysing Data and Undertaking Meta‐Analyses*: John Wiley & Sons, Ltd, 2008.

16 Higgins JP, Thompson SG, Deeks JJ, et al. Measuring inconsistency in meta-analyses. *BMJ* 2003;**327**(7414):557-60 doi: 10.1136/bmj.327.7414.557published Online First: Epub Date]|.

17 Altman DG, Bland JM. Interaction revisited: the difference between two estimates. *BMJ* 2003;**326**(7382):219

18 Egger M, Davey Smith G, Schneider M, et al. Bias in meta-analysis detected by a simple, graphical test. *BMJ* 1997;**315**(7109):629-34

19 Begg CB, Mazumdar M. Operating characteristics of a rank correlation test for publication bias. *Biometrics* 1994;**50**(4):1088-101

20 Mooe T, Franklin KA, Wiklund U, et al. Sleep-disordered breathing and myocardial ischemia in patients with coronary artery disease. *Chest* 2000;**117**(6):1597-602

21 Gottlieb DJ, Yenokyan G, Newman AB, et al. Prospective study of obstructive sleep apnea and incident coronary heart disease and heart failure: the sleep heart health study. *Circulation* 2010;**122**(4):352-60 doi: 10.1161/CIRCULATIONAHA.109.901801published Online First: Epub

For peer review only

Date]|.

22 Campos-Rodriguez F, Martinez-Garcia MA, de la Cruz-Moron I, et al. Cardiovascular mortality in women with obstructive sleep apnea with or without continuous positive airway pressure treatment: a cohort study. *Ann Intern Med* 2012;**156**(2):115-22 doi: 10.7326/0003-4819-156-2-201201170-00006published Online First: Epub Date]|.

23 Marin JM, Carrizo SJ, Vicente E, et al. Long-term cardiovascular outcomes in men with obstructive sleep apnoea-hypopnoea with or without treatment with continuous positive airway pressure: an observational study. *Lancet* 2005;**365**(9464):1046-53 doi: 10.1016/S0140-6736(05)71141-7published Online First: Epub Date]|.

24 Young T, Finn L, Peppard PE, et al. Sleep disordered breathing and mortality: eighteen-year follow-up of the Wisconsin sleep cohort. *Sleep* 2008;**31**(8):1071-8

25 Redline S, Yenokyan G, Gottlieb DJ, et al. Obstructive sleep apnea-hypopnea and incident stroke: the sleep heart health study. *Am J Respir Crit Care Med* 2010;**182**(2):269-77 doi: 10.1164/rccm.200911-1746OCpublished Online First: Epub Date]|.

26 Arzt M, Young T, Finn L, et al. Association of sleep-disordered breathing and the occurrence of stroke. *Am J Respir Crit Care Med* 2005;**172**(11):1447-51 doi: 10.1164/rccm.200505-702OCpublished Online First: Epub Date]|.

For peer review only

27 Punjabi NM, Newman AB, Young TB, et al. Sleep-disordered breathing and cardiovascular disease: an outcome-based definition of hypopneas. *Am J Respir Crit Care Med* 2008;**177**(10):1150-5 doi: 10.1164/rccm.200712-1884OCpublished Online First: Epub Date]|.

28 Shah NA, Yaggi HK, Concato J, et al. Obstructive sleep apnea as a risk factor for coronary events or cardiovascular death. *Sleep Breath* 2010;**14**(2):131-6 doi: 10.1007/s11325-009-0298-7published Online First: Epub Date]|.

29 Yaggi HK, Concato J, Kernan WN, et al. Obstructive sleep apnea as a risk factor for stroke and death. *N Engl J Med* 2005;**353**(19):2034-41 doi: 10.1056/NEJMoa043104published Online First: Epub Date]|.

30 Martinez-Garcia MA, Soler-Cataluna JJ, Ejarque-Martinez L, et al. Continuous positive airway pressure treatment reduces mortality in patients with ischemic stroke and obstructive sleep apnea: a 5-year follow-up study. *Am J Respir Crit Care Med* 2009;**180**(1):36-41 doi: 10.1164/rccm.200808-1341OCpublished Online First: Epub Date]|.

31 Munoz R, Duran-Cantolla J, Martinez-Vila E, et al. Severe sleep apnea and risk of ischemic stroke in the elderly. *Stroke* 2006;**37**(9):2317-21 doi: 10.1161/01.STR.0000236560.15735.0fpublished Online First: Epub Date]|.

32 Leao S, Conde B, Fontes P, et al. Effect of Obstructive Sleep Apnea in Acute Coronary Syndrome. *Am J Cardiol* 2016;**117**(7):1084-7 doi: 10.1016/j.amjcard.2015.12.053published Online First: Epub Date]|.

33 Fornadi K, Ronai KZ, Turanyi CZ, et al. Sleep apnea is not associated with worse

For peer review only

outcomes in kidney transplant recipients. *Sci Rep* 2014;**4**:6987 doi: 10.1038/srep06987published Online First: Epub Date]|.

34 Kendzerska T, Gershon AS, Hawker G, et al. Obstructive sleep apnea and risk of cardiovascular events and all-cause mortality: a decade-long historical cohort study. *PLoS Med* 2014;**11**(2):e1001599 doi: 10.1371/journal.pmed.1001599published Online First: Epub Date]|.

35 Won CH, Chun HJ, Chandra SM, et al. Severe obstructive sleep apnea increases mortality in patients with ischemic heart disease and myocardial injury. *Sleep Breath* 2013;**17**(1):85-91 doi: 10.1007/s11325-012-0653-ypublished Online First: Epub Date]|.

36 Duval S, Tweedie R. A Nonparametric "Trim and Fill" Method of Accounting for Publication Bias in Meta-Analysis. *Journal of the American Statistical Association* 2000;**95**(449):89-98

37 Loke YK, Brown JW, Kwok CS, et al. Association of obstructive sleep apnea with risk of serious cardiovascular events: a systematic review and meta-analysis. *Circ Cardiovasc Qual Outcomes* 2012;**5**(5):720-8 doi: 10.1161/CIRCOUTCOMES.111.964783published Online First: Epub Date]|.

38 Dong JY, Zhang YH, Qin LQ. Obstructive sleep apnea and cardiovascular risk: meta-analysis of prospective cohort studies. *Atherosclerosis* 2013;**229**(2):489-95 doi: 10.1016/j.atherosclerosis.2013.04.026published Online First: Epub Date]|.

39 Ge X, Han F, Huang Y, et al. Is obstructive sleep apnea associated with

For peer review only

cardiovascular and all-cause mortality? *PLoS One* 2013;**8**(7):e69432 doi: 10.1371/journal.pone.0069432published Online First: Epub Date]|.

40 Wang X, Ouyang Y, Wang Z, et al. Obstructive sleep apnea and risk of cardiovascular disease and all-cause mortality: a meta-analysis of prospective cohort studies. *Int J Cardiol* 2013;**169**(3):207-14 doi: 10.1016/j.ijcard.2013.08.088published Online First: Epub Date]|.

41 Xie W, Zheng F, Song X. Obstructive sleep apnea and serious adverse outcomes in patients with cardiovascular or cerebrovascular disease: a PRISMA-compliant systematic review and meta-analysis. *Medicine (Baltimore)* 2014;**93**(29):e336. doi: 10.1097/MD.0000000000000336.

For peer review only

Figure legends:

Figure 1. Study-selection process.

Figure 2. Association between mild OSA and MACEs.

Figure 3. Association between moderate OSA and MACEs.

Figure 4. Association between severe OSA and MACEs.

Figure 5. Funnel plots.

For peer review only

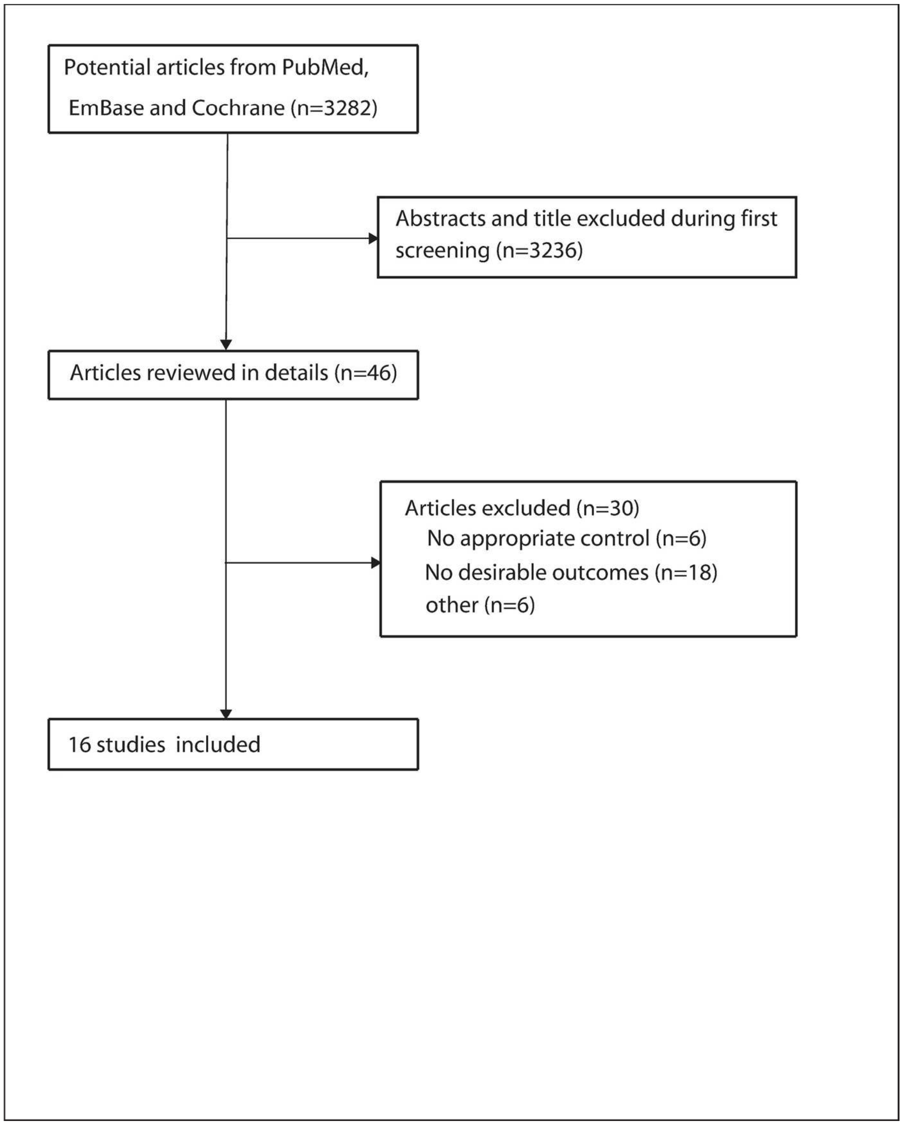

Figure 1

168x188mm (300 x 300 DPI)

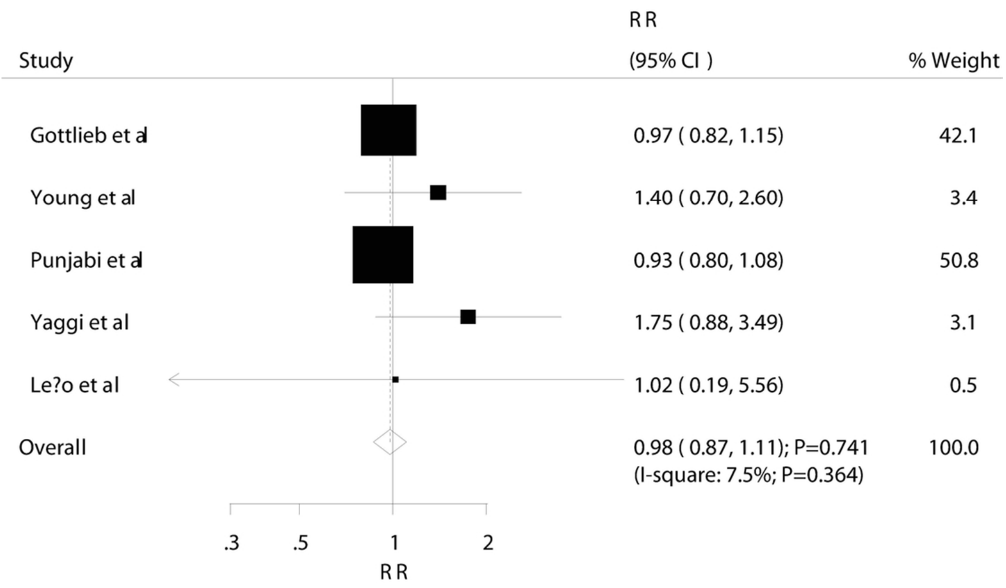

Figure 2

94x54mm (300 x 300 DPI)

review only

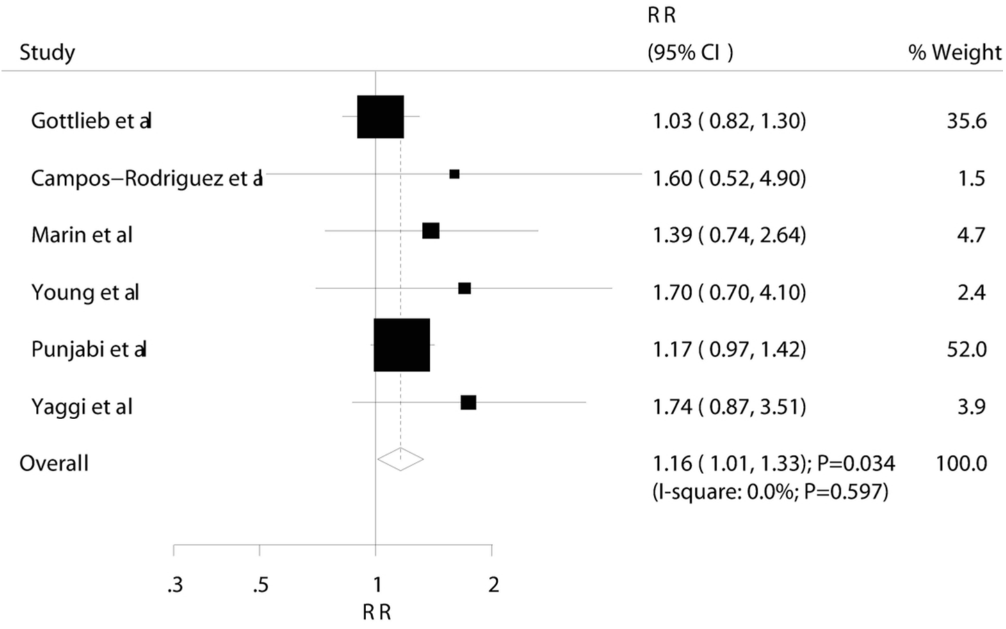

Figure 3

98x61mm (300 x 300 DPI)

For peer review only - http://bmjopen.bmj.com/site/about/guidelines.xhtml

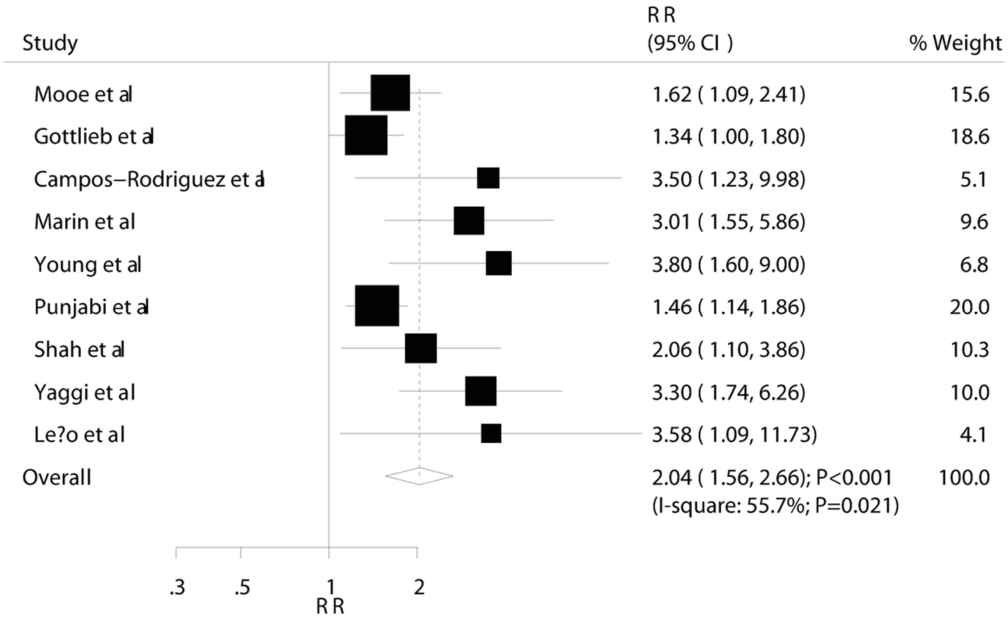

Figure 4

98x60mm (300 x 300 DPI)

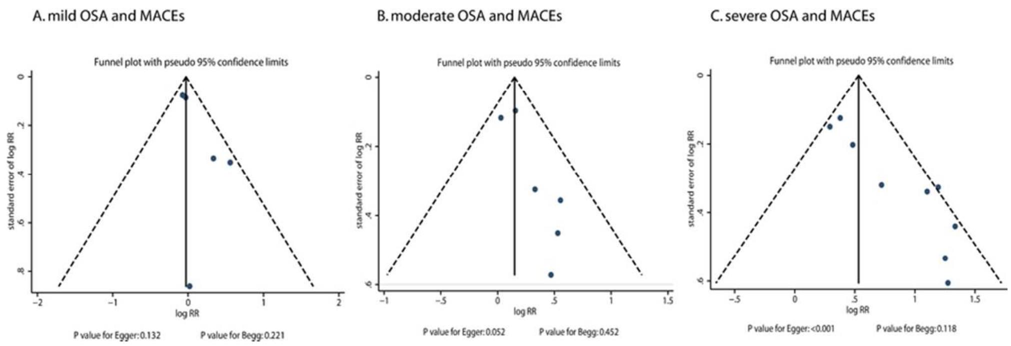

Figure 5

75x25mm (300 x 300 DPI)

peer review only

Title:

	**Search strategy**
**#1**	"Sleep Apnea, Obstructive" [Mesh] OR "OSA" [All fields] OR "OHS" [All fields]
**#2**	Apneas, Obstructive Sleep OR Obstructive Sleep Apneas OR Sleep Apneas, Obstructive OR Obstructive Sleep Apnea Syndrome OR Obstructive Sleep Apnea OR OSAHS OR Syndrome, Sleep Apnea, Obstructive OR Sleep Apnea Syndrome, Obstructive OR Apnea, Obstructive Sleep OR Sleep Apnea Hypopnea Syndrome OR Syndrome, Obstructive Sleep Apnea OR Upper Airway Resistance Sleep Apnea Syndrome OR Syndrome, Upper Airway Resistance, Sleep Apnea OR Hypoventilation Syndrome, Obesity OR Syndrome, Obesity Hypoventilation OR Pickwickian Syndrome OR Syndrome, Pickwickian OR Obesity-Hypoventilation Syndrome
**#3**	"Sleep Apnea Syndromes" [Mesh] OR "SAS" [All fields]
**#4**	Apnea Syndrome, Sleep OR Apnea Syndromes, Sleep OR Sleep Apnea Syndrome OR Apnea, Sleep OR Apneas, Sleep OR Sleep Apnea OR Sleep Apneas OR Sleep Hypopnea OR Hypopnea, Sleep OR Hypopneas, Sleep OR Sleep Hypopneas OR Sleep-Disordered Breathing OR Breathing, Sleep-Disordered OR Sleep Disordered Breathing OR Sleep Apnea, Mixed Central and Obstructive OR Mixed Central and Obstructive Sleep Apnea OR Sleep Apnea, Mixed OR Mixed Sleep Apnea OR Mixed Sleep Apneas OR Sleep Apneas, Mixed OR Hypersomnia with Periodic Respiration
**#5**	"Sleep Apnea, Central" [Mesh] OR "CSA"[All fields]
**#6**	Apneas, Central Sleep OR Central Sleep Apneas OR Sleep Apneas, Central OR Apnea, Central OR Apneas, Central OR Central Apnea OR Central Apneas OR Apnea, Central Sleep OR Apnea, Sleep, Central OR Sleep Apnea, Lethal Central OR Central Sleep Apnea OR Central Sleep Apnea Syndrome OR Central Sleep Disordered Breathing OR Hypoventilation, Central Alveolar OR Alveolar Hypoventilation, Central OR Alveolar Hypoventilations, Central OR Central Alveolar Hypoventilation OR Hypoventilations, Central Alveolar OR Ondine Syndrome OR Sleep-Disordered Breathing, Central OR Breathing, Central Sleep-Disordered OR Breathings, Central Sleep-Disordered OR Central Sleep-Disordered Breathing OR Central Sleep-Disordered Breathings OR Sleep Disordered Breathing, Central OR Sleep-Disordered Breathings, Central OR Central Alveolar Hypoventilation Syndrome OR Central Sleep Apnea, Secondary OR Secondary Central Sleep Apnea OR Sleep Apnea, Newborn, Primary OR Primary Sleep Apneas of Newborn OR Newborn Primary Sleep Apneas OR Central Sleep Apnea, Primary OR Primary Central Sleep Apnea
**#7**	"Continuous Positive Airway Pressure" [Mesh] OR "CPAP" [All fields] OR "Continuous Positive Airway Pressure/therapy" [Mesh]
**#8**	CPAP Ventilation OR Ventilation, CPAP OR Biphasic Continuous Positive Airway Pressure OR Bilevel Continuous Positive Airway Pressure OR Nasal Continuous Positive Airway Pressure OR nCPAP Ventilation OR Ventilation, nCPAP OR Airway Pressure Release Ventilation OR APRV Ventilation Mode OR APRV Ventilation Modes OR Ventilation Mode, APRV OR Ventilation Modes, APRV

For peer review only

**#9**	#1 OR #2 OR #3 OR #4 OR #5 OR #6 OR #7 OR #8
#10	"Cardiovascular System" [Mesh]
#11	"Death" [Mesh] OR Determination of Death OR Near-Death Experience OR Cardiac Death OR Death, Cardiac OR "Mortality" [Mesh] OR "mortality" [All fields] OR "Disease" [Mesh] OR "disease*"
**#12**	#10 AND #11
**#13**	"Cardiovascular Diseases" [Mesh] OR "CVD" OR Cardiovascular Disease OR Disease, Cardiovascular OR Diseases, Cardiovascular
**#14**	"Myocardial Infarction" [Mesh] OR "MI" OR Infarction, Myocardial OR Infarctions, Myocardial OR Myocardial Infarctions OR Cardiovascular Stroke OR Cardiovascular Strokes OR Stroke, Cardiovascular OR Strokes, Cardiovascular OR Heart Attack OR Heart Attacks OR Myocardial Infarct OR Infarct, Myocardial OR Infarcts, Myocardial OR Myocardial Infarcts
**#15**	"Angina Pectoris" [Mesh] OR "Angina, Stable" [Mesh] OR "Microvascular Angina" [Mesh] OR "Angina, Unstable" [Mesh] OR Stenocardia OR Stenocardias OR Angor Pectoris OR "angina" [All fields] OR "Coronary Artery Disease" [Mesh] OR "CAD" OR "ischemic heart disease" [All fields] OR "Heart Failure" [Mesh] OR "Heart Failure, Diastolic" [Mesh] OR "Heart Failure, Systolic" [Mesh]
**#16**	"Cerebrovascular Disorders" [Mesh] OR "cerebrovascular" [All fields] OR "stroke*"
**#17**	"Death" [Mesh] OR Determination of Death OR Near-Death Experience OR Cardiac Death OR Death, Cardiac OR "Mortality" [Mesh] OR "mortality" [All fields] OR "mortality*"
**#18**	#12 OR #13 OR #14 OR #15 OR #16 OR #17
**#19**	"Prospective Studies" [Mesh] OR "Cohort Studies" [Mesh] OR "Follow-Up Studies" [Mesh] OR "prospective study" OR "cohort study" OR "follow-up study"
**#20**	#9 AND #18 AND #19

For peer review only

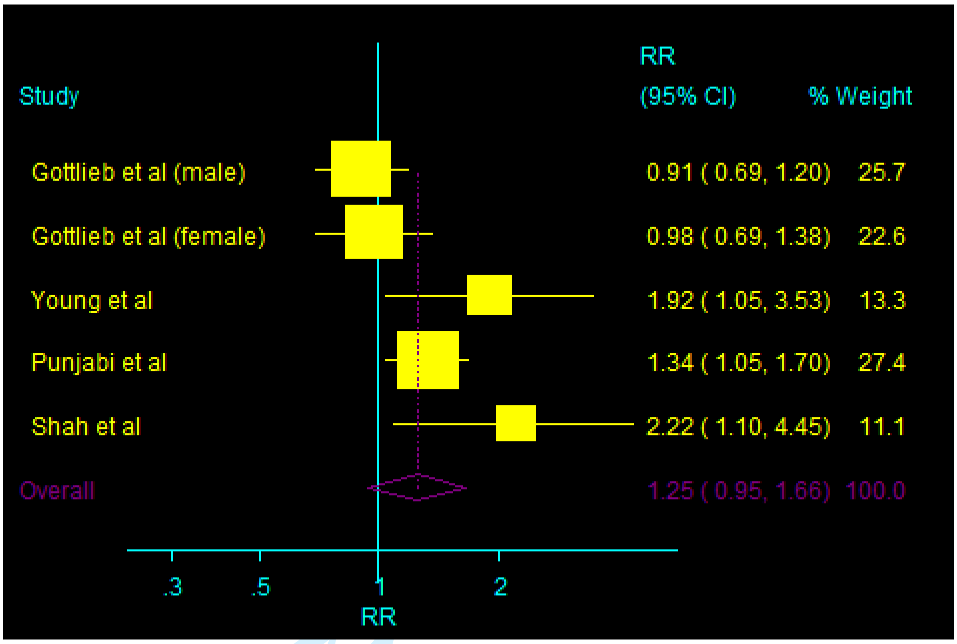

Figure S1. Association between mild OSA and CHD.

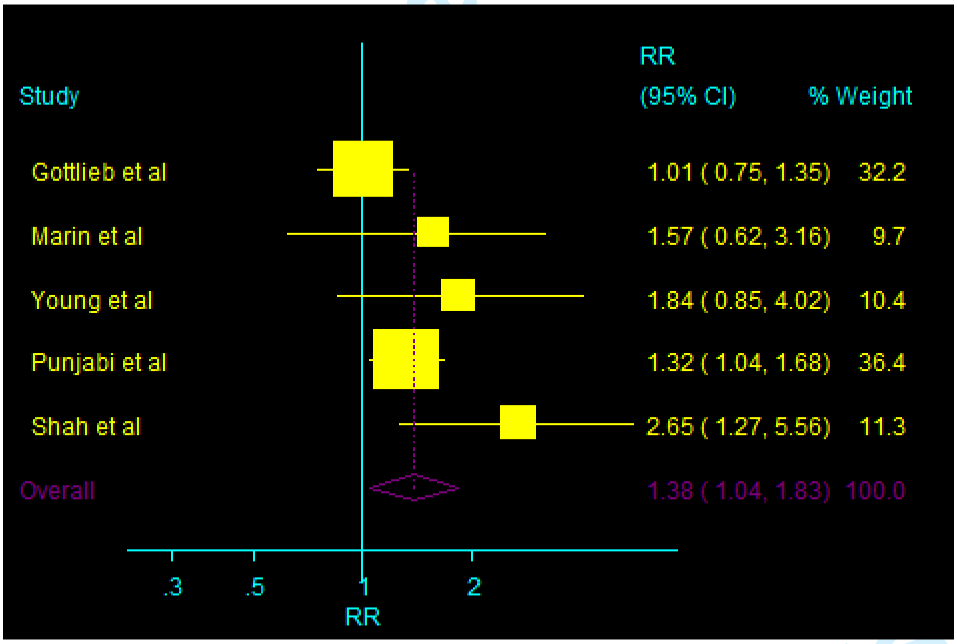

Figure S2. Association between moderate OSA and CHD.

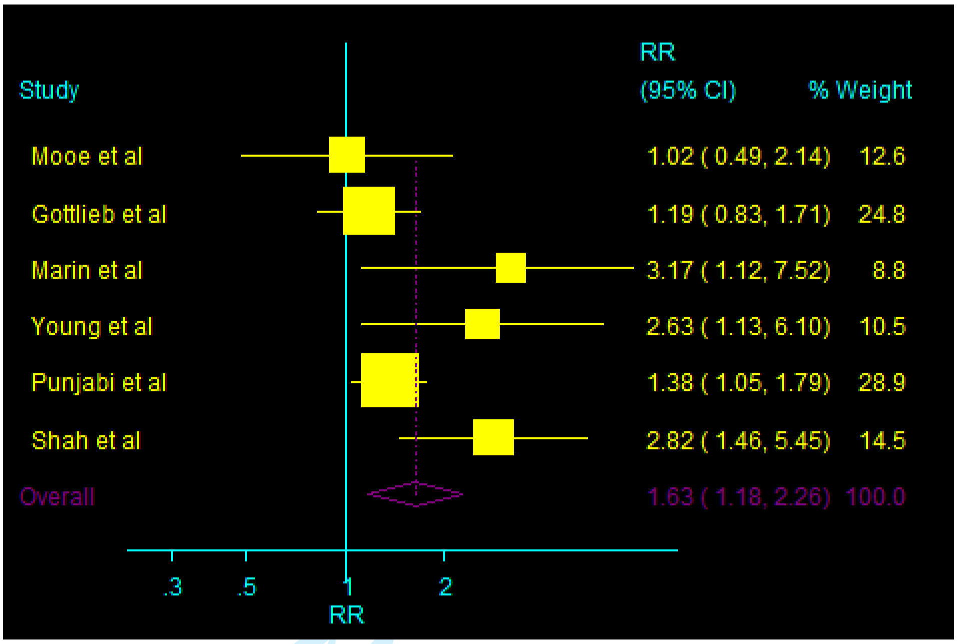

Figure S3. Association between severe OSA and CHD.

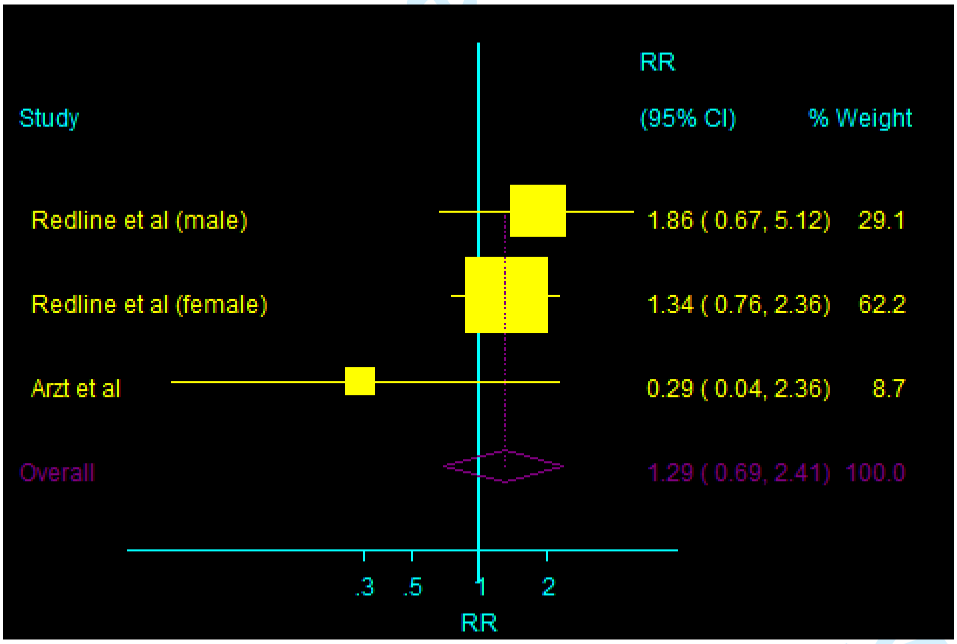

Figure S4. Association between mild OSA and stroke.

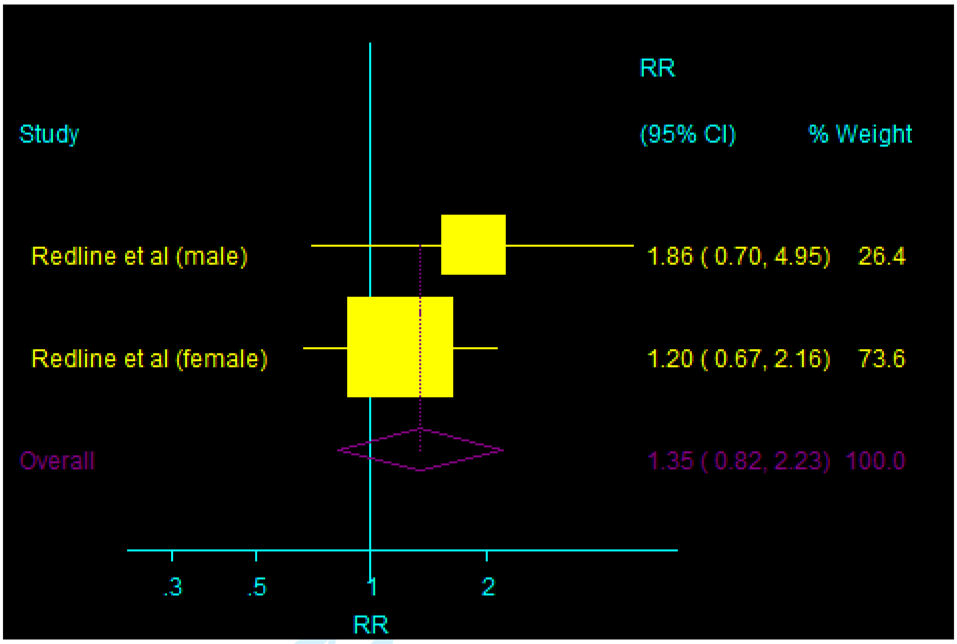

Figure S5. Association between moderate OSA and stroke.

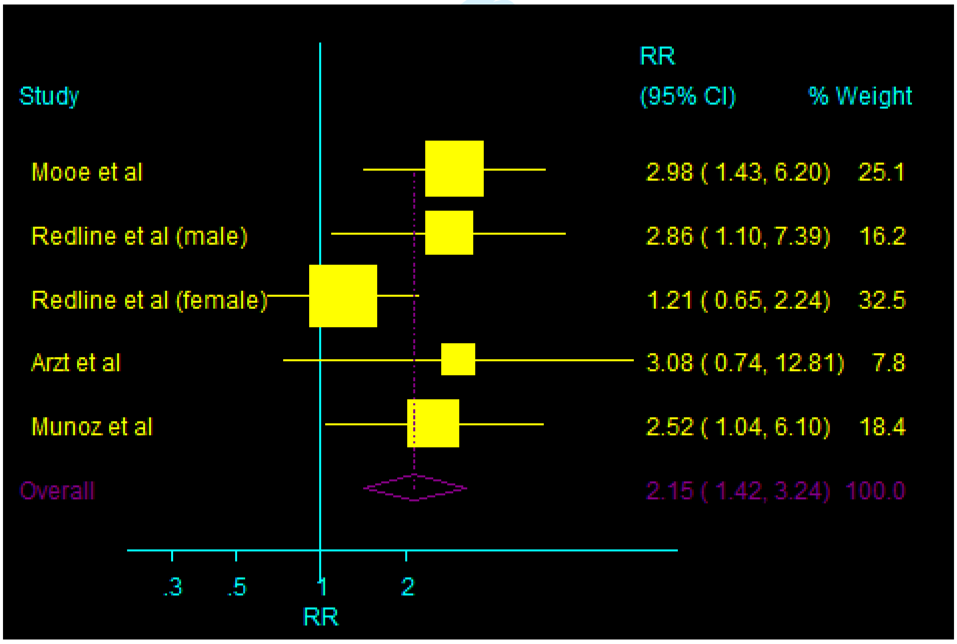

Figure S6. Association between severe OSA and stroke

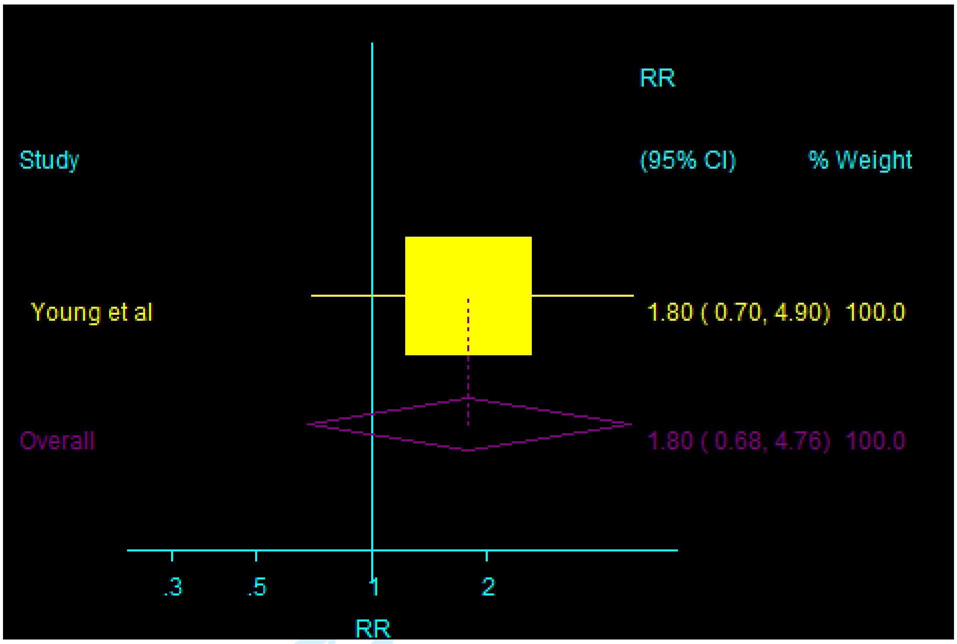

Figure S7. Association between mild OSA and cardiac death.

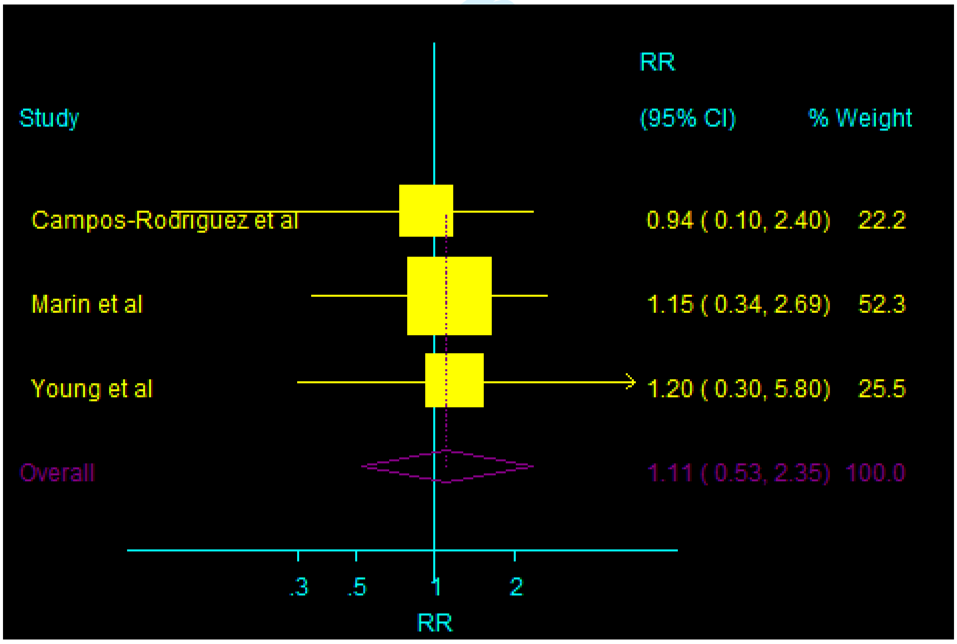

Figure S8. Association between moderate OSA and cardiac death.

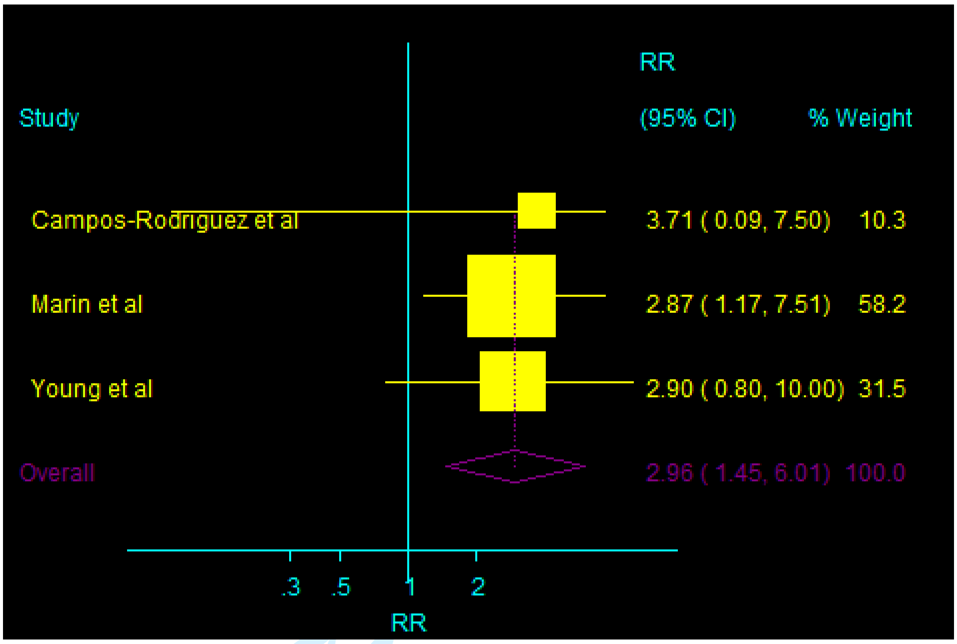

Figure S9. Association between severe OSA and cardiac death.

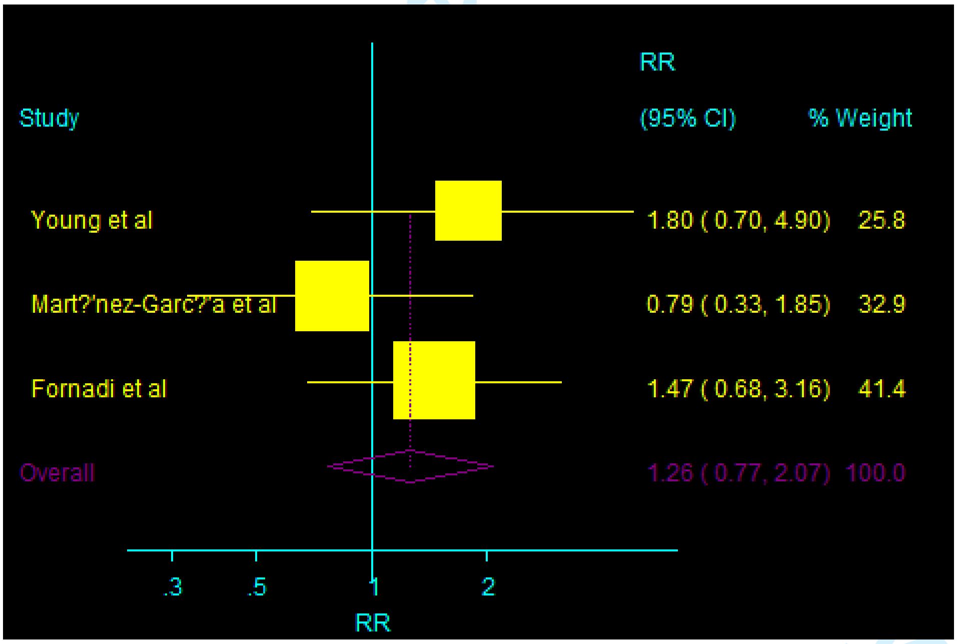

Figure S10. Association between mild OSA and all-cause death.

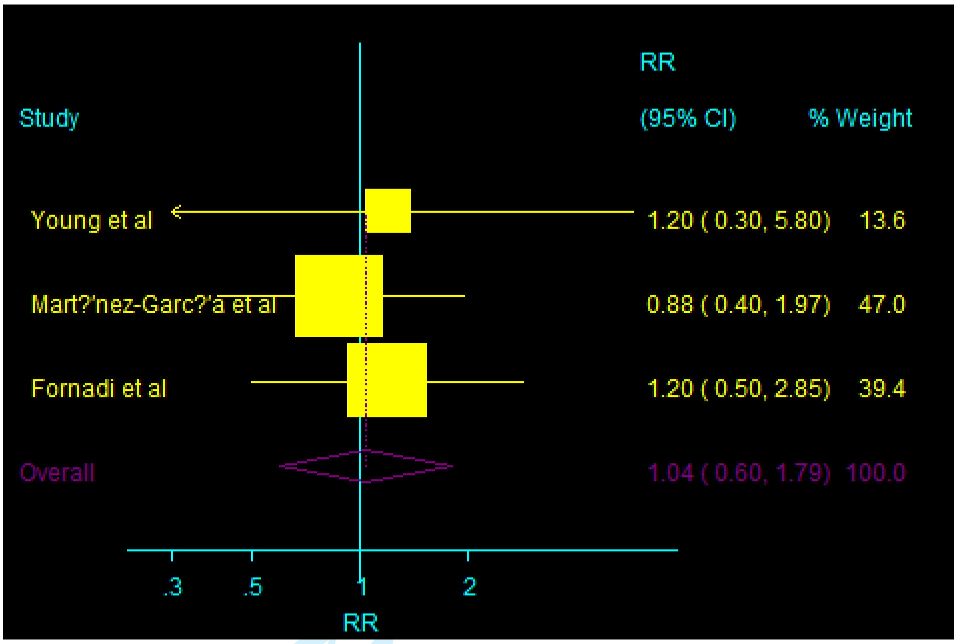

Figure S11. Association between moderate OSA and all-cause death.

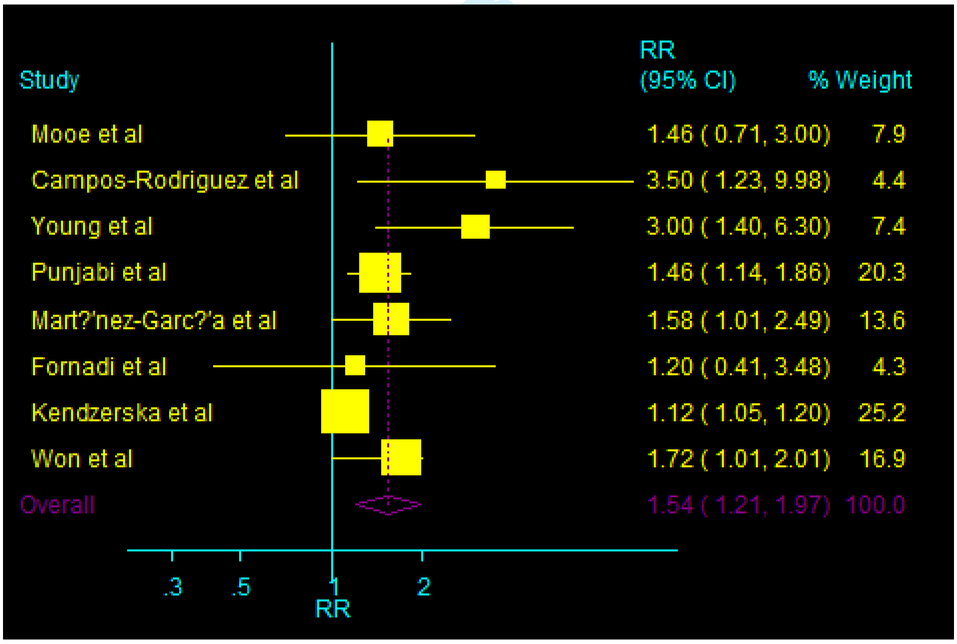

Figure S12. Association between severe OSA and all-cause death.

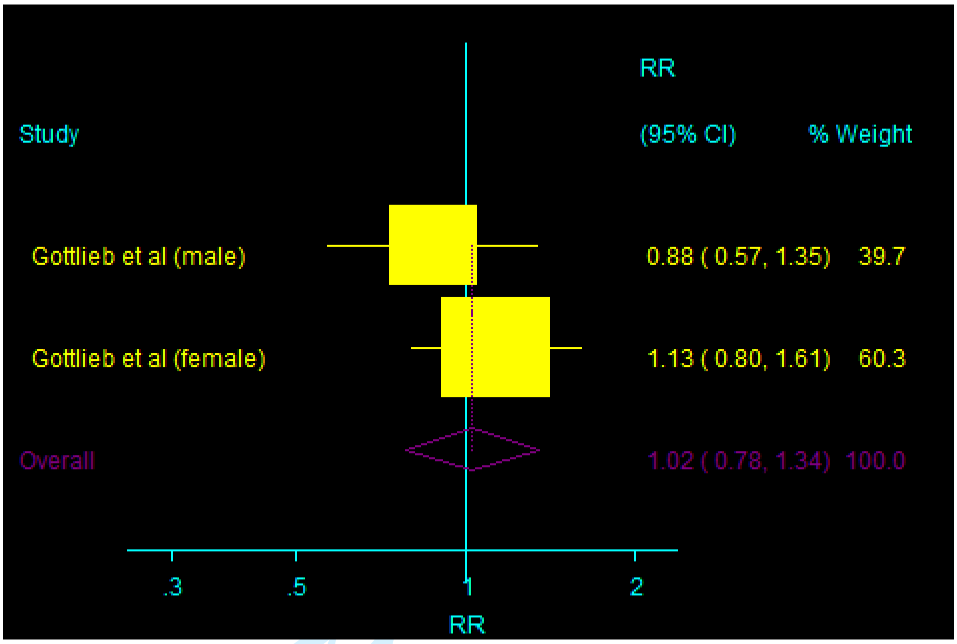

Figure S13. Association between mild OSA and heart failure.

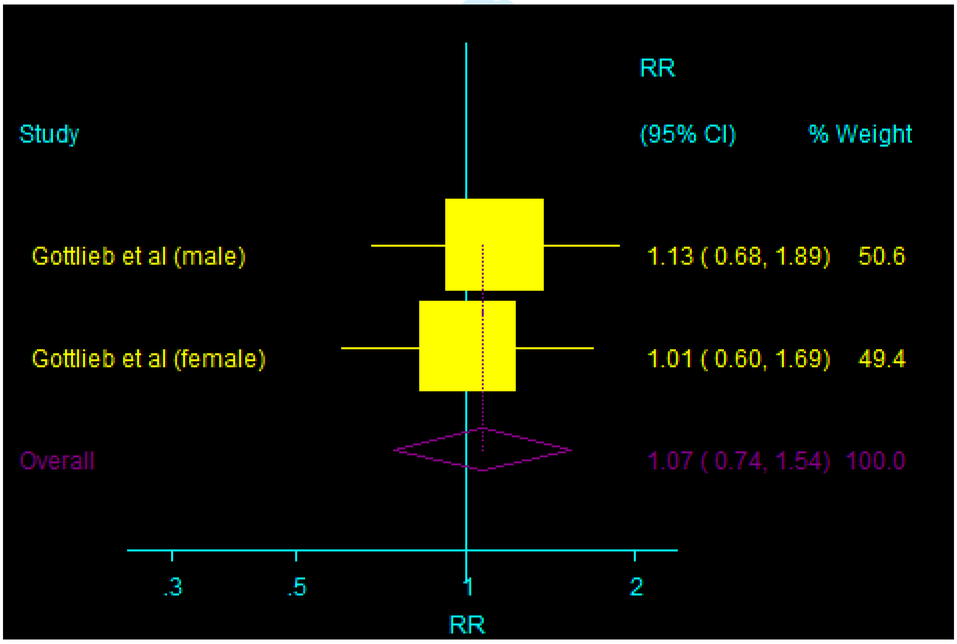

Figure S14. Association between moderate OSA and heart failure.

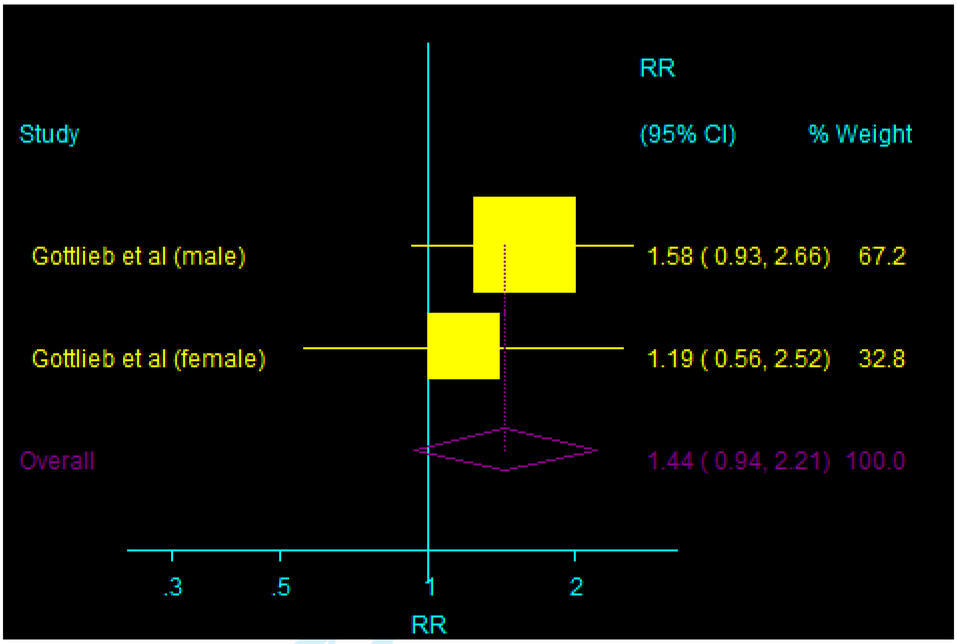

Figure S15. Association between severe OSA and heart failure.

STROBE 2007 (v4) checklist of items to be included in reports of observational studies in epidemiology*
Checklist for cohort, case-control, and cross-sectional studies (combined)

Section/Topic	Item #	Recommendation	Reported on page #
**Title and abstract**	1	(*a*) Indicate the study's design with a commonly used term in the title or the abstract	1
		(*b*) Provide in the abstract an informative and balanced summary of what was done and what was found	2
**Introduction**			3–4
Background/rationale	2	Explain the scientific background and rationale for the investigation being reported	3
Objectives	3	State specific objectives, including any pre-specified hypotheses	4
**Methods**			4–7
Study design	4	Present key elements of study design early in the paper	4
Setting	5	Describe the setting, locations, and relevant dates, including periods of recruitment, exposure, follow-up, and data collection	
Participants	6	(*a*) *Cohort study*—Give the eligibility criteria, and the sources and methods of selection of participants. Describe methods of follow-up<br>*Case-control study*—Give the eligibility criteria, and the sources and methods of case ascertainment and control selection. Give the rationale for the choice of cases and controls<br>*Cross-sectional study*—Give the eligibility criteria, and the sources and methods of selection of participants	
		(*b*) *Cohort study*—For matched studies, give matching criteria and number of exposed and unexposed<br>*Case-control study*—For matched studies, give matching criteria and the number of controls per case	
Variables	7	Clearly define all outcomes, exposures, predictors, potential confounders, and effect modifiers. Give diagnostic criteria, if applicable	
Data sources/ measurement	8*	For each variable of interest, give sources of data and details of methods of assessment (measurement). Describe comparability of assessment methods if there is more than one group	*4*
Bias	9	Describe any efforts to address potential sources of bias	
Study size	10	Explain how the study size was arrived at	
Quantitative variables	11	Explain how quantitative variables were handled in the analyses. If applicable, describe which groupings were chosen and why	5–6
Statistical methods	12	(*a*) Describe all statistical methods, including those used to control for confounding	6
		(*b*) Describe any methods used to examine subgroups and interactions	6
		(*c*) Explain how missing data were addressed	7
		(*d*) *Cohort study*—If applicable, explain how loss to follow-up was addressed<br>*Case-control study*—If applicable, explain how matching of cases and controls was addressed	7

For peer review only

		Cross-sectional study—If applicable, describe analytical methods taking account of sampling strategy	
		(*e*) Describe any sensitivity analyses	
**Results**			7-20
Participants	13*	(a) Report numbers of individuals at each stage of study—eg numbers potentially eligible, examined for eligibility, confirmed eligible, included in the study, completing follow-up, and analysed	
		(b) Give reasons for non-participation at each stage	
		(c) Consider use of a flow diagram	
Descriptive data	14*	(a) Give characteristics of study participants (eg demographic, clinical, social) and information on exposures and potential confounders	
		(b) Indicate number of participants with missing data for each variable of interest	
		(c) *Cohort study*—Summarise follow-up time (eg, average and total amount)	
Outcome data	15*	*Cohort study*—Report numbers of outcome events or summary measures over time	
		Case-control study—Report numbers in each exposure category, or summary measures of exposure	
		Cross-sectional study—Report numbers of outcome events or summary measures	
Main results	16	(*a*) Give unadjusted estimates and, if applicable, confounder-adjusted estimates and their precision (eg, 95% confidence interval). Make clear which confounders were adjusted for and why they were included	
		(*b*) Report category boundaries when continuous variables were categorized	
		(*c*) If relevant, consider translating estimates of relative risk into absolute risk for a meaningful time period	
Other analyses	17	Report other analyses done—eg analyses of subgroups and interactions, and sensitivity analyses	
**Discussion**			21-23
Key results	18	Summarise key results with reference to study objectives	22
Limitations	19	Discuss limitations of the study, taking into account sources of potential bias or imprecision. Discuss both direction and magnitude of any potential bias	
Interpretation	20	Give a cautious overall interpretation of results considering objectives, limitations, multiplicity of analyses, results from similar studies, and other relevant evidence	
Generalisability	21	Discuss the generalisability (external validity) of the study results	23
**Other information**			
Funding	22	Give the source of funding and the role of the funders for the present study and, if applicable, for the original study on which the present article is based	24

*Give information separately for cases and controls in case-control studies and, if applicable, for exposed and unexposed groups in cohort and cross-sectional studies.

Note: An Explanation and Elaboration article discusses each checklist item and gives methodological background and published examples of transparent reporting. The STROBE checklist is best used in conjunction with this article (freely available on the Web sites of PLoS Medicine at http://www.plosmedicine.org/, Annals of Internal Medicine at http://www.annals.org/, and Epidemiology at http://www.epidem.com/). Information on the STROBE Initiative is available at www.strobe-statement.org.

For peer review only

MOOSE Statement: Reporting Checklist for Authors, Editors, and Reviewers of Meta-analyses of Observational Studies

Reporting Criteria	Reported (Yes/No)	Reported on Page
**Reporting of background should include**		
Problem definition	Yes	3
Hypothesis statement	Yes	3
Description of study outcomes	Yes	3
Type of exposure or intervention used	Yes	3 – 4
Type of study designs used	Yes	4
Study population	Yes	4
**Reporting of search strategy should include**		
Qualifications of searchers (e.g., librarians and investigators)	Yes	4 – 5
Search strategy, including time period used in the synthesis and key words	Yes	5
Effort to include all available studies, including contact with authors	Yes	5
Databases and registries searched	Yes	4–5
Search software used, name and version, including special features used (e.g., explosion)	Yes	4–5
Use of hand searching (e.g., reference lists of obtained articles)	Yes	5
List of citations located and those excluded, including justification	Yes	8
Method of addressing articles published in languages other than English	Yes	4
Method of handling abstracts and unpublished studies	Yes	4–5
Description of any contact with authors	No	NA
**Reporting of methods should include**		
Description of relevance or appropriateness of studies assembled for assessing the hypothesis to be tested	No	5
Rationale for the selection and coding of data (e.g., sound clinical principles or convenience)	Yes	5–6
Documentation of how data were classified and coded (e.g., multiple raters, blinding and inter-rater reliability)	Yes	5–6
Assessment of confounding (e.g., comparability of cases and controls in studies where appropriate)	Yes	6
Assessment of study quality, including blinding of quality assessors, and stratification or regression on possible predictors of study results	Yes	6

For peer review only

Assessment of heterogeneity	Yes	7
Description of statistical methods (e.g., complete description of fixed- or random-effects models, justification of whether the chosen models account for predictors of study results, dose–response models, or cumulative meta-analysis) in sufficient detail to be replicated	Yes	6–7
Provision of appropriate tables and graphics	Yes	6–7
**Reporting of results should include**		
Graphic summarizing individual study estimates and overall estimate	Yes	8
Table giving descriptive information for each study included	Yes	8–14
Results of sensitivity testing (e.g., subgroup analysis)	Yes	15–20
Indication of statistical uncertainty of findings	Yes	20
**Reporting of discussion should include**		
Quantitative assessment of bias (e.g., publication bias)	Yes	20
Justification for exclusion (e.g., exclusion of non-English language citations)	No	21
Assessment of quality of included studies	Yes	Table 1
Strengths and weaknesses	Yes	23
**Reporting of conclusions should include**		
Consideration of alternative explanations for observed results	Yes	20–23
Generalization of the conclusions (e.g., appropriate for the data presented and within the domain of the literature review)	Yes	23
Guidelines for future research	Yes	23
Disclosure of funding source	Yes	24

NA, Not applicable.

For peer review only

BMJ Open

Association of obstructive sleep apnea with the risk of vascular outcomes and all-cause mortality: a meta-analysis

Journal:	*BMJ Open*
Manuscript ID	bmjopen-2016-013983.R2
Article Type:	Research
Date Submitted by the Author:	19-Sep-2017

Complete List of Authors:	Xie, Chengjuan; The First Affiliated Hospital of Anhui Medical University, Department of Neurology<br>Zhu, Ruolin; The First Affiliated Hospital of Anhui Medical University, Department of Neurology<br>Tian, Yanghua; The First Affiliated Hospital of Anhui Medical University, Department of Neurology<br>Wang, Kai; Department of Neurology

**Primary Subject Heading**:	Neurology
Secondary Subject Heading:	Cardiovascular medicine
Keywords:	meta-analysis, mortality, obstructive sleep apnea, vascular outcome

SCHOLARONE™
Manuscripts

For peer review only

Association of obstructive sleep apnea with the risk of vascular outcomes and all-cause mortality: a meta-analysis

Chengjuan Xie[1], Ruolin Zhu[1], Yanghua Tian[1,2], Kai Wang[1, 2, 3,*]

[1]Department of Neurology, The First Affiliated Hospital of Anhui Medical University, Hefei, China

[2]Collaborative Innovation Centre of Neuropsychiatric Disorders and Mental Health, Anhui Province, China

[3]Department of Medical Psychology, Anhui Medical University, Hefei, China

***Correspondence author:**

Kai Wang,

Department of Neurology, The First Affiliated Hospital of Anhui Medical University, Hefei, 230022,China;Collaborative Innovation Centre of Neuropsychiatric Disorders and Mental Health, Anhui Province, 230022,China;Department of Medical Psychology, Anhui Medical University, Hefei, 230022,China

Tel: +86-0551-62922418,

Fax:+86-021-64085875,

E-mail: xiecj2016@163.com.

Word count: 6288

For peer review only

Abstract

Objective: This study aimed to conduct a meta-analysis to explore and summarize the evidence regarding the association between obstructive sleep apnea (OSA) and the subsequent risk of vascular outcomes and all-cause mortality.

Methods: Electronic databases PubMed, Embase, and the Cochrane Library were searched to identify studies conducted through May 2016. Prospective cohort studies that reported effect estimates with 95% confidence intervals of major adverse cardiac events (MACEs), coronary heart disease (CHD), stroke, cardiac death, all-cause mortality, and heart failure for different levels versus the lowest level of OSA were included.

Results: A total of 16 cohort studies reporting data on 24,308 individuals were included. Of these, 11 studies reported healthy participants, and the remaining 5 studies reported participants with different diseases. Severe OSA was associated with a greater risk of MACEs ($P < 0.001$), CHD ($P = 0.003$), stroke ($P < 0.001$), cardiac death ($P = 0.003$), and all-cause mortality ($P < 0.001$); moderate OSA had a harmful impact on MACEs ($P = 0.034$) and CHD ($P = 0.026$); and no significant association was found between mild OSA and the risk of vascular outcomes or all-cause mortality ($P > 0.05$). Finally, no evidence of a factor-specific difference in the risk ratio for MACEs among participants with different levels of OSA compared with those with the lowest level of OSA was found.

Conclusions: Severe and moderate OSAs were associated with an increased risk of

For peer review only

vascular outcomes and all-cause mortality. This relationship might differ between genders. Therefore, further large-scale prospective studies are needed to verify this difference.

Key words: Meta-analysis; mortality, obstructive sleep apnea, vascular outcome

For peer review only

Article Summary:

Strengths and limitations of this study:

1. This was a meta-analysis of prospective observational studies designed to elucidate the association of obstructive sleep apnea (OSA) with fatal and nonfatal cardiovascular diseases.

2. The findings were based on a large sample size and are more robust than those obtained from any individual study.

3. The relationship was calculated for subsets of patients with specific characteristics and any potential differences between these subsets were determined.

4. Differently adjusted models might affect the progression of vascular outcomes.

5. Different cutoff values for the apnea–hypopnea index might affect the relationship between OSA and vascular outcomes.

Introduction

Obstructive sleep apnea (OSA) affects 24% of middle-aged men and 9% of women in the United States, but daytime sleepiness was reported in 17% and 22% of these subjects, respectively [1]. OSA is an increasingly prevalent condition characterized by repetitive obstruction of the upper airway during sleep accompanied by episodic hypoxia, arousal, and sleep fragmentation [2]. Previous studies suggested that OSA was associated with an increased risk of glaucoma, diabetic kidney disease, and metabolic syndrome [3-5]. However, data on the association between OSA and the risk of subsequent vascular outcomes and mortality are both limited and inconclusive. Furthermore, whether these relationships differ according to the characteristics of patients with OSA also needs to be verified.

Several meta-analyses have illustrated that continuous positive airway pressure (CPAP) interventions aimed at OSA may reduce the risk of cardiovascular outcomes. Kim et al. [6] showed that CPAP treatment for OSA was associated with a lower incidence of stroke and cardiac events. Furthermore, Bratton et al. [7] indicated that use of both CPAP and mandibular advancement devices was associated with a reduction in the blood pressure among patients with OSA. Nadeem et al. [8] suggested that CPAP treatment for OSA seemed to improve dyslipidemia (decrease in total cholesterol and low-density lipoprotein, and increase in high-density lipoprotein), whereas it did not appear to affect the triglyceride levels. These studies demonstrated that patients with OSA who received interventions had a reduced risk of cardiovascular diseases. Therefore, clarifying the relationship between OSA and vascular outcomes is particularly important as it has not been definitively determined.

For peer review only

This study attempted to perform a large-scale examination of the available prospective studies to determine the association of OSA with the potential risk of vascular outcomes and all-cause mortality.

Methods

Data sources, search strategy, and selection criteria

This study was conducted and reported according to the Meta-analysis of Observational Studies in Epidemiology protocol [9].

Any prospective cohort study that examined the relationship between OSA and vascular outcomes or all-cause mortality was eligible for inclusion into this study, and no restrictions were placed on language or publication status (e.g., published, in press, or in progress). Electronic databases PubMed, Embase, and the Cochrane Library were searched for articles published through May 2016, using the terms "sleep apnea" OR "obstructive sleep apneas" AND ("cardiovascular disease" OR "stroke" OR "cardiac death" OR "mortality" OR "death" OR "CVD" OR "myocardial infarction" OR "coronary events") AND "clinical trials" AND "human" as the search terms (Supplemental 1). Manual searches of reference lists were also conducted from all the relevant original and reviewed articles to identify additional eligible studies. The medical subject heading, methods, patient population, design, exposure, and outcome variables of these articles were used to identify the relevant studies.

The literature search was independently undertaken by two authors using a standardized approach. Any inconsistencies between these two authors were settled

For peer review only

by the primary author until a consensus was reached. The study was eligible for inclusion if the following criteria were met: (1) the study had a prospective cohort design; (2) the study investigated the association between OSA and the risk of major adverse cardiac events (MACEs), coronary heart disease (CHD), stroke, cardiac death, all-cause mortality, and heart failure; and (3) the authors reported effect estimates [relative risk (RR), hazard ratio (HR), or odds ratio (OR)] and 95% confidence intervals (CIs) for comparisons of different levels of OSA versus lowest OSA level. All case-control studies were excluded because various confounding factors could bias the results.

Data collection and quality assessment

The data collected included the first author's name, publication year, country, sample size, mean age at baseline, percentage of male patients, body mass index (BMI), disease status, assessment of OSA, follow-up duration, effect estimate and its 95% CI, reported endpoints, and covariates in the fully adjusted model. For studies that reported several multivariable adjusted RRs, the effect estimate that was maximally adjusted for potential confounders was selected.

The Newcastle–Ottawa Scale (NOS), which is quite comprehensive and has been partially validated for evaluating the quality of observational studies in the meta-analysis, was used to evaluate the methodological quality [10]. The NOS is based on the following three subscales: selection (4 items), comparability (1 item), and outcome (3 items). A "star system" (range, 0–9) was developed for assessment

For peer review only

(Table 1). The data extraction and quality assessment were conducted independently by two authors. Information was examined and adjudicated independently by an additional author referring to the original studies.

Statistical analysis

The relationship between OSA and the risk of vascular outcomes or all-cause mortality based on the effect estimate (OR, RR, or HR) and its 95% CI was examined in each study. HR was considered to be equivalent to RR in cohort studies. Given the low incidence of vascular outcomes and all-cause mortality, ORs could be considered as accurate estimates of RRs [11]. A semi-parametric method was first used to evaluate the association of mild OSA [apnea–hypopnea index (AHI): 5–15], moderate OSA (AHI: 15–30) and severe OSA (AHI > 30) with the risk of vascular outcomes or all-cause mortality in order to analyze the trend between OSA levels and vascular outcomes or all-cause mortality risk [12]. For each individual study, each category of AHI was reclassified based on its calculated mid-point (for closed categories) or median (for open categories, assuming a normal distribution for AHI). The control category was composed of participants with the lowest AHI or normal participants in that study. Furthermore, when an individual study provided more than one median AHI level for classification among the three categories (i.e. mild, moderate or severe OSA), a fixed-effects model was used to calculate their summary RRs and 95% CIs to obtain effect estimates for each category [13]. If the study data were not broken down by AHI but rather by oxygen desaturation index (ODI), classification into the OSA categories was carried out based on the judgment of the clinicians. A random-effects

For peer review only

model was then used to calculate summary RRs and 95% CIs for mild, moderate, and severe OSA versus normal [14]. Finally, the ratio of RRs between subgroups (and the corresponding 95% CIs) were estimated using specific RRs and 95% CIs after considering the country, mean age, gender, BMI, disease status, and duration of the follow-up period [15].

Heterogeneity between studies was investigated using the Q statistic, and *P* values <0.10 was considered as indicative of significant heterogeneity [16 17]. Subgroup analyses were conducted for mild, moderate, and severe OSA and the risk of MACEs based on the country, mean age, gender, BMI, disease status, and duration of the follow-up period. A sensitivity analysis was also performed by removing each individual study from the meta-analysis [18]. Several methods were used to check for potential publication bias. Visual inspections of funnel plots for MACEs were conducted. The Egger [19] and Begg [20] tests were also used to statistically assess publication bias for MACEs. All reported *P* values were two sided, and *P* values <0.05 were considered statistically significant for all included studies. Statistical analyses were performed using the STATA software (version 12.0; Stata Corporation, TX, USA).

For peer review only

Results

Literature search

The results of the study-selection process are shown in Figure 1. An initial electronic search yielded 3282 articles, of which 3236 duplicates and irrelevant

studies were excluded, and 46 potentially eligible studies were selected. After detailed evaluations, 16 prospective studies were selected for the final meta-analysis [21-36]. No new studies qualified for inclusion after a manual search of the reference lists of these studies. The general characteristics of the included studies are presented in Table 1.

Study characteristics

A total of 16 studies with 24,308 individuals qualified for this study. The follow-up period for participants was 2.9–18.0 years, while 73–10,149 individuals were included in each study. Eight studies were conducted in the United States, four in Spain, one in Sweden, one in Portugal, one in Hungary, and one in Canada. Furthermore, 11 studies reported healthy participants, and the remaining 5 studies reported participants with different diseases. The mean BMI ranged from 26.8 to 34.0 kg/m^2. Fourteen studies used polysomnography (PSG), and the remaining one study used limited PSG to assess the levels of OSA. The study quality was assessed using the NOS (Table 1). Overall, one study had a score of 9, six studies had a score of 8, seven studies had a score of 7, and the remaining two studies had a score of 6.

For peer review only - http://bmjopen.bmj.com/site/about/guidelines.xhtml

Table 1. Baseline characteristic of studies included in the systematic review and meta-analysis

Study	Country	Sample size	Mean age	Percentage male (%)	BMI	Disease status	Assessment OSA	AHI or ODI categories	Follow-up duration (year)	Reported outcomes	Adjusted factors	NOS score
Mooe et al. 2000 [21]	Sweden	408	59.1	58.4	27.0	CAD	Limited PSG	< 5; 5-10; 10-15; ≥ 15	5.1	CHD, stroke, all-cause mortality	Age, sex, BMI, hypertension, DM, LVF, and coronary intervention	7
Gottlieb et al. 2010 [22]	USA	4422	62.4	43.5	28.2	Healthy	PSG	< 5; 5-15; 15-30; ≥ 30	8.7	HF	Age, race, BMI, smoking, DM, SBP, DBP, TC, HDL-C, lipid-lowering	8

For peer review only

											medications, and antihypertensive medications	
Campos-Rodriguez et al. 2012 [23]	Spain	1116	56.1	0.0	36.6	Healthy	PSG	< 10; 10-29; ≥ 30	6.0	Cardiac death	Age, BMI, DM, hypertension, and previous CVD	8
Marin et al. 2005 [24]	Spain	1729	49.9	100	28.7	Healthy	PSG	5-30; ≥ 30	10.1	Cardiac death and CHD	Age, diagnostic group, presence of CVD, DM, hypertension, lipid disorders, smoking, alcohol, SBP DBP,	9

For peer review only

											blood glucose, TC, TG, and use of antihypertensive, lipid-lowering and antidiabetic drugs	
Young et al. 2008 [25]	USA	1522	48.0	55.0	28.6	Healthy	PSG	5-15; 15-30; ≥ 30	18.0	Cardiac death, all-cause mortality, and CHD	Age, age-squared, sex, BMI, and BMI squared	8
Redline et al. 2010 [26]	USA	5422	62.9	45.4	27.8	Healthy	PSG	Quartile I (0-4.05); Quartile II	8.7	Stroke	Age, BMI, race, smoking, SBP, DM, and antihypertensive	8

For peer review only

								(4.05-9.50); Quartile III (9.50-19.1 3); Quartile IV (>19.13)			medications	
Arzt et al. 2005 [27]	USA	1189	47.0	55.0	30.0	Healthy	PSG	<5; 5-20; ≥ 20	4.0	Stroke	Age, sex, and BMI	7
Punjabi et al. 2008	USA	6294	62.5	47.0	27.8	Healthy	PSG	Quartile I (0-8.50);	8.2	CHD, all-cause	Age, sex, race, BMI, SBP, DBP, smoking,	8

For peer review only

[28]								Quartile II (8.51-15.09); Quartile III (15.10-24.28); Quartile IV (>24.28)		mortality	prevalent hypertension, DM, and CVD	
Shah et al. 2010 [29]	USA	1436	59.7	69.4	32.9	Healthy	PSG	<5; 5-14; 15-29; ≥	2.9	CHD, cardiac death	Age, race, sex, smoking, alcohol,	7

For peer review only

								30			BMI, AF, DM, hypertension, and hyperlipidemia	
Yaggi et al. 2005 [30]	USA	1022	60.2	71.3	32.8	Healthy	PSG	⩽3; 4-12; 13-36; ≥ 36	3.4	Stroke and all-cause mortality	Age, sex, race, smoking, alcohol, BMI, DM, hyperlipidemia, AF, and hypertension	8
Martı´nez-G arcı´a et al. 2009 [31]	Spain	166	73.3	59.0	28.1	Ischemic Stroke	PSG	0-9; 10-19; ≥ 20	5.0	All-cause mortality	Age, sex, Barthel index, AHI, and CPAP treatment groups, previous stroke or	7

For peer review only

											TIA, diabetes, hypercholesterolemia, BMI, smoking, arterial hypertension, atrial fibrillation, significant carotid stenosis, and fibrinogen levels	
Munoz et al. 2006 [32]	Spain	1034	79.8	57.0	26.8	Healthy	PSG	<30; ≥ 30	6.0	Stroke	Sex	7
Leão et al.	Portugal	73	62.4	75.0	27.6	Acute	PSG	5-15;	6.3	CHD	Sex	7

For peer review only

2016 [33]						coronary syndrome		15-30; ≥ 30				
Fornadi et al. 2014 [34]	Hungary	100	51.0	56.8	26.8	Kidney transplant recipients	PSG	5-15; 15-30; ≥ 30	6.3	All-cause mortality	Unadjusted	6
Kendzerska et al. 2014 [35]	Canada	10149	49.9	62.0	30.1	Healthy	PSG	< 5; 5-15; 15-30; ≥ 30	5.7	All-cause mortality	Traditional CV risk factors	7

For peer review only

Won et al. 2013 [36]	USA	281	65.0	98.0	34.0	Ischemic heart disease and myocardial injury	PSG	5-30; ≥ 30	4.1	All-cause mortality	NA	6

AF, atrial fibrillation; AHI: apnea–hypopnea index; BMI, body mass index; CAD, coronary artery disease; CHD, coronary heart disease; CPAP, continuous positive airway pressure; CV, cardiovascular; CVD, cardiovascular disease; DBP, diastolic blood pressure; DM, diabetes mellitus; HDL-C, high-density lipoprotein cholesterol; HF, heart failure; LVF, left ventricular function; NA, not applicable; ODI: oxygen desaturation index; OSA, obstructive sleep apnea; PSG, polysomnography; SBP, systolic blood pressure; TC, total cholesterol; TG, triglyceride; TIA, transient ischemic attack.

For peer review only

OSA and MACE risk

The summary RRs showed that mild OSA was not associated with MACEs (RR: 0.98; 95% CI: 0.87–1.11; *P* = 0.741; Fig. 2 and Table 2). Furthermore, the pooled analysis results for moderate and severe OSA indicated that they had a harmful effect on the risk of MACEs (moderate: RR, 1.16; 95% CI, 1.01–1.33; *P* = 0.034; Fig. 3 and Table 2; severe: RR, 2.04; 95% CI, 1.56–2.66; *P* < 0.001; Fig. 4 and Table 2). A subgroup analysis for MACEs was conducted to minimize heterogeneity among the included studies and evaluate the relationship between OSA and MACEs in specific subpopulations (Table 3). Overall, participants with moderate OSA were associated with an increased risk of MACEs if individuals did not have other diseases (RR: 1.16; 95% CI: 1.01–1.33; *P* = 0.034). Furthermore, no significant association was found between severe OSA and MACEs if the study included only women (RR: 1.98; 95% CI: 0.64–6.06; *P* = 0.234); in other subsets, severe OSA was associated with an increased risk of MACEs (Table 3). Finally, no evidence of a factor-specific difference was found in the RR for MACEs among participants with OSA compared with controls (Table 3).

Table 2. Summary of the relative risks of all outcomes evaluated

Outcomes	Mild OSA (RR with 95% CI)	*P* value for mild OSA	Moderate OSA (RR with 95% CI)	*P* value for moderate OSA	Severe OSA (RR with 95% CI)	*P* value for severe OSA
MACEs	0.98 (0.87–1.11)	0.741	1.16 (1.01–1.33)	0.034	2.04 (1.56–2.66)	<0.001

For peer review only

CHD	1.25 (0.95–1.66)	0.117	1.38 (1.04–1.83)	0.026	1.63 (1.18–2.26)	0.003
Stroke	1.29 (0.69–2.41)	0.424	1.35 (0.82–2.23)	0.245	2.15 (1.42–3.24)	<0.001
Cardiac death	1.80 (0.68–4.76)	0.236	1.11 (0.53–2.35)	0.781	2.96 (1.45–6.01)	0.003
All-cause mortality	1.26 (0.77–2.07)	0.354	1.04 (0.60–1.79)	0.895	1.54 (1.21–1.97)	<0.001
Heart failure	1.02 (0.78–1.34)	0.868	1.07 (0.74–1.54)	0.719	1.44 (0.94–2.21)	0.097

CHD, Coronary heart disease; CI: confidence interval; MACE, major cardiovascular event; OSA, obstructive sleep apnea; RR: relative risk.

Table 3. Subgroup analyses for MACEs

Variable	Subgroup	Mild OSA (RR with 95% CI)	*P* value for mild OSA	Moderate OSA (RR with 95% CI)	*P* value for moderate OSA	Severe OSA (RR with 95% CI)	*P* value for severe OSA
Country	USA	1.00 (0.85–1.17)	0.977	1.14 (0.99–1.32)	0.064	**1.90 (1.35–2.67)**	**<0.001**
	Other	1.02 (0.19–5.52)	0.982	1.44 (0.83–2.50)	0.198	**2.35 (1.52–3.65)**	**<0.001**
	USA vs other	0.98 (0.18–5.32)	0.982	0.79 (0.45–1.40)	0.422	0.81 (0.46–1.41)	0.453
Mean age	≥60	0.96 (0.86–1.08)	0.540	1.13 (0.97–1.33)	0.117	**1.78 (1.23–2.57)**	**0.002**
	<60	1.40 (0.73–2.70)	0.315	1.51 (0.94–2.41)	0.086	**2.31 (1.64–3.24)**	**<0.001**
	≥60 vs <60	0.69 (0.35–1.33)	0.265	0.75 (0.46–1.23)	0.252	0.77 (0.47–1.27)	0.309

For peer review only

Gender	Male	0.92 (0.73–1.15)	0.455	1.10 (0.85–1.42)	0.449	**1.81 (1.14–2.89)**	**0.012**
	Female	1.97 (0.47–8.25)	0.353	1.36 (0.67–2.76)	0.399	1.98 (0.64–6.06)	0.234
	Male vs female	0.47 (0.11–1.99)	0.304	0.81 (0.38–1.72)	0.581	0.91 (0.27–3.08)	0.885
BMI	≥30	1.75 (0.88–3.49)	0.111	1.70 (0.94–3.07)	0.079	**2.72 (1.80–4.10)**	**<0.001**
	<30	0.96 (0.86–1.07)	0.449	1.14 (0.99–1.31)	0.078	**1.80 (1.36–2.38)**	**<0.001**
	≥30 vs <30	1.82 (0.91–3.66)	0.092	1.49 (0.81–2.74)	0.198	1.51 (0.92–2.49)	0.104
Disease statues	Healthy	1.00 (0.85–1.17)	0.977	**1.16 (1.01–1.33)**	**0.034**	**2.12 (1.53–2.94)**	**<0.001**
	Other	1.02 (0.19–5.52)	0.982	–	-	**1.96 (1.01–3.81)**	**0.047**
	Healthy vs Other	0.98 (0.18–5.32)	0.982	–	-	1.08 (0.52–2.27)	0.835
Follow-up duration	≥6	0.96 (0.86–1.07)	0.449	1.14 (0.99–1.31)	0.064	**2.06 (1.43–2.95)**	**<0.001**
	<6	1.75 (0.88–3.49)	0.111	1.74 (0.87–3.49)	0.120	**2.10 (1.39–3.17)**	**<0.001**
	≥6 vs <6	0.55 (0.27–1.10)	0.092	0.66 (0.32–1.33)	0.242	0.98 (0.57–1.70)	0.945

BMI, body mass index; CI: confidence interval; OSA, obstructive sleep apnea; RR: relative risk.

OSA and CHD risk

The pooled data of meta-analysis showed that mild OSA was not associated with the risk of CHD (RR: 1.25; 95% CI: 0.95–1.66; *P* = 0.117; Table 2 and Supplemental 2), whereas moderate OSA (RR: 1.38; 95% CI: 1.04–1.83; *P* = 0.026; Table 2 and

For peer review only

Supplemental 2) and severe OSA (RR: 1.63; 95% CI: 1.18–2.26; *P* = 0.003; Table 2 and Supplemental 2) were associated with a significantly increased risk of CHD. Stratified analyses according to gender were conducted for different levels of OSA versus normal group, and it was found that patients with severe OSA had significantly increased the risk of CHD in men (RR: 1.65; 95% CI: 1.06–2.57; *P* = 0.027). No other significant differences were detected (Table 4).

Table 4. Gender difference for other outcomes

Outcome	Subgroup	Mild OSA (RR with 95% CI)	*P* value for mild OSA	Moderate OSA (RR with 95% CI)	*P* value for moderate OSA	Severe OSA (RR with 95% CI)	*P* value for severe OSA
CHD	Men	0.93 (0.72–1.21)	0.596	1.09 (0.80–1.48)	0.582	**1.65 (1.06–2.57)**	**0.027**
	Women	1.92 (0.43–8.64)	0.394	1.51 (0.38–5.97)	0.559	1.10 (0.12–9.87)	0.933
	Men vs women	0.48 (0.11–2.22)	0.351	0.72 (0.18–2.96)	0.651	1.50 (0.16–14.22)	0.724
Stroke	Men	1.86 (0.67–5.14)	0.232	1.86 (0.70–4.95)	0.214	**2.86 (1.10–7.41)**	**0.031**
	Women	1.34 (0.76–2.36)	0.311	1.20 (0.67–2.15)	0.542	1.21 (0.65–2.25)	0.546
	Men vs women	1.39 (0.43–4.45)	0.581	1.55 (0.50–4.84)	0.451	2.36 (0.76–7.38)	0.138
Cardiac death	Men	–	–	1.15 (0.41–3.23)	0.791	**2.87 (1.13–7.27)**	**0.026**
	Women	–	–	0.94 (0.19–4.61)	0.939	3.71 (0.41–33.87)	0.245

	Men vs women	–	–	1.22 (0.18–8.17)	0.935	0.77 (0.07–8.49)	0.834
All-cause mortality	Men	–	–	–	–	**1.72 (1.22–2.43)**	**0.002**
	Women	–	–	–	–	**3.50 (1.23–9.97)**	**0.019**
	Men vs women	–	–	–	–	0.49 (0.16–1.48)	0.206
Heart failure	Men	0.88 (0.57–1.35)	0.561	1.13 (0.68–1.88)	0.639	1.58 (0.93–2.67)	0.088
	Women	1.13 (0.80–1.60)	0.493	1.01 (0.60–1.70)	0.970	1.19 (0.56–2.52)	0.650
	Men vs women	0.78 (0.45–1.35)	0.376	1.12 (0.54–2.32)	0.762	1.33 (0.53–3.33)	0.545

CHD, coronary heart disease; OSA, obstructive sleep apnea.

OSA and stroke risk

Pooled analysis results indicated no association between mild OSA (RR: 1.29; 95% CI: 0.69–2.41; $P = 0.424$; Table 2 and Supplemental 2) and moderate OSA (RR: 1.35; 95% CI: 0.82–2.23; $P = 0.245$; Table 2 and Supplemental 2) and stroke, whereas severe OSA was associated with an increased risk of stroke (RR: 2.15; 95% CI: 1.42–3.24; $P < 0.001$; Table 2 and Supplemental 2). Subgroup analysis on the basis of gender indicated that severe OSA had a harmful effect on the risk of stroke in men (RR: 2.86; 95% CI: 1.10–7.41; $P = 0.031$; Table 4).

OSA and cardiac death risk

The summary RRs showed that mild OSA (RR: 1.80; 95% CI: 0.68–4.76; $P = 0.236$; Table 2 and Supplemental 2) and moderate OSA (RR: 1.11; 95% CI: 0.53–2.35;

For peer review only

P = 0.781; Table 2 and Supplemental 2) were not associated with cardiac death risk, whereas severe OSA significantly increased the risk of cardiac death (RR: 2.96; 95% CI: 1.45–6.01; P = 0.003; Table 2 and Supplemental 2). Subgroup analysis showed that severe OSA was associated with an increased risk of cardiac death in men (RR: 2.87; 95% CI: 1.13–7.27; P = 0.026; Table 4).

OSA and all-cause mortality risk

No significant association was found between mild OSA (RR: 1.26; 95% CI: 0.77–2.07; P = 0.354; Table 2 and Supplemental 2), moderate OSA (RR: 1.04; 95% CI: 0.60–1.79; P = 0.895; Table 2 and Supplemental 2), and all-cause mortality risk. However, severe OSA had a harmful impact on the all-cause mortality (RR: 1.54; 95% CI: 1.21–1.97; P < 0.001; Table 2 and Supplemental 2). Stratified analysis suggested that severe OSA increased the risk of all-cause mortality in men (RR: 1.72; 95% CI: 1.22–2.43; P = 0.002) and women (RR: 3.50; 95% CI: 1.23–9.97; P = 0.019; Table 4).

OSA and heart failure risk

The summary results indicated no significant differences between mild OSA (RR: 1.02; 95% CI: 0.78–1.34; P = 0.868), moderate OSA (RR: 1.07; 95% CI: 0.74–1.54; P = 0.719), and severe OSA (RR: 1.44; 95% CI: 0.94–2.21; P = 0.097), and the risk of heart failure (Table 2 and Supplemental 2). Subgroup analysis reported similar results compared with the overall analysis (Table 4).

Publication bias

For peer review only

Review of the funnel plots could not rule out the potential publication bias for MACEs (Fig. 5). The Egger and Begg test results showed no evidence of publication bias for MACEs of mild OSA (*P* value for Egger: 0.132; *P* value for Begg: 0.221) and moderate OSA (*P* value for Egger: 0.052; *P* value for Begg: 0.452). Although the Begg test showed no evidence of publication bias for MACEs of severe OSA (P = 0.118), the Egger test showed potential evidence of publication bias for MACEs of severe OSA (P < 0.001). The conclusion did not change after adjustment for publication bias using the trim-and-fill method [37].

Discussion

The present study was based on prospective cohort studies and explored all possible correlations between OSA and the outcomes of MACEs, CHD, stroke, cardiac death, all-cause mortality, and heart failure. This large quantitative study included 24,308 individuals from 16 prospective cohort studies with a broad range of populations. The findings from the present meta-analysis suggested that mild OSA had no significant impact on the risk of vascular outcomes and all-cause mortality, moderate OSA was associated with an increased risk of MACEs and CHD, and severe OSA had a harmful effect on the risk of MACEs, CHD, stroke, cardiac death, and all-cause mortality.

A previous meta-analysis suggested that OSA was associated with stroke, but its relationship with ischemic heart disease and cardiovascular mortality needs further research [38]. However, this study could not illustrate the impact of different levels of OSA on the risk of serious cardiovascular outcomes. Further, Dong et al. suggested

For peer review only - http://bmjopen.bmj.com/site/about/guidelines.xhtml

that moderate-to-severe OSA significantly increased the risk of cardiovascular diseases, in particular, the risk of stroke [39]. Similarly, Ge et al. indicated that severe OSA is a strong independent predictor of cardiovascular and all-cause mortality. CPAP treatment was associated with decreased cardiovascular mortality [40]. However, these two studies could not evaluate the association of OSA with the risk of vascular outcomes and all-cause mortality in specific subpopulations. In addition, Wang et al. suggested that severe OSA significantly increased the risk of CHD and stroke, and all-cause mortality. A positive association with CHD was observed for moderate OSA but not for mild OSA [41]. However, whether this relationship differs according to the characteristics of participants remains unclear. Finally, Xie et al. conducted a meta-analysis to evaluate the relationship between OSA and recurrent vascular events and all-cause mortality [42]. However, they just compared the highest AHI versus lowest AHI, whereas the degree of OSA and subsequent adverse outcomes were not available. Therefore, a comprehensive meta-analysis of these prospective cohort studies was performed to evaluate any possible correlates between OSA and vascular outcomes.

No significant difference was observed between mild OSA and the risk of vascular outcomes. However, several studies included in this study reported inconsistent results. Young et al. suggested that mild OSA significantly increased the risk of CHD by 92% [25], whereas Punjabi et al. indicated that mild OSA might have a harmful effect on the risk of CHD [28]. This might be because these two studies used healthy individuals as controls, which may make them more susceptible to acquired

For peer review only

significant conclusion. Furthermore, most of these studies did not take into account potential confounders for the risk of cardiovascular disease. Moderate-to-severe OSA might play an important role in the risk of vascular outcomes. Shah et al. concluded that OSA increased the risk of coronary events or death from cardiovascular causes [29]. Nearly all included studies reported adverse outcomes for severe OSA. Finally, Previous studies indicated that OSA was a cause of diabetes, which was an independent risk factor for MACEs [43].

Subgroup analyses reported similar conclusions. Gender might have an impact on the relationship between OSA and CHD, stroke, or cardiac death, although the sex difference was not statistically significant. The possible reasons could be the lower prevalence of severe OSA in women and the later age of onset of OSA in women than in men. Furthermore, OSA in women always occurred after menopause. Physiological response to OSA is another reason for this nonsignificant difference. Finally, these conclusions might be unreliable because smaller cohorts were included in each subset. Therefore, further large-scale studies were needed to verify this difference. Therefore, a relative result was given, and a synthetic and comprehensive review was provided.

No significant difference was found between mild or moderate OSA and all-cause mortality, while severe OSA was associated with an increased risk of all-cause mortality. Further, these significant associations were also observed in men and women separately. Although the effect estimate in women was larger than that in men, no gender difference was found in the relationship between OSA and all-cause mortality. This might be because the number of studies that reported the relationship

For peer review only

between severe OSA and all-cause mortality was smaller than expected, and a broad 95% CI was acquired. Therefore, the association of severe OSA with all-cause mortality in women was variable and should be verified in future large-scale prospective studies.

Three strengths of this study should be highlighted. First, only prospective studies were included, which eliminated selection and recall bias, and could be of concern in retrospective case–control studies. Second, the large sample size allowed us to quantitatively assess the association of OSA with the risk of vascular outcomes and mortality, and thus the findings were potentially more robust than those of any individual study. Third, the summary RRs were calculated to evaluate any potential difference between subsets according to the characteristics of participants.

The limitations of this study were as follows: (1) the adjusted models were different across the included studies, and these factors might have played an important role in developing vascular outcomes; (2) in a meta-analysis of published studies, publication bias was an inevitable problem; and (3) the analysis used pooled data (individual data were not available), which restricted performing a more detailed relevant analysis and obtaining more comprehensive results.

The results of this study suggested that moderate-to-severe OSA might play an important role in the risk of vascular outcomes, especially for men. Future studies should focus on specific populations to analyze the gender difference to study the association between OSA and vascular outcomes.

For peer review only

Author Contributions

Chengjuan Xie carried out the studies, participated in collecting data, and drafted the manuscript. Ruolin Zhu performed the statistical analysis and participated in its design. Yanghua Tian and Kai Wang helped to draft the manuscript. All authors read and approved the final manuscript.

Conflict of interests: All authors declare no conflict of interest.

Funding statement: This research was supported by the National Basic Research Program of China (nos. 973 Program 2015CB856405 and 2012CB720704) and the National Natural Science Foundation of China (nos. 31571149, 91432301, 81301176, and 81171273).

Data sharing statement: No additional data available.

For peer review only

References

1 Young T, Palta M, Dempsey J, et al. The occurrence of sleep-disordered breathing among middle-aged adults. *N Engl J Med* 1993;**328**(17):1230-5 doi: 10.1056/NEJM199304293281704published Online First: Epub Date]|.

2 Young T, Peppard PE, Taheri S. Excess weight and sleep-disordered breathing. *J Appl Physiol (1985)* 2005;**99**(4):1592-9 doi: 10.1152/japplphysiol.00587.2005published Online First: Epub Date]|.

3 Liu S, Lin Y, Liu X. Meta-Analysis of Association of Obstructive Sleep Apnea With Glaucoma. *J Glaucoma* 2016;**25**(1):1-7 doi: 10.1097/IJG.0000000000000357published Online First: Epub Date]|.

4 Leong WB, Jadhakhan F, Taheri S, et al. The Association between Obstructive Sleep Apnea on Diabetic Kidney Disease: A Systematic Review and Meta-Analysis. *Sleep* 2016;**39**(2):301-8 doi: 10.5665/sleep.5432published Online First: Epub Date]|.

5 Xu S, Wan Y, Xu M, et al. The association between obstructive sleep apnea and metabolic syndrome: a systematic review and meta-analysis. *BMC Pulm Med* 2015;**15**:105 doi: 10.1186/s12890-015-0102-3published Online First: Epub Date]|.

6 Kim Y, Koo YS, Lee HY, et al. Can Continuous Positive Airway Pressure Reduce the Risk of Stroke in Obstructive Sleep Apnea Patients? A Systematic Review and Meta-Analysis. *PLoS One* 2016;**11**(1):e0146317 doi: 10.1371/journal.pone.0146317published Online First: Epub Date]|.

For peer review only

7 Bratton DJ, Gaisl T, Wons AM, et al. CPAP vs Mandibular Advancement Devices and Blood Pressure in Patients With Obstructive Sleep Apnea: A Systematic Review and Meta-analysis. *JAMA* 2015;**314**(21):2280-93 doi: 10.1001/jama.2015.16303published Online First: Epub Date]|.

8 Nadeem R, Singh M, Nida M, et al. Effect of CPAP treatment for obstructive sleep apnea hypopnea syndrome on lipid profile: a meta-regression analysis. *J Clin Sleep Med* 2014;**10**(12):1295-302 doi: 10.5664/jcsm.4282published Online First: Epub Date]|.

9 Stroup DF, Berlin JA, Morton SC, et al. Meta-analysis of observational studies in epidemiology: a proposal for reporting. Meta-analysis Of Observational Studies in Epidemiology (MOOSE) group. *JAMA* 2000;**283**(15):2008-12

10 Wells G, Shea B, O'Connell D. The Newcastle-Ottawa Scale (NOS) for assessing the quality of nonrandomised studies in meta-analyses. *Ottawa Hospital Research Institute* 2009

11 Zheng YL, Lian F, Shi Q, et al. Alcohol intake and associated risk of major cardiovascular outcomes in women compared with men: a systematic review and meta-analysis of prospective observational studies. *BMC Public Health* 2015;**15**:773. doi: 10.1186/s12889-015-2081-y.

12 Bradley TD, Floras JS. Obstructive sleep apnoea and its cardiovascular consequences. *Lancet* 2009;**373**(9657):82-93 doi: 10.1016/S0140-6736(08)61622-0published Online First: Epub Date]|.

13 DerSimonian R, Laird N. Meta-analysis in clinical trials. *Control Clin Trials*

For peer review only

1986;**7**(3):177-88

14 Ades AE, Lu G, Higgins JP. The interpretation of random-effects meta-analysis in decision models. *Med Decis Making* 2005;**25**(6):646-54 doi: 10.1177/0272989X05282643published Online First: Epub Date]|.

15 Huxley RR, Woodward M. Cigarette smoking as a risk factor for coronary heart disease in women compared with men: a systematic review and meta-analysis of prospective cohort studies. *Lancet* 2011;**378**(9799):1297-305 doi: 10.1016/S0140-6736(11)60781-2published Online First: Epub Date]|.

16 Deeks JJ, Higgins JP, Altman DG. *Analysing Data and Undertaking Meta‐Analyses*: John Wiley & Sons, Ltd, 2008.

17 Higgins JP, Thompson SG, Deeks JJ, et al. Measuring inconsistency in meta-analyses. *BMJ* 2003;**327**(7414):557-60 doi: 10.1136/bmj.327.7414.557published Online First: Epub Date]|.

18 Altman DG, Bland JM. Interaction revisited: the difference between two estimates. *BMJ* 2003;**326**(7382):219

19 Egger M, Davey Smith G, Schneider M, et al. Bias in meta-analysis detected by a simple, graphical test. *BMJ* 1997;**315**(7109):629-34

20 Begg CB, Mazumdar M. Operating characteristics of a rank correlation test for publication bias. *Biometrics* 1994;**50**(4):1088-101

21 Mooe T, Franklin KA, Wiklund U, et al. Sleep-disordered breathing and myocardial ischemia in patients with coronary artery disease. *Chest* 2000;**117**(6):1597-602

For peer review only

22 Gottlieb DJ, Yenokyan G, Newman AB, et al. Prospective study of obstructive sleep apnea and incident coronary heart disease and heart failure: the sleep heart health study. *Circulation* 2010;**122**(4):352-60 doi: 10.1161/CIRCULATIONAHA.109.901801published Online First: Epub Date]|.

23 Campos-Rodriguez F, Martinez-Garcia MA, de la Cruz-Moron I, et al. Cardiovascular mortality in women with obstructive sleep apnea with or without continuous positive airway pressure treatment: a cohort study. *Ann Intern Med* 2012;**156**(2):115-22 doi: 10.7326/0003-4819-156-2-201201170-00006published Online First: Epub Date]|.

24 Marin JM, Carrizo SJ, Vicente E, et al. Long-term cardiovascular outcomes in men with obstructive sleep apnoea-hypopnoea with or without treatment with continuous positive airway pressure: an observational study. *Lancet* 2005;**365**(9464):1046-53 doi: 10.1016/S0140-6736(05)71141-7published Online First: Epub Date]|.

25 Young T, Finn L, Peppard PE, et al. Sleep disordered breathing and mortality: eighteen-year follow-up of the Wisconsin sleep cohort. *Sleep* 2008;**31**(8):1071-8

26 Redline S, Yenokyan G, Gottlieb DJ, et al. Obstructive sleep apnea-hypopnea and incident stroke: the sleep heart health study. *Am J Respir Crit Care Med* 2010;**182**(2):269-77 doi: 10.1164/rccm.200911-1746OCpublished Online

For peer review only

First: Epub Date]|.

27 Arzt M, Young T, Finn L, et al. Association of sleep-disordered breathing and the occurrence of stroke. *Am J Respir Crit Care Med* 2005;**172**(11):1447-51 doi: 10.1164/rccm.200505-702OCpublished Online First: Epub Date]|.

28 Punjabi NM, Newman AB, Young TB, et al. Sleep-disordered breathing and cardiovascular disease: an outcome-based definition of hypopneas. *Am J Respir Crit Care Med* 2008;**177**(10):1150-5 doi: 10.1164/rccm.200712-1884OCpublished Online First: Epub Date]|.

29 Shah NA, Yaggi HK, Concato J, et al. Obstructive sleep apnea as a risk factor for coronary events or cardiovascular death. *Sleep Breath* 2010;**14**(2):131-6 doi: 10.1007/s11325-009-0298-7published Online First: Epub Date]|.

30 Yaggi HK, Concato J, Kernan WN, et al. Obstructive sleep apnea as a risk factor for stroke and death. *N Engl J Med* 2005;**353**(19):2034-41 doi: 10.1056/NEJMoa043104published Online First: Epub Date]|.

31 Martinez-Garcia MA, Soler-Cataluna JJ, Ejarque-Martinez L, et al. Continuous positive airway pressure treatment reduces mortality in patients with ischemic stroke and obstructive sleep apnea: a 5-year follow-up study. *Am J Respir Crit Care Med* 2009;**180**(1):36-41 doi: 10.1164/rccm.200808-1341OCpublished Online First: Epub Date]|.

32 Munoz R, Duran-Cantolla J, Martinez-Vila E, et al. Severe sleep apnea and risk of ischemic stroke in the elderly. *Stroke* 2006;**37**(9):2317-21 doi: 10.1161/01.STR.0000236560.15735.0fpublished Online First: Epub Date]|.

For peer review only

33 Leao S, Conde B, Fontes P, et al. Effect of Obstructive Sleep Apnea in Acute Coronary Syndrome. *Am J Cardiol* 2016;**117**(7):1084-7 doi: 10.1016/j.amjcard.2015.12.053published Online First: Epub Date]|.

34 Fornadi K, Ronai KZ, Turanyi CZ, et al. Sleep apnea is not associated with worse outcomes in kidney transplant recipients. *Sci Rep* 2014;**4**:6987 doi: 10.1038/srep06987published Online First: Epub Date]|.

35 Kendzerska T, Gershon AS, Hawker G, et al. Obstructive sleep apnea and risk of cardiovascular events and all-cause mortality: a decade-long historical cohort study. *PLoS Med* 2014;**11**(2):e1001599 doi: 10.1371/journal.pmed.1001599published Online First: Epub Date]|.

36 Won CH, Chun HJ, Chandra SM, et al. Severe obstructive sleep apnea increases mortality in patients with ischemic heart disease and myocardial injury. *Sleep Breath* 2013;**17**(1):85-91 doi: 10.1007/s11325-012-0653-ypublished Online First: Epub Date]|.

37 Duval S, Tweedie R. A Nonparametric "Trim and Fill" Method of Accounting for Publication Bias in Meta-Analysis. *Journal of the American Statistical Association* 2000;**95**(449):89-98

38 Loke YK, Brown JW, Kwok CS, et al. Association of obstructive sleep apnea with risk of serious cardiovascular events: a systematic review and meta-analysis. *Circ Cardiovasc Qual Outcomes* 2012;**5**(5):720-8 doi: 10.1161/CIRCOUTCOMES.111.964783published Online First: Epub Date]|.

39 Dong JY, Zhang YH, Qin LQ. Obstructive sleep apnea and cardiovascular risk:

For peer review only

meta-analysis of prospective cohort studies. *Atherosclerosis* 2013;**229**(2):489-95 doi: 10.1016/j.atherosclerosis.2013.04.026published Online First: Epub Date]|.

40 Ge X, Han F, Huang Y, et al. Is obstructive sleep apnea associated with cardiovascular and all-cause mortality? *PLoS One* 2013;**8**(7):e69432 doi: 10.1371/journal.pone.0069432published Online First: Epub Date]|.

41 Wang X, Ouyang Y, Wang Z, et al. Obstructive sleep apnea and risk of cardiovascular disease and all-cause mortality: a meta-analysis of prospective cohort studies. *Int J Cardiol* 2013;**169**(3):207-14 doi: 10.1016/j.ijcard.2013.08.088published Online First: Epub Date]|.

42 Xie W, Zheng F, Song X. Obstructive sleep apnea and serious adverse outcomes in patients with cardiovascular or cerebrovascular disease: a PRISMA-compliant systematic review and meta-analysis. *Medicine (Baltimore)* 2014;**93**(29):e336. doi: 10.1097/MD.0000000000000336.

43 Wang X, Bi Y, Zhang Q, et al. Obstructive sleep apnoea and the risk of type 2 diabetes: a meta-analysis of prospective cohort studies. *Respirology* 2013;**18**(1):140-6. doi: 10.1111/j.1440-1843.2012.02267.x.

For peer review only

Figure legends:

Figure 1. Study-selection process.

Figure 2. Association between mild OSA and MACEs.

Figure 3. Association between moderate OSA and MACEs.

Figure 4. Association between severe OSA and MACEs.

Figure 5. Funnel plots.

For peer review only

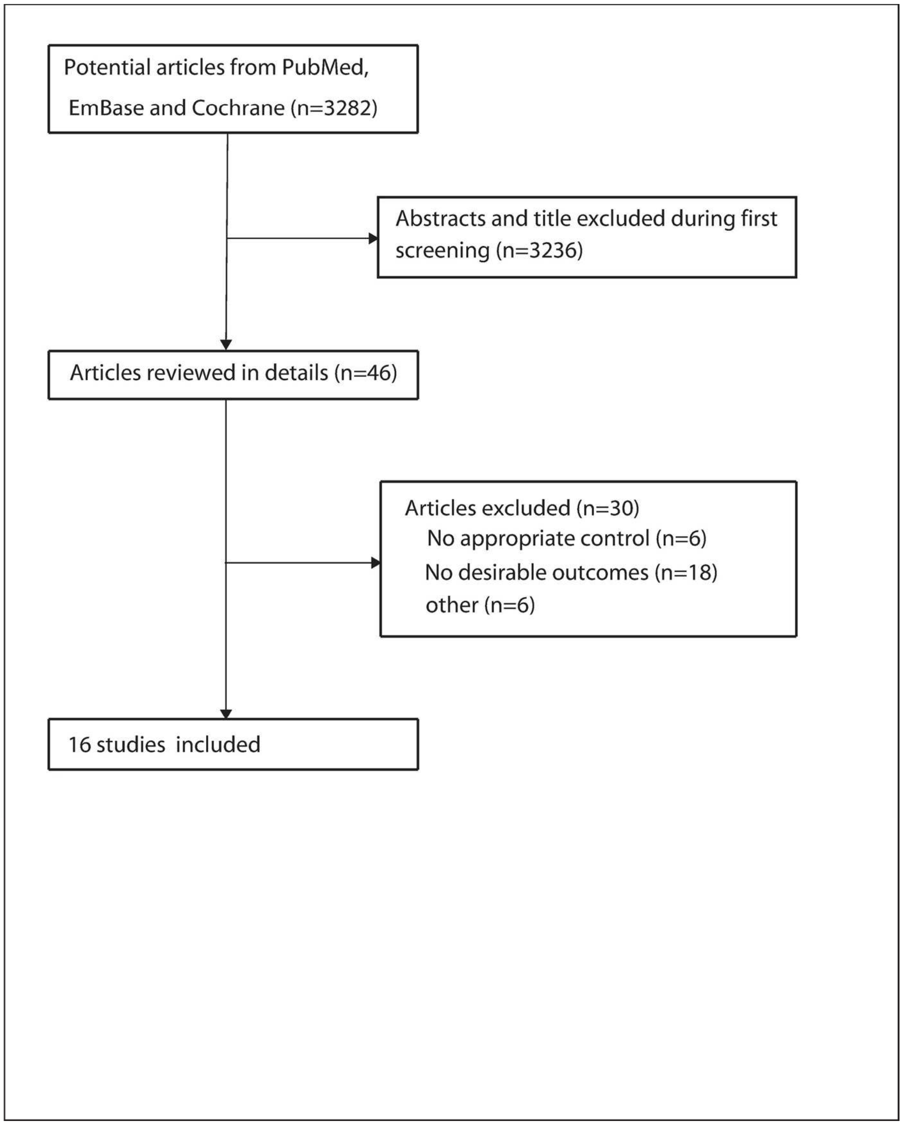

168x188mm (300 x 300 DPI)

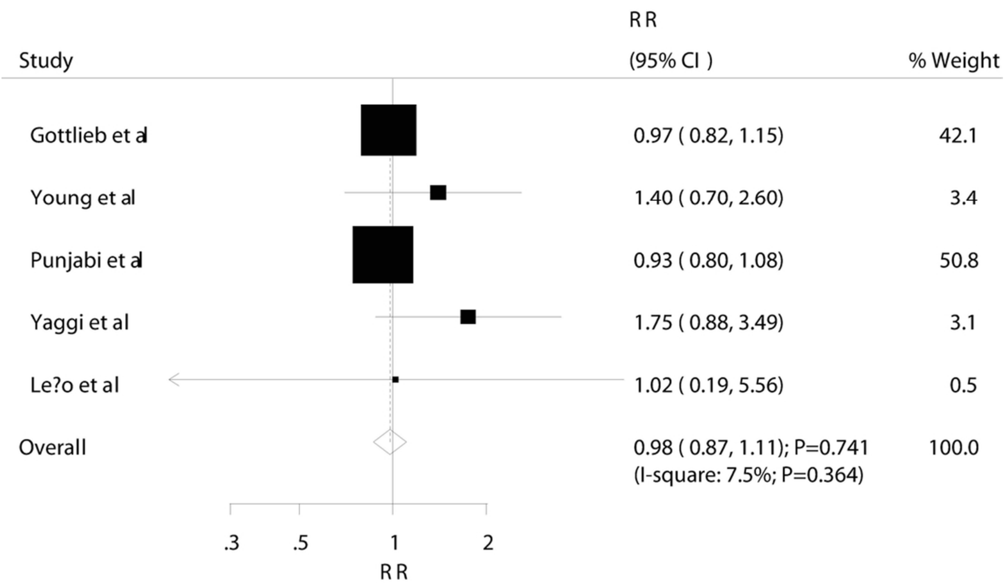

94x54mm (300 x 300 DPI)

review only

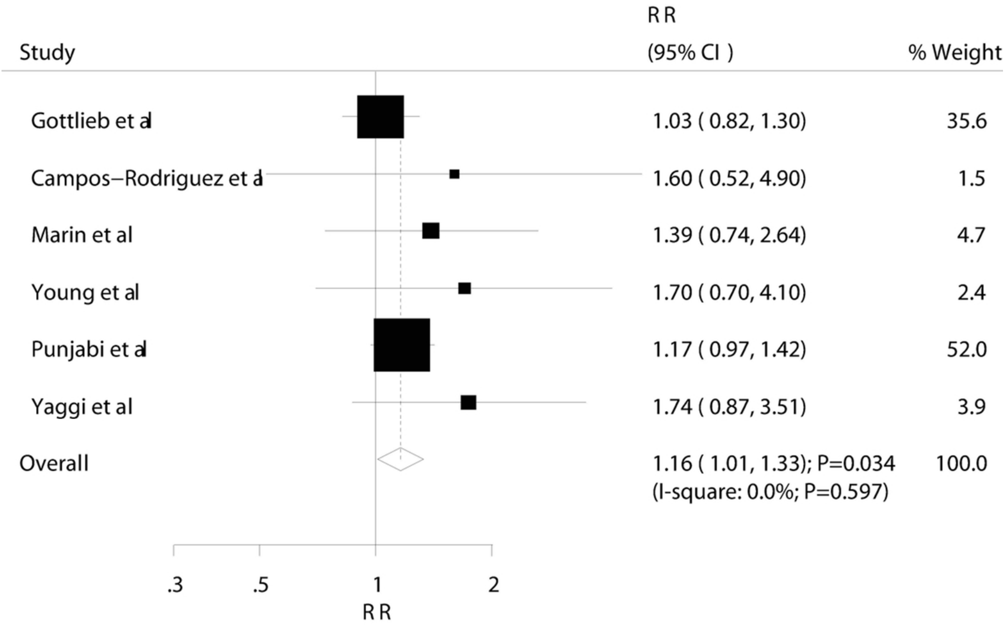

98x61mm (300 x 300 DPI)

eview only

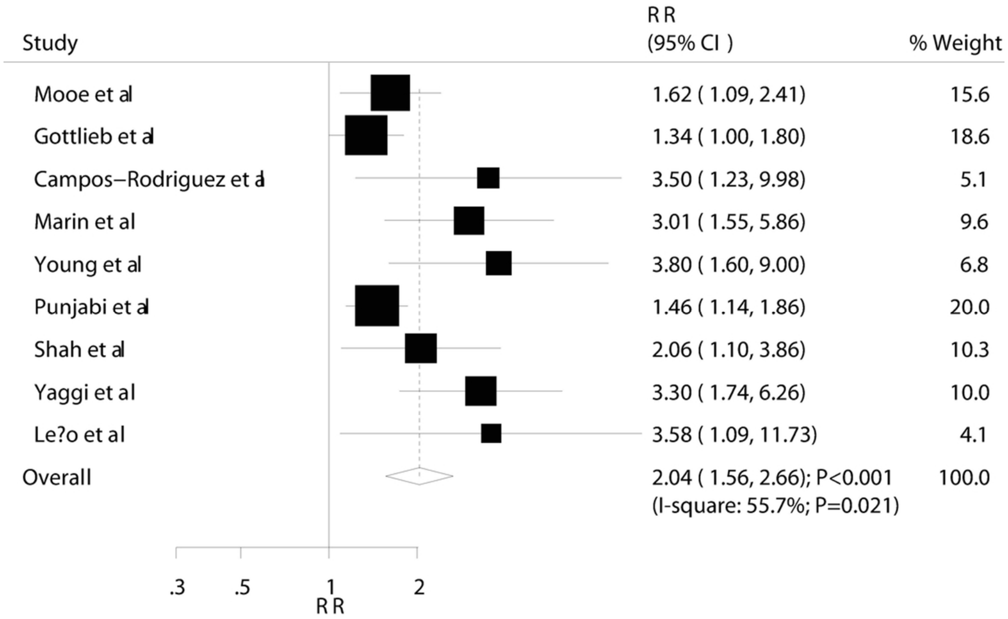

98x60mm (300 x 300 DPI)

eview only

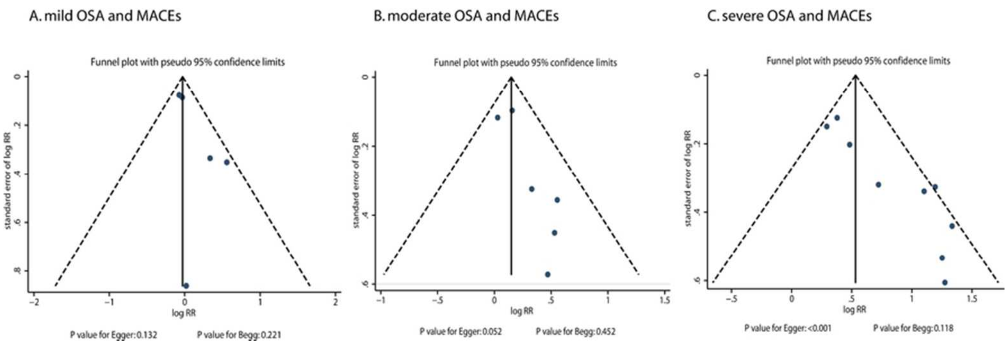

75x25mm (300 x 300 DPI)

peer review only

Title:

	**Search strategy**
**#1**	"Sleep Apnea, Obstructive" [Mesh] OR "OSA" [All fields] OR "OHS" [All fields]
**#2**	Apneas, Obstructive Sleep OR Obstructive Sleep Apneas OR Sleep Apneas, Obstructive OR Obstructive Sleep Apnea Syndrome OR Obstructive Sleep Apnea OR OSAHS OR Syndrome, Sleep Apnea, Obstructive OR Sleep Apnea Syndrome, Obstructive OR Apnea, Obstructive Sleep OR Sleep Apnea Hypopnea Syndrome OR Syndrome, Obstructive Sleep Apnea OR Upper Airway Resistance Sleep Apnea Syndrome OR Syndrome, Upper Airway Resistance, Sleep Apnea OR Hypoventilation Syndrome, Obesity OR Syndrome, Obesity Hypoventilation OR Pickwickian Syndrome OR Syndrome, Pickwickian OR Obesity-Hypoventilation Syndrome
**#3**	"Sleep Apnea Syndromes" [Mesh] OR "SAS" [All fields]
**#4**	Apnea Syndrome, Sleep OR Apnea Syndromes, Sleep OR Sleep Apnea Syndrome OR Apnea, Sleep OR Apneas, Sleep OR Sleep Apnea OR Sleep Apneas OR Sleep Hypopnea OR Hypopnea, Sleep OR Hypopneas, Sleep OR Sleep Hypopneas OR Sleep-Disordered Breathing OR Breathing, Sleep-Disordered OR Sleep Disordered Breathing OR Sleep Apnea, Mixed Central and Obstructive OR Mixed Central and Obstructive Sleep Apnea OR Sleep Apnea, Mixed OR Mixed Sleep Apnea OR Mixed Sleep Apneas OR Sleep Apneas, Mixed OR Hypersomnia with Periodic Respiration
**#5**	"Sleep Apnea, Central" [Mesh] OR "CSA"[All fields]
**#6**	Apneas, Central Sleep OR Central Sleep Apneas OR Sleep Apneas, Central OR Apnea, Central OR Apneas, Central OR Central Apnea OR Central Apneas OR Apnea, Central Sleep OR Apnea, Sleep, Central OR Sleep Apnea, Lethal Central OR Central Sleep Apnea OR Central Sleep Apnea Syndrome OR Central Sleep Disordered Breathing OR Hypoventilation, Central Alveolar OR Alveolar Hypoventilation, Central OR Alveolar Hypoventilations, Central OR Central Alveolar Hypoventilation OR Hypoventilations, Central Alveolar OR Ondine Syndrome OR Sleep-Disordered Breathing, Central OR Breathing, Central Sleep-Disordered OR Breathings, Central Sleep-Disordered OR Central Sleep-Disordered Breathing OR Central Sleep-Disordered Breathings OR Sleep Disordered Breathing, Central OR Sleep-Disordered Breathings, Central OR Central Alveolar Hypoventilation Syndrome OR Central Sleep Apnea, Secondary OR Secondary Central Sleep Apnea OR Sleep Apnea, Newborn, Primary OR Primary Sleep Apneas of Newborn OR Newborn Primary Sleep Apneas OR Central Sleep Apnea, Primary OR Primary Central Sleep Apnea
**#7**	"Continuous Positive Airway Pressure" [Mesh] OR "CPAP" [All fields] OR "Continuous Positive Airway Pressure/therapy" [Mesh]
**#8**	CPAP Ventilation OR Ventilation, CPAP OR Biphasic Continuous Positive Airway Pressure OR Bilevel Continuous Positive Airway Pressure OR Nasal Continuous Positive Airway Pressure OR nCPAP Ventilation OR Ventilation, nCPAP OR Airway Pressure Release Ventilation OR APRV Ventilation Mode OR APRV Ventilation Modes OR Ventilation Mode, APRV OR Ventilation Modes, APRV

For peer review only - http://bmjopen.bmj.com/site/about/guidelines.xhtml

**#9**	#1 OR #2 OR #3 OR #4 OR #5 OR #6 OR #7 OR #8
#10	"Cardiovascular System" [Mesh]
#11	"Death" [Mesh] OR Determination of Death OR Near-Death Experience OR Cardiac Death OR Death, Cardiac OR "Mortality" [Mesh] OR "mortality" [All fields] OR "Disease" [Mesh] OR "disease*"
**#12**	#10 AND #11
**#13**	"Cardiovascular Diseases" [Mesh] OR "CVD" OR Cardiovascular Disease OR Disease, Cardiovascular OR Diseases, Cardiovascular
**#14**	"Myocardial Infarction" [Mesh] OR "MI" OR Infarction, Myocardial OR Infarctions, Myocardial OR Myocardial Infarctions OR Cardiovascular Stroke OR Cardiovascular Strokes OR Stroke, Cardiovascular OR Strokes, Cardiovascular OR Heart Attack OR Heart Attacks OR Myocardial Infarct OR Infarct, Myocardial OR Infarcts, Myocardial OR Myocardial Infarcts
**#15**	"Angina Pectoris" [Mesh] OR "Angina, Stable" [Mesh] OR "Microvascular Angina" [Mesh] OR "Angina, Unstable" [Mesh] OR Stenocardia OR Stenocardias OR Angor Pectoris OR "angina" [All fields] OR "Coronary Artery Disease" [Mesh] OR "CAD" OR "ischemic heart disease" [All fields] OR "Heart Failure" [Mesh] OR "Heart Failure, Diastolic" [Mesh] OR "Heart Failure, Systolic" [Mesh]
**#16**	"Cerebrovascular Disorders" [Mesh] OR "cerebrovascular" [All fields] OR "stroke*"
**#17**	"Death" [Mesh] OR Determination of Death OR Near-Death Experience OR Cardiac Death OR Death, Cardiac OR "Mortality" [Mesh] OR "mortality" [All fields] OR "mortality*"
**#18**	#12 OR #13 OR #14 OR #15 OR #16 OR #17
**#19**	"Prospective Studies" [Mesh] OR "Cohort Studies" [Mesh] OR "Follow-Up Studies" [Mesh] OR "prospective study" OR "cohort study" OR "follow-up study"
**#20**	#9 AND #18 AND #19

For peer review only

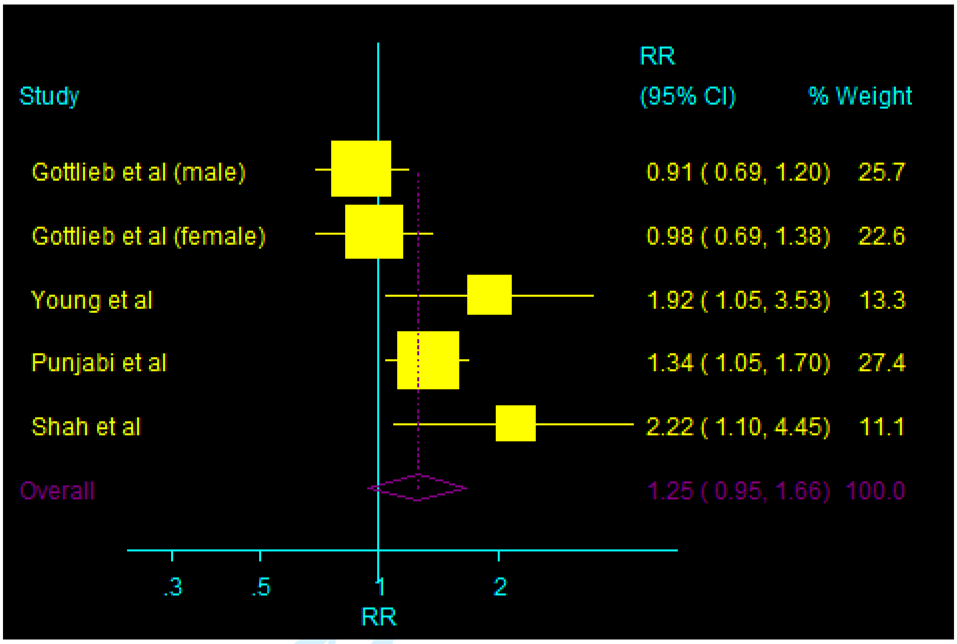

Figure S1. Association between mild OSA and CHD.

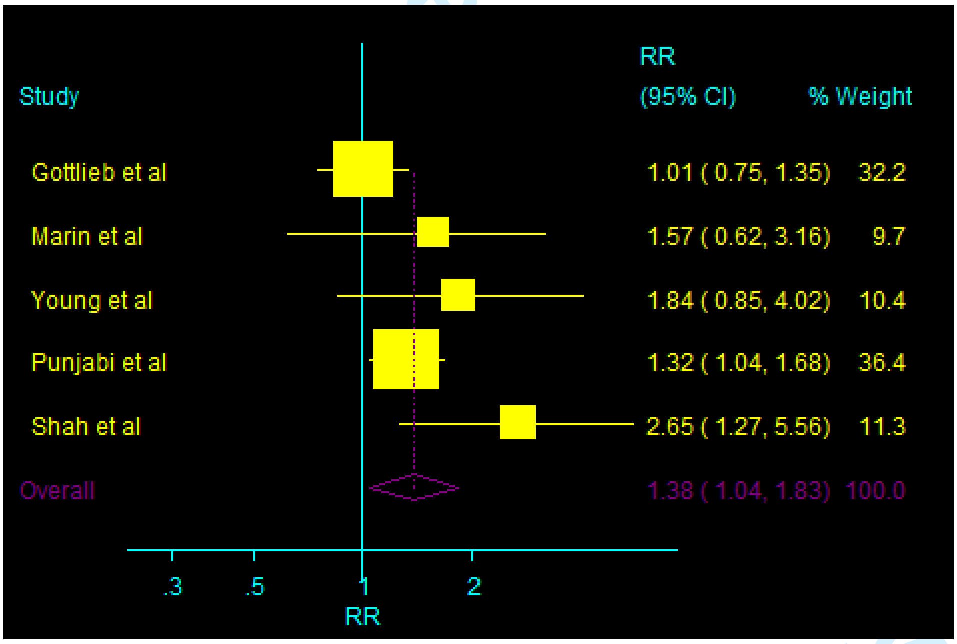

Figure S2. Association between moderate OSA and CHD.

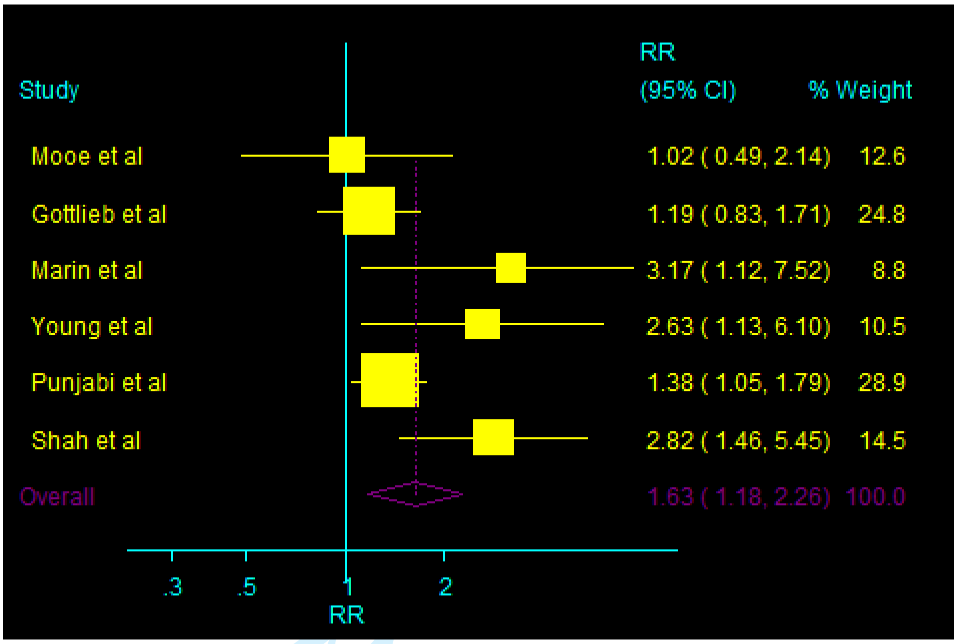

Figure S3. Association between severe OSA and CHD.

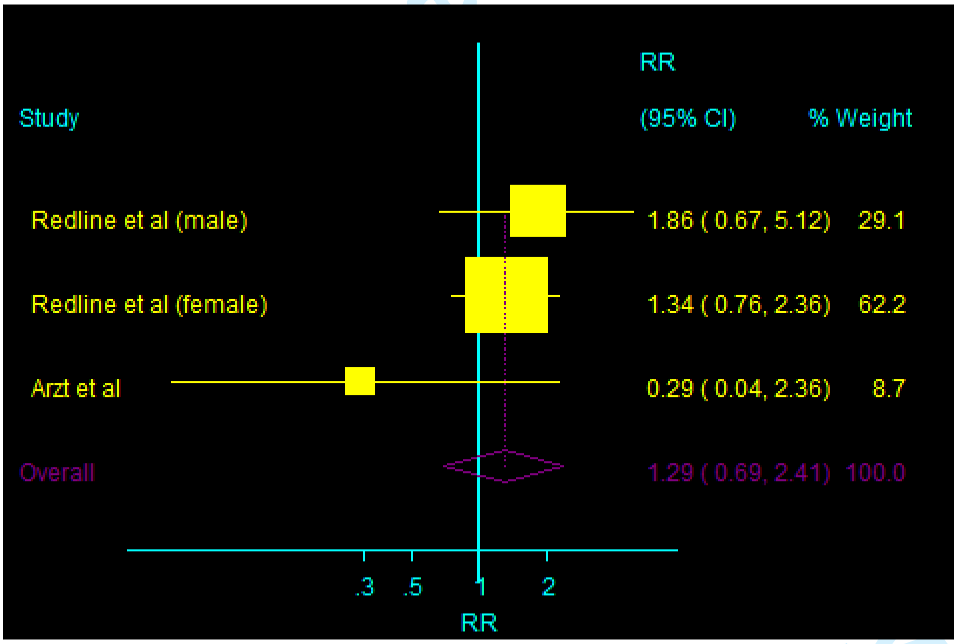

Figure S4. Association between mild OSA and stroke.

For peer review only - http://bmjopen.bmj.com/site/about/guidelines.xhtml

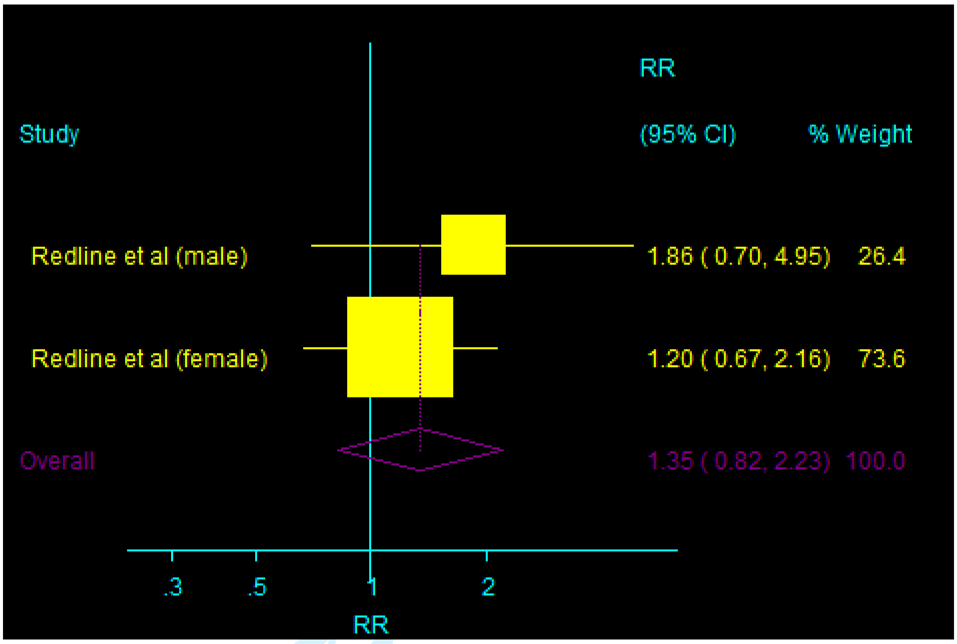

Figure S5. Association between moderate OSA and stroke.

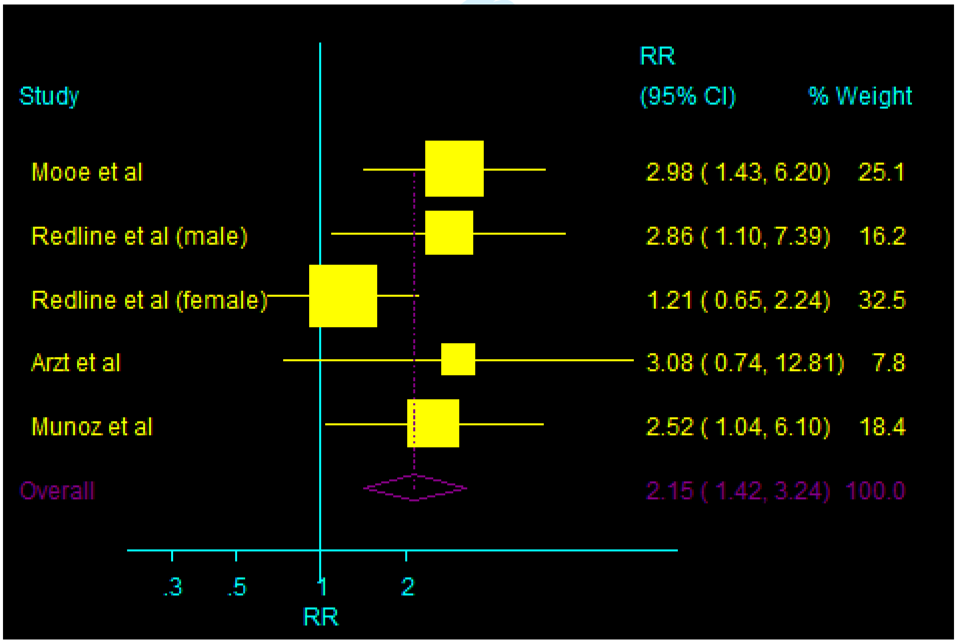

Figure S6. Association between severe OSA and stroke

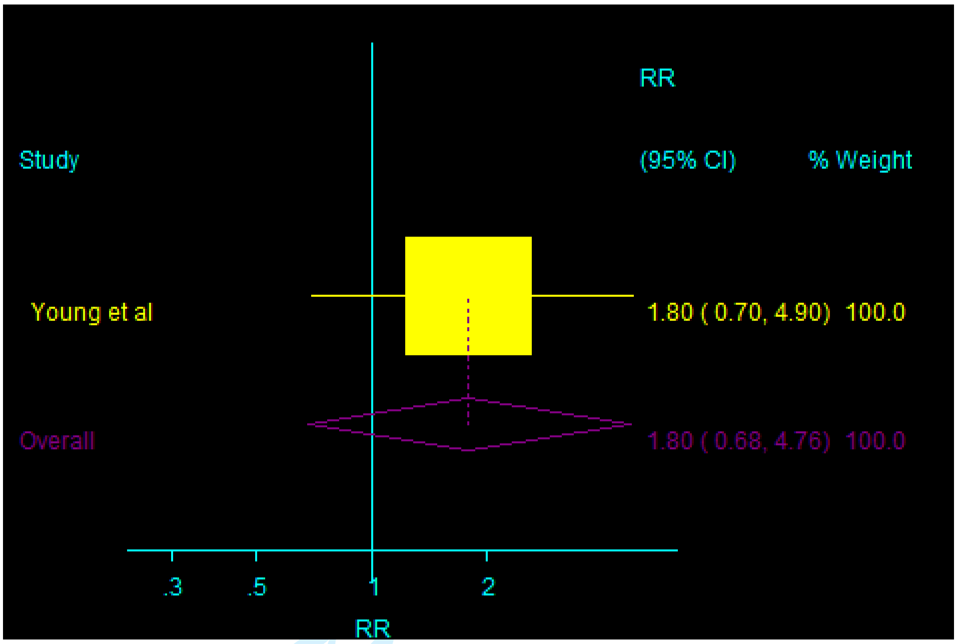

Figure S7. Association between mild OSA and cardiac death.

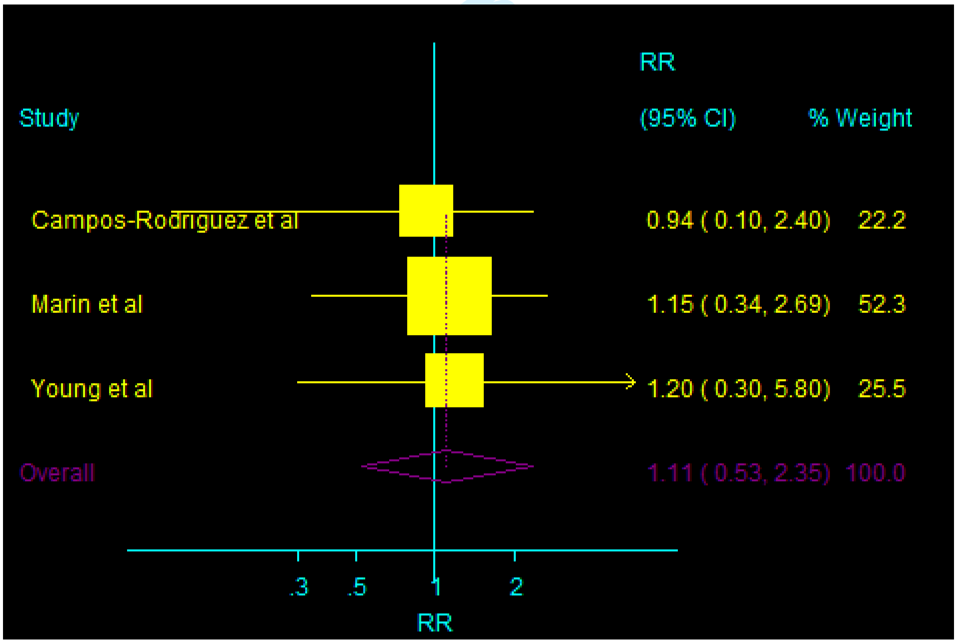

Figure S8. Association between moderate OSA and cardiac death.

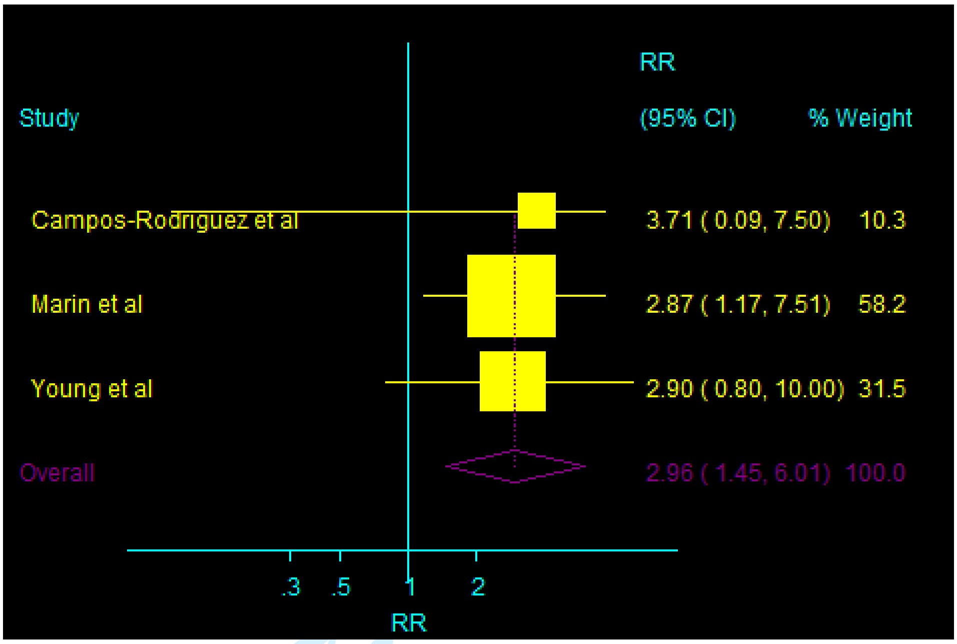

Figure S9. Association between severe OSA and cardiac death.

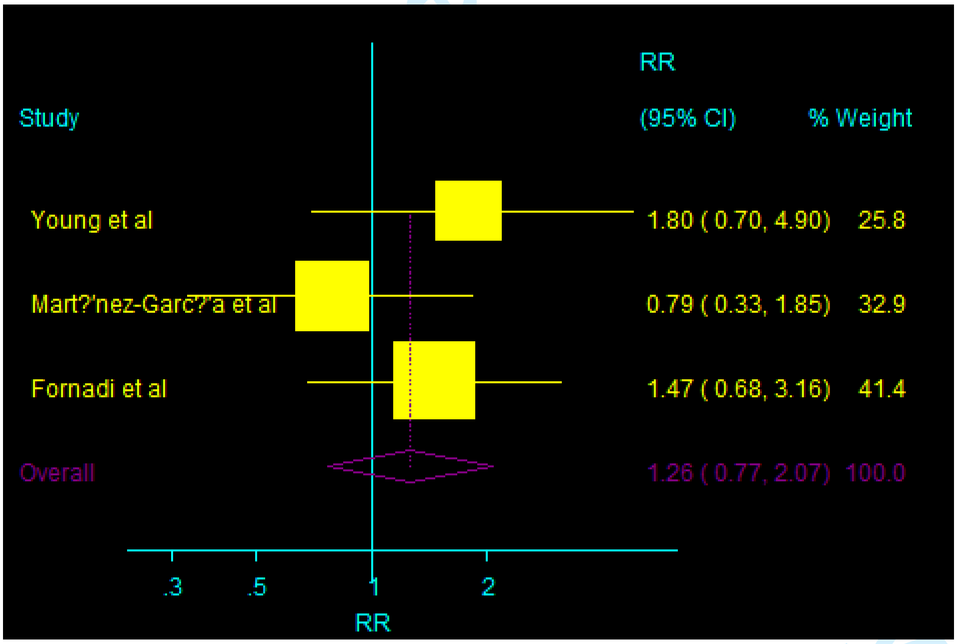

Figure S10. Association between mild OSA and all-cause death.

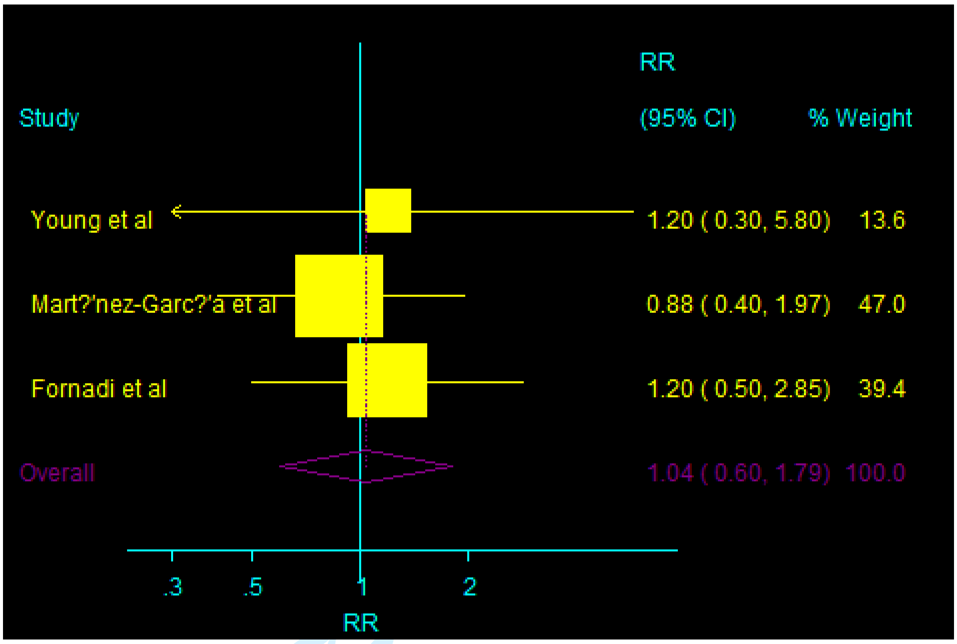

Figure S11. Association between moderate OSA and all-cause death.

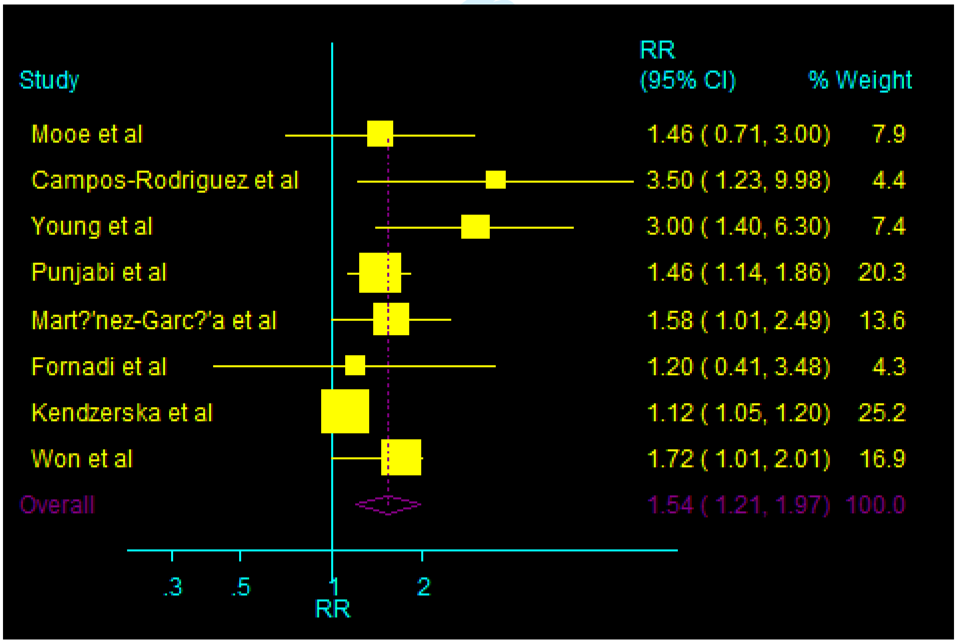

Figure S12. Association between severe OSA and all-cause death.

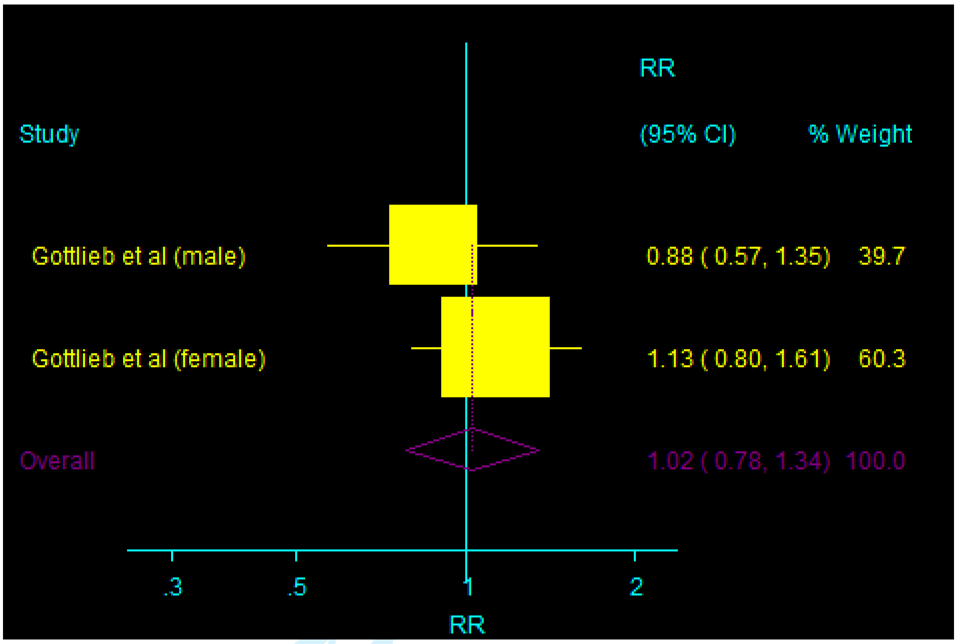

Figure S13. Association between mild OSA and heart failure.

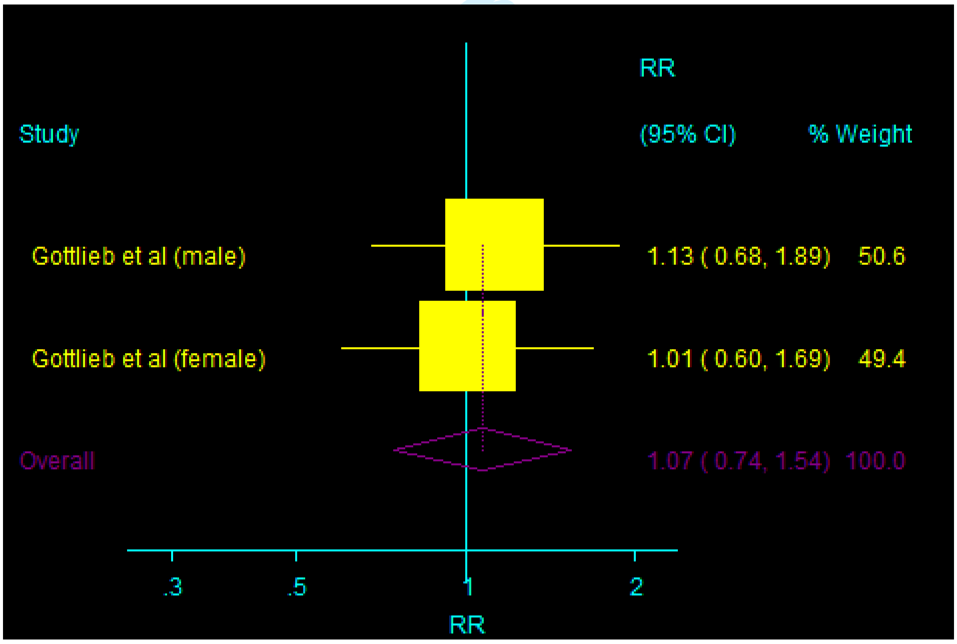

Figure S14. Association between moderate OSA and heart failure.

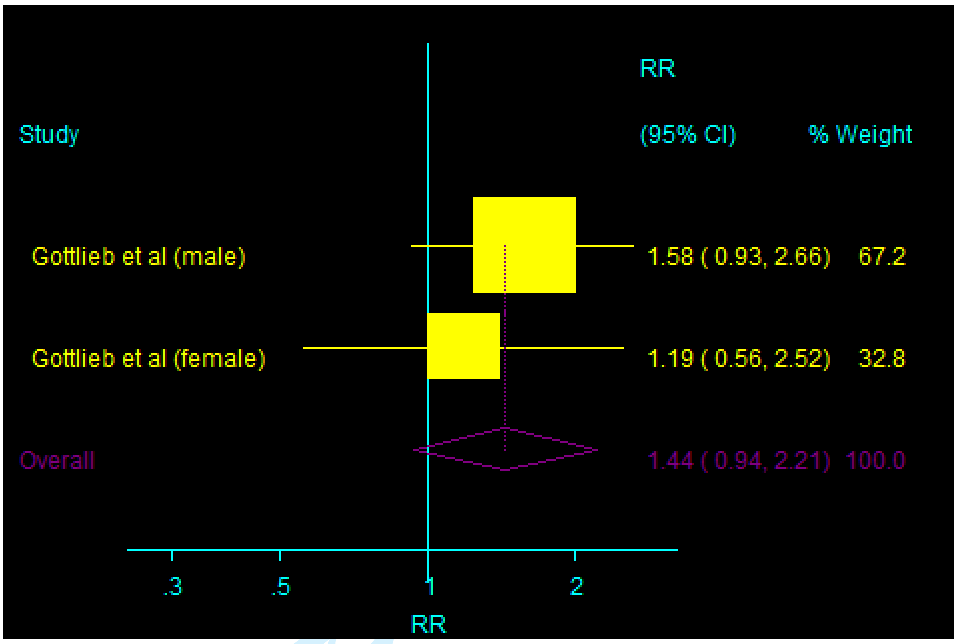

Figure S15. Association between severe OSA and heart failure.

For peer review only

STROBE 2007 (v4) checklist of items to be included in reports of observational studies in epidemiology*

Checklist for cohort, case-control, and cross-sectional studies (combined)

Section/Topic	Item #	Recommendation	Reported on page #
**Title and abstract**	1	(*a*) Indicate the study's design with a commonly used term in the title or the abstract	1
		(*b*) Provide in the abstract an informative and balanced summary of what was done and what was found	2
**Introduction**			3–4
Background/rationale	2	Explain the scientific background and rationale for the investigation being reported	3
Objectives	3	State specific objectives, including any pre-specified hypotheses	4
**Methods**			4–7
Study design	4	Present key elements of study design early in the paper	4
Setting	5	Describe the setting, locations, and relevant dates, including periods of recruitment, exposure, follow-up, and data collection	
Participants	6	(*a*) *Cohort study*—Give the eligibility criteria, and the sources and methods of selection of participants. Describe methods of follow-up<br>*Case-control study*—Give the eligibility criteria, and the sources and methods of case ascertainment and control selection. Give the rationale for the choice of cases and controls<br>*Cross-sectional study*—Give the eligibility criteria, and the sources and methods of selection of participants	
		(*b*) *Cohort study*—For matched studies, give matching criteria and number of exposed and unexposed<br>*Case-control study*—For matched studies, give matching criteria and the number of controls per case	
Variables	7	Clearly define all outcomes, exposures, predictors, potential confounders, and effect modifiers. Give diagnostic criteria, if applicable	
Data sources/ measurement	8*	For each variable of interest, give sources of data and details of methods of assessment (measurement). Describe comparability of assessment methods if there is more than one group	4
Bias	9	Describe any efforts to address potential sources of bias	
Study size	10	Explain how the study size was arrived at	
Quantitative variables	11	Explain how quantitative variables were handled in the analyses. If applicable, describe which groupings were chosen and why	5–6
Statistical methods	12	(*a*) Describe all statistical methods, including those used to control for confounding	6
		(*b*) Describe any methods used to examine subgroups and interactions	6
		(*c*) Explain how missing data were addressed	7
		(*d*) *Cohort study*—If applicable, explain how loss to follow-up was addressed<br>*Case-control study*—If applicable, explain how matching of cases and controls was addressed	7

For peer review only

		Cross-sectional study—If applicable, describe analytical methods taking account of sampling strategy	
		(*e*) Describe any sensitivity analyses	
**Results**			7-20
Participants	13*	(a) Report numbers of individuals at each stage of study—eg numbers potentially eligible, examined for eligibility, confirmed eligible, included in the study, completing follow-up, and analysed	
		(b) Give reasons for non-participation at each stage	
		(c) Consider use of a flow diagram	
Descriptive data	14*	(a) Give characteristics of study participants (eg demographic, clinical, social) and information on exposures and potential confounders	
		(b) Indicate number of participants with missing data for each variable of interest	
		(c) *Cohort study*—Summarise follow-up time (eg, average and total amount)	
Outcome data	15*	*Cohort study*—Report numbers of outcome events or summary measures over time	
		Case-control study—Report numbers in each exposure category, or summary measures of exposure	
		Cross-sectional study—Report numbers of outcome events or summary measures	
Main results	16	(*a*) Give unadjusted estimates and, if applicable, confounder-adjusted estimates and their precision (eg, 95% confidence interval). Make clear which confounders were adjusted for and why they were included	
		(*b*) Report category boundaries when continuous variables were categorized	
		(*c*) If relevant, consider translating estimates of relative risk into absolute risk for a meaningful time period	
Other analyses	17	Report other analyses done—eg analyses of subgroups and interactions, and sensitivity analyses	
**Discussion**			21-23
Key results	18	Summarise key results with reference to study objectives	22
Limitations	19	Discuss limitations of the study, taking into account sources of potential bias or imprecision. Discuss both direction and magnitude of any potential bias	
Interpretation	20	Give a cautious overall interpretation of results considering objectives, limitations, multiplicity of analyses, results from similar studies, and other relevant evidence	
Generalisability	21	Discuss the generalisability (external validity) of the study results	23
**Other information**			
Funding	22	Give the source of funding and the role of the funders for the present study and, if applicable, for the original study on which the present article is based	24

*Give information separately for cases and controls in case-control studies and, if applicable, for exposed and unexposed groups in cohort and cross-sectional studies.

Note: An Explanation and Elaboration article discusses each checklist item and gives methodological background and published examples of transparent reporting. The STROBE checklist is best used in conjunction with this article (freely available on the Web sites of PLoS Medicine at http://www.plosmedicine.org/, Annals of Internal Medicine at http://www.annals.org/, and Epidemiology at http://www.epidem.com/). Information on the STROBE Initiative is available at www.strobe-statement.org.

For peer review only

MOOSE Statement: Reporting Checklist for Authors, Editors, and Reviewers of Meta-analyses of Observational Studies

Reporting Criteria	Reported (Yes/No)	Reported on Page
**Reporting of background should include**		
Problem definition	Yes	3
Hypothesis statement	Yes	3
Description of study outcomes	Yes	3
Type of exposure or intervention used	Yes	3 – 4
Type of study designs used	Yes	4
Study population	Yes	4
**Reporting of search strategy should include**		
Qualifications of searchers (e.g., librarians and investigators)	Yes	4 – 5
Search strategy, including time period used in the synthesis and key words	Yes	5
Effort to include all available studies, including contact with authors	Yes	5
Databases and registries searched	Yes	4–5
Search software used, name and version, including special features used (e.g., explosion)	Yes	4–5
Use of hand searching (e.g., reference lists of obtained articles)	Yes	5
List of citations located and those excluded, including justification	Yes	8
Method of addressing articles published in languages other than English	Yes	4
Method of handling abstracts and unpublished studies	Yes	4–5
Description of any contact with authors	No	NA
**Reporting of methods should include**		
Description of relevance or appropriateness of studies assembled for assessing the hypothesis to be tested	No	5
Rationale for the selection and coding of data (e.g., sound clinical principles or convenience)	Yes	5–6
Documentation of how data were classified and coded (e.g., multiple raters, blinding and inter-rater reliability)	Yes	5–6
Assessment of confounding (e.g., comparability of cases and controls in studies where appropriate)	Yes	6
Assessment of study quality, including blinding of quality assessors, and stratification or regression on possible predictors of study results	Yes	6

For peer review only

Assessment of heterogeneity	Yes	7
Description of statistical methods (e.g., complete description of fixed- or random-effects models, justification of whether the chosen models account for predictors of study results, dose–response models, or cumulative meta-analysis) in sufficient detail to be replicated	Yes	6–7
Provision of appropriate tables and graphics	Yes	6–7
**Reporting of results should include**		
Graphic summarizing individual study estimates and overall estimate	Yes	8
Table giving descriptive information for each study included	Yes	8–14
Results of sensitivity testing (e.g., subgroup analysis)	Yes	15–20
Indication of statistical uncertainty of findings	Yes	20
**Reporting of discussion should include**		
Quantitative assessment of bias (e.g., publication bias)	Yes	20
Justification for exclusion (e.g., exclusion of non-English language citations)	No	21
Assessment of quality of included studies	Yes	Table 1
Strengths and weaknesses	Yes	23
**Reporting of conclusions should include**		
Consideration of alternative explanations for observed results	Yes	20–23
Generalization of the conclusions (e.g., appropriate for the data presented and within the domain of the literature review)	Yes	23
Guidelines for future research	Yes	23
Disclosure of funding source	Yes	24

NA, Not applicable.

For peer review only

Association of obstructive sleep apnea with the risk of vascular outcomes and all-cause mortality: a meta-analysis

Journal:	*BMJ Open*
Manuscript ID	bmjopen-2016-013983.R3
Article Type:	Research
Date Submitted by the Author:	10-Oct-2017

Complete List of Authors:	Xie, Chengjuan; The First Affiliated Hospital of Anhui Medical University, Department of Neurology<br>Zhu, Ruolin; The First Affiliated Hospital of Anhui Medical University, Department of Neurology<br>Tian, Yanghua; The First Affiliated Hospital of Anhui Medical University, Department of Neurology<br>Wang, Kai; Department of Neurology

**Primary Subject Heading**:	Neurology
Secondary Subject Heading:	Cardiovascular medicine
Keywords:	meta-analysis, mortality, obstructive sleep apnea, vascular outcome

SCHOLARONE™ Manuscripts

For peer review only

Association of obstructive sleep apnea with the risk of vascular outcomes and all-cause mortality: a meta-analysis

Chengjuan Xie[1], Ruolin Zhu[1], Yanghua Tian[1,2], Kai Wang[1, 2, 3,*]

[1]Department of Neurology, The First Affiliated Hospital of Anhui Medical University, Hefei, China

[2]Collaborative Innovation Centre of Neuropsychiatric Disorders and Mental Health, Anhui Province, China

[3]Department of Medical Psychology, Anhui Medical University, Hefei, China

***Correspondence author:**

Kai Wang,

Department of Neurology, The First Affiliated Hospital of Anhui Medical University, Hefei, 230022,China;Collaborative Innovation Centre of Neuropsychiatric Disorders and Mental Health, Anhui Province, 230022,China;Department of Medical Psychology, Anhui Medical University, Hefei, 230022,China

Tel: +86-0551-62922418,

Fax:+86-021-64085875,

E-mail: xiecj2016@163.com.

Word count: 6288

For peer review only

Abstract

Objective: This study aimed to conduct a meta-analysis to explore and summarize the evidence regarding the association between obstructive sleep apnea (OSA) and the subsequent risk of vascular outcomes and all-cause mortality.

Methods: Electronic databases PubMed, Embase, and the Cochrane Library were searched to identify studies conducted through May 2016. Prospective cohort studies that reported effect estimates with 95% confidence intervals of major adverse cardiac events (MACEs), coronary heart disease (CHD), stroke, cardiac death, all-cause mortality, and heart failure for different levels versus the lowest level of OSA were included.

Results: A total of 16 cohort studies reporting data on 24,308 individuals were included. Of these, 11 studies reported healthy participants, and the remaining 5 studies reported participants with different diseases. Severe OSA was associated with an increased risk of MACEs (relative risk [RR]: 2.04; 95%CI: 1.56–2.66; $P < 0.001$), CHD (RR: 1.63; 95%CI: 1.18–2.26; $P = 0.003$), stroke (RR: 2.15; 95%CI: 1.42–3.24; $P < 0.001$), cardiac death (RR: 2.96; 95%CI: 1.45–6.01; $P = 0.003$), and all-cause mortality (RR: 1.54; 95%CI: 1.21–1.97; $P < 0.001$). Moderate OSA was also significantly associated with increased risk of MACEs (RR: 1.16; 95%CI: 1.01–1.33; $P = 0.034$) and CHD (RR: 1.38; 95%CI: 1.04–1.83; $P = 0.026$). No significant association was found between mild OSA and the risk of vascular outcomes or all-cause mortality ($P > 0.05$). Finally, no evidence of a factor-specific difference in

For peer review only

the risk ratio for MACEs among participants with different levels of OSA compared with those with the lowest level of OSA was found.

Conclusions: Severe and moderate OSAs were associated with an increased risk of vascular outcomes and all-cause mortality. This relationship might differ between genders. Therefore, further large-scale prospective studies are needed to verify this difference.

Key words: Meta-analysis; mortality, obstructive sleep apnea, vascular outcome

For peer review only

Article Summary:

Strengths and limitations of this study:

1. This was a meta-analysis to elucidate the association of obstructive sleep apnea (OSA) with fatal and nonfatal cardiovascular diseases, using a broad search strategy and predefined selection criteria and with no restriction of language or publication status.

2. The methodological quality of each study was assessed using the Newcastle-Ottawa Scale for prospective observational studies, and a meta-analysis, sensitivity analysis, subgroup analysis and bias assessment were also conducted.

3. Only prospective studies were included, eliminating selection and recall bias that could be of concern in retrospective case-control studies.

4. Summary relative risks were calculated to evaluate any potential difference between subsets according to the characteristics of the participants.

5. Different cutoff values for the apnea-hypopnea index might affect the relationship between OSA and vascular outcomes.

For peer review only

Introduction

Obstructive sleep apnea (OSA) affects 24% of middle-aged men and 9% of women in the United States, but daytime sleepiness was reported in 17% and 22% of these subjects, respectively [1]. OSA is an increasingly prevalent condition characterized by repetitive obstruction of the upper airway during sleep accompanied by episodic hypoxia, arousal, and sleep fragmentation [2]. Previous studies suggested that OSA was associated with an increased risk of glaucoma, diabetic kidney disease, and metabolic syndrome [3-5]. However, data on the association between OSA and the risk of subsequent vascular outcomes and mortality are both limited and inconclusive. Furthermore, whether these relationships differ according to the characteristics of patients with OSA also needs to be verified.

Several meta-analyses have illustrated that continuous positive airway pressure (CPAP) interventions aimed at OSA may reduce the risk of cardiovascular outcomes. Kim et al. [6] showed that CPAP treatment for OSA was associated with a lower incidence of stroke and cardiac events. Furthermore, Bratton et al. [7] indicated that use of both CPAP and mandibular advancement devices was associated with a reduction in the blood pressure among patients with OSA. Nadeem et al. [8] suggested that CPAP treatment for OSA seemed to improve dyslipidemia (decrease in total cholesterol and low-density lipoprotein, and increase in high-density lipoprotein), whereas it did not appear to affect the triglyceride levels. These studies demonstrated that patients with OSA who received interventions had a reduced risk of cardiovascular diseases. Therefore, clarifying the relationship between OSA and

For peer review only

vascular outcomes is particularly important as it has not been definitively determined. This study attempted to perform a large-scale examination of the available prospective studies to determine the association of OSA with the potential risk of vascular outcomes and all-cause mortality.

Methods

Data sources, search strategy, and selection criteria

This study was conducted and reported according to the Meta-analysis of Observational Studies in Epidemiology protocol (Checklist S1) [9].

Any prospective cohort study that examined the relationship between OSA and vascular outcomes or all-cause mortality was eligible for inclusion into this study, and no restrictions were placed on language or publication status (e.g., published, in press, or in progress). Electronic databases PubMed, Embase, and the Cochrane Library were searched for articles published through May 2016, using the terms “sleep apnea” OR “obstructive sleep apneas” AND (“cardiovascular disease” OR “stroke” OR “cardiac death” OR “mortality” OR “death” OR “CVD” OR “myocardial infarction” OR “coronary events”) AND “clinical trials” AND “human” as the search terms (Supplemental 1). Manual searches of reference lists were also conducted from all the relevant original and reviewed articles to identify additional eligible studies. The medical subject heading, methods, patient population, design, exposure, and outcome variables of these articles were used to identify the relevant studies.

For peer review only

The literature search was independently undertaken by two authors using a standardized approach. Any inconsistencies between these two authors were settled by the primary author until a consensus was reached. The study was eligible for inclusion if the following criteria were met: (1) the study had a prospective cohort design; (2) the study investigated the association between OSA and the risk of major adverse cardiac events (MACEs), coronary heart disease (CHD), stroke, cardiac death, all-cause mortality, and heart failure; and (3) the authors reported effect estimates [relative risk (RR), hazard ratio (HR), or odds ratio (OR)] and 95% confidence intervals (CIs) for comparisons of different levels of OSA versus lowest OSA level. All case-control studies were excluded because various confounding factors could bias the results.

Data collection and quality assessment

The data collected included the first author's name, publication year, country, sample size, mean age at baseline, percentage of male patients, body mass index (BMI), disease status, assessment of OSA, follow-up duration, effect estimate and its 95% CI, reported endpoints, and covariates in the fully adjusted model. For studies that reported several multivariable adjusted RRs, the effect estimate that was maximally adjusted for potential confounders was selected.

The Newcastle–Ottawa Scale (NOS), which is quite comprehensive and has been partially validated for evaluating the quality of observational studies in the meta-analysis, was used to evaluate the methodological quality [10]. The NOS is

For peer review only

based on the following three subscales: selection (4 items), comparability (1 item), and outcome (3 items). A "star system" (range, 0–9) was developed for assessment (Table 1). The data extraction and quality assessment were conducted independently by two authors. Information was examined and adjudicated independently by an additional author referring to the original studies.

Statistical analysis

The relationship between OSA and the risk of vascular outcomes or all-cause mortality based on the effect estimate (OR, RR, or HR) and its 95% CI was examined in each study. HR was considered to be equivalent to RR in cohort studies. Given the low incidence of vascular outcomes and all-cause mortality, ORs could be considered as accurate estimates of RRs [11]. A semi-parametric method was first used to evaluate the association of mild OSA [apnea–hypopnea index (AHI): 5–15], moderate OSA (AHI: 15–30) and severe OSA (AHI > 30) with the risk of vascular outcomes or all-cause mortality in order to analyze the trend between OSA levels and vascular outcomes or all-cause mortality risk [12]. For each individual study, each category of AHI was reclassified based on its calculated mid-point (for closed categories) or median (for open categories, assuming a normal distribution for AHI). The control category was composed of participants with the lowest AHI or normal participants in that study. Furthermore, when an individual study provided more than one median AHI level for classification among the three categories (i.e. mild, moderate or severe OSA), a fixed-effects model was used to calculate their summary RRs and 95% CIs to obtain effect estimates for each category [13]. If the study data were not broken down

For peer review only

by AHI but rather by oxygen desaturation index (ODI), classification into the OSA categories was carried out based on the judgment of the clinicians. A random-effects model was then used to calculate summary RRs and 95% CIs for mild, moderate, and severe OSA versus normal [14]. Finally, the ratio of RRs and the corresponding 95% CIs between subgroups were estimated using specific RRs and 95% CIs in each group based on the country, mean age, gender, BMI, disease status, and duration of the follow-up period [15].

Heterogeneity between studies was investigated using the Q statistic, and *P* values <0.10 was considered as indicative of significant heterogeneity [16 17]. Subgroup analyses were conducted for mild, moderate, and severe OSA and the risk of MACEs based on the country, mean age, gender, BMI, disease status, and duration of the follow-up period. A sensitivity analysis was also performed by removing each individual study from the meta-analysis [18]. Several methods were used to check for potential publication bias. Visual inspections of funnel plots for MACEs were conducted. The Egger [19] and Begg [20] tests were also used to statistically assess publication bias for MACEs. All reported *P* values were two sided, and *P* values <0.05 were regarded as statistically significant for all included studies. Statistical analyses were performed using the STATA software (version 12.0; Stata Corporation, TX, USA).

Results

For peer review only

Literature search

The results of the study-selection process are shown in Figure 1. An initial electronic search yielded 3282 articles, of which 3236 duplicates and irrelevant studies were excluded, and 46 potentially eligible studies were selected. After detailed evaluations, 16 prospective studies were selected for the final meta-analysis [21-36]. No new studies qualified for inclusion after a manual search of the reference lists of these studies. The general characteristics of the included studies are presented in Table 1.

Study characteristics

A total of 16 studies with 24,308 individuals qualified for this study. The follow-up period for participants was 2.9–18.0 years, while 73–10,149 individuals were included in each study. Eight studies were conducted in the United States, four in Spain, one in Sweden, one in Portugal, one in Hungary, and one in Canada. Furthermore, 11 studies reported healthy participants, and the remaining 5 studies reported participants with different diseases. The mean BMI ranged from 26.8 to 34.0 kg/m^2. Fourteen studies used polysomnography (PSG), and the remaining one study used limited PSG to assess the levels of OSA. The study quality was assessed using the NOS (Table 1). Overall, one study had a score of 9, six studies had a score of 8, seven studies had a score of 7, and the remaining two studies had a score of 6.

Table 1. Baseline characteristic of studies included in the systematic review and meta-analysis

Study	Country	Sample size	Mean age	Percentage male (%)	BMI	Disease status	Assessment OSA	AHI or ODI categories	Follow-up duration (year)	Reported outcomes	Adjusted factors	NOS score
Mooe et al. 2000 [21]	Sweden	408	59.1	58.4	27.0	CAD	Limited PSG	< 5; 5-10; 10-15; ≥ 15	5.1	CHD, stroke, all-cause mortality	Age, sex, BMI, hypertension, DM, LVF, and coronary intervention	7
Gottlieb et al. 2010 [22]	USA	4422	62.4	43.5	28.2	Healthy	PSG	< 5; 5-15; 15-30; ≥ 30	8.7	HF	Age, race, BMI, smoking, DM, SBP, DBP, TC, HDL-C, lipid-lowering	8

For peer review only

											medications, and antihypertensive medications	
Campos-Rodriguez et al. 2012 [23]	Spain	1116	56.1	0.0	36.6	Healthy	PSG	< 10; 10-29; ≥ 30	6.0	Cardiac death	Age, BMI, DM, hypertension, and previous CVD	8
Marin et al. 2005 [24]	Spain	1729	49.9	100	28.7	Healthy	PSG	5-30; ≥ 30	10.1	Cardiac death and CHD	Age, diagnostic group, presence of CVD, DM, hypertension, lipid disorders, smoking, alcohol, SBP DBP,	9

For peer review only

											blood glucose, TC, TG, and use of antihypertensive, lipid-lowering and antidiabetic drugs	
Young et al. 2008 [25]	USA	1522	48.0	55.0	28.6	Healthy	PSG	5-15; 15-30; ≥ 30	18.0	Cardiac death, all-cause mortality, and CHD	Age, age-squared, sex, BMI, and BMI squared	8
Redline et al. 2010 [26]	USA	5422	62.9	45.4	27.8	Healthy	PSG	Quartile I (0-4.05); Quartile II	8.7	Stroke	Age, BMI, race, smoking, SBP, DM, and antihypertensive	8

For peer review only

								(4.05-9.50); Quartile III (9.50-19.13); Quartile IV (>19.13)			medications	
Arzt et al. 2005 [27]	USA	1189	47.0	55.0	30.0	Healthy	PSG	<5; 5-20; ≥ 20	4.0	Stroke	Age, sex, and BMI	7
Punjabi et al. 2008	USA	6294	62.5	47.0	27.8	Healthy	PSG	Quartile I (0-8.50);	8.2	CHD, all-cause	Age, sex, race, BMI, SBP, DBP, smoking,	8

For peer review only

[28]								Quartile II (8.51-15.09); Quartile III (15.10-24.28); Quartile IV (>24.28)		mortality	prevalent hypertension, DM, and CVD	
Shah et al. 2010 [29]	USA	1436	59.7	69.4	32.9	Healthy	PSG	<5; 5-14; 15-29; ≥	2.9	CHD, cardiac death	Age, race, sex, smoking, alcohol,	7

For peer review only

								30			BMI, AF, DM, hypertension, and hyperlipidemia	
Yaggi et al. 2005 [30]	USA	1022	60.2	71.3	32.8	Healthy	PSG	⩽3; 4-12; 13-36; ≥ 36	3.4	Stroke and all-cause mortality	Age, sex, race, smoking, alcohol, BMI, DM, hyperlipidemia, AF, and hypertension	8
Martı´nez-Garcı´a et al. 2009 [31]	Spain	166	73.3	59.0	28.1	Ischemic Stroke	PSG	0-9; 10-19; ≥ 20	5.0	All-cause mortality	Age, sex, Barthel index, AHI, and CPAP treatment groups, previous stroke or	7

For peer review only

For peer review only - http://bmjopen.bmj.com/site/about/guidelines.xhtml

											TIA, diabetes, hypercholesterolemia, BMI, smoking, arterial hypertension, atrial fibrillation, significant carotid stenosis, and fibrinogen levels	
Munoz et al. 2006 [32]	Spain	1034	79.8	57.0	26.8	Healthy	PSG	<30; ≥ 30	6.0	Stroke	Sex	7
Leão et al.	Portugal	73	62.4	75.0	27.6	Acute	PSG	5-15;	6.3	CHD	Sex	7

For peer review only

2016 [33]						coronary syndrome		15-30; ≥ 30				
Fornadi et al. 2014 [34]	Hungary	100	51.0	56.8	26.8	Kidney transplant recipients	PSG	5-15; 15-30; ≥ 30	6.3	All-cause mortality	Unadjusted	6
Kendzerska et al. 2014 [35]	Canada	10149	49.9	62.0	30.1	Healthy	PSG	< 5; 5-15; 15-30; ≥ 30	5.7	All-cause mortality	Traditional CV risk factors	7

For peer review only

Won et al. 2013 [36]	USA	281	65.0	98.0	34.0	Ischemic heart disease and myocardial injury	PSG	5-30; ≥ 30	4.1	All-cause mortality	NA	6

AF, atrial fibrillation; AHI: apnea–hypopnea index; BMI, body mass index; CAD, coronary artery disease; CHD, coronary heart disease; CPAP, continuous positive airway pressure; CV, cardiovascular; CVD, cardiovascular disease; DBP, diastolic blood pressure; DM, diabetes mellitus; HDL-C, high-density lipoprotein cholesterol; HF, heart failure; LVF, left ventricular function; NA, not applicable; ODI: oxygen desaturation index; OSA, obstructive sleep apnea; PSG, polysomnography; SBP, systolic blood pressure; TC, total cholesterol; TG, triglyceride; TIA, transient ischemic attack.

For peer review only

OSA and MACE risk

The summary RRs showed that mild OSA was not associated with MACEs (RR: 0.98; 95% CI: 0.87–1.11; *P* = 0.741; Fig. 2 and Table 2). Furthermore, the pooled analysis results for moderate and severe OSA indicated that they had a harmful effect on the risk of MACEs (moderate: RR, 1.16; 95% CI, 1.01–1.33; *P* = 0.034; Fig. 3 and Table 2; severe: RR, 2.04; 95% CI, 1.56–2.66; *P* < 0.001; Fig. 4 and Table 2). A subgroup analysis for MACEs was conducted to minimize heterogeneity among the included studies and evaluate the relationship between OSA and MACEs in specific subpopulations (Table 3). Overall, participants with moderate OSA were associated with an increased risk of MACEs if individuals did not have other diseases (RR: 1.16; 95% CI: 1.01–1.33; *P* = 0.034). Furthermore, no significant association was found between severe OSA and MACEs if the study included only women (RR: 1.98; 95% CI: 0.64–6.06; *P* = 0.234); in other subsets, severe OSA was associated with an increased risk of MACEs (Table 3). Finally, no evidence of a factor-specific difference was found in the RR for MACEs among participants with OSA compared with controls (Table 3).

Table 2. Summary of the relative risks of all outcomes evaluated

Outcomes	Mild OSA (RR with 95% CI)	*P* value for mild OSA	Moderate OSA (RR with 95% CI)	*P* value for moderate OSA	Severe OSA (RR with 95% CI)	*P* value for severe OSA
MACEs	0.98 (0.87–1.11)	0.741	1.16 (1.01–1.33)	0.034	2.04 (1.56–2.66)	<0.001

For peer review only

CHD	1.25 (0.95–1.66)	0.117	1.38 (1.04–1.83)	0.026	1.63 (1.18–2.26)	0.003
Stroke	1.29 (0.69–2.41)	0.424	1.35 (0.82–2.23)	0.245	2.15 (1.42–3.24)	<0.001
Cardiac death	1.80 (0.68–4.76)	0.236	1.11 (0.53–2.35)	0.781	2.96 (1.45–6.01)	0.003
All-cause mortality	1.26 (0.77–2.07)	0.354	1.04 (0.60–1.79)	0.895	1.54 (1.21–1.97)	<0.001
Heart failure	1.02 (0.78–1.34)	0.868	1.07 (0.74–1.54)	0.719	1.44 (0.94–2.21)	0.097

CHD, Coronary heart disease; CI: confidence interval; MACE, major cardiovascular event; OSA, obstructive sleep apnea; RR: relative risk.

Table 3. Subgroup analyses for MACEs

Variable	Subgroup	Mild OSA (RR with 95% CI)	*P* value for mild OSA	Moderate OSA (RR with 95% CI)	*P* value for moderate OSA	Severe OSA (RR with 95% CI)	*P* value for severe OSA
Country	USA	1.00 (0.85–1.17)	0.977	1.14 (0.99–1.32)	0.064	**1.90 (1.35–2.67)**	**<0.001**
	Other	1.02 (0.19–5.52)	0.982	1.44 (0.83–2.50)	0.198	**2.35 (1.52–3.65)**	**<0.001**
	USA vs other	0.98 (0.18–5.32)*	0.982	0.79 (0.45–1.40)*	0.422	0.81 (0.46–1.41)*	0.453
Mean age	≥60	0.96 (0.86–1.08)	0.540	1.13 (0.97–1.33)	0.117	**1.78 (1.23–2.57)**	**0.002**
	<60	1.40 (0.73–2.70)	0.315	1.51 (0.94–2.41)	0.086	**2.31 (1.64–3.24)**	**<0.001**
	≥60 vs <60	0.69 (0.35–1.33)*	0.265	0.75 (0.46–1.23)*	0.252	0.77 (0.47–1.27)*	0.309

For peer review only

Gender	Male	0.92 (0.73–1.15)	0.455	1.10 (0.85–1.42)	0.449	**1.81 (1.14–2.89)**	**0.012**
	Female	1.97 (0.47–8.25)	0.353	1.36 (0.67–2.76)	0.399	1.98 (0.64–6.06)	0.234
	Male vs female	0.47 (0.11–1.99)*	0.304	0.81 (0.38–1.72)*	0.581	0.91 (0.27–3.08)*	0.885
BMI	≥30	1.75 (0.88–3.49)	0.111	1.70 (0.94–3.07)	0.079	**2.72 (1.80–4.10)**	**<0.001**
	<30	0.96 (0.86–1.07)	0.449	1.14 (0.99–1.31)	0.078	**1.80 (1.36–2.38)**	**<0.001**
	≥30 vs <30	1.82 (0.91–3.66)*	0.092	1.49 (0.81–2.74)*	0.198	1.51 (0.92–2.49)*	0.104
Disease statues	Healthy	1.00 (0.85–1.17)	0.977	**1.16 (1.01–1.33)**	**0.034**	**2.12 (1.53–2.94)**	**<0.001**
	Other	1.02 (0.19–5.52)	0.982	–	-	**1.96 (1.01–3.81)**	**0.047**
	Healthy vs Other	0.98 (0.18–5.32)*	0.982	–	-	1.08 (0.52–2.27)*	0.835
Follow-up duration	≥6	0.96 (0.86–1.07)	0.449	1.14 (0.99–1.31)	0.064	**2.06 (1.43–2.95)**	**<0.001**
	<6	1.75 (0.88–3.49)	0.111	1.74 (0.87–3.49)	0.120	**2.10 (1.39–3.17)**	**<0.001**
	≥6 vs <6	0.55 (0.27–1.10)*	0.092	0.66 (0.32–1.33)*	0.242	0.98 (0.57–1.70)*	0.945

BMI, body mass index; CI: confidence interval; OSA, obstructive sleep apnea; RR: relative risk. * Reported as ratio of RR and 95% CI.

OSA and CHD risk

The pooled data of meta-analysis showed that mild OSA was not associated with the risk of CHD (RR: 1.25; 95% CI: 0.95–1.66; *P* = 0.117; Table 2 and Supplemental 2), whereas moderate OSA (RR: 1.38; 95% CI: 1.04–1.83; *P* = 0.026; Table 2 and Supplemental 2) and severe OSA (RR: 1.63; 95% CI: 1.18–2.26; *P* = 0.003; Table 2

For peer review only

and Supplemental 2) were associated with a significantly increased risk of CHD. Stratified analyses according to gender were conducted for different levels of OSA versus normal group, and it was found that patients with severe OSA had significantly increased the risk of CHD in men (RR: 1.65; 95% CI: 1.06–2.57; *P* = 0.027). No other significant differences were detected (Table 4).

Table 4. Gender difference for other outcomes

Outcome	Subgroup	Mild OSA (RR with 95% CI)	*P* value for mild OSA	Moderate OSA (RR with 95% CI)	*P* value for moderate OSA	Severe OSA (RR with 95% CI)	*P* value for severe OSA
CHD	Men	0.93 (0.72–1.21)	0.596	1.09 (0.80–1.48)	0.582	**1.65 (1.06–2.57)**	**0.027**
	Women	1.92 (0.43–8.64)	0.394	1.51 (0.38–5.97)	0.559	1.10 (0.12–9.87)	0.933
	Men vs women	0.48 (0.11–2.22)*	0.351	0.72 (0.18–2.96)*	0.651	1.50 (0.16–14.22)*	0.724
Stroke	Men	1.86 (0.67–5.14)	0.232	1.86 (0.70–4.95)	0.214	**2.86 (1.10–7.41)**	**0.031**
	Women	1.34 (0.76–2.36)	0.311	1.20 (0.67–2.15)	0.542	1.21 (0.65–2.25)	0.546
	Men vs women	1.39 (0.43–4.45)*	0.581	1.55 (0.50–4.84)*	0.451	2.36 (0.76–7.38)*	0.138
Cardiac death	Men	–	–	1.15 (0.41–3.23)	0.791	**2.87 (1.13–7.27)**	**0.026**
	Women	–	–	0.94 (0.19–4.61)	0.939	3.71 (0.41–33.87)	0.245
	Men vs women	–	–	1.22 (0.18–8.17)*	0.935	0.77 (0.07–8.49)*	0.834

For peer review only

All-cause mortality	Men	–	–	–	–	**1.72 (1.22–2.43)**	**0.002**
	Women	–	–	–	–	**3.50 (1.23–9.97)**	**0.019**
	Men vs women	–	–	–	–	0.49 (0.16–1.48)*	0.206
Heart failure	Men	0.88 (0.57–1.35)	0.561	1.13 (0.68–1.88)	0.639	1.58 (0.93–2.67)	0.088
	Women	1.13 (0.80–1.60)	0.493	1.01 (0.60–1.70)	0.970	1.19 (0.56–2.52)	0.650
	Men vs women	0.78 (0.45–1.35)*	0.376	1.12 (0.54–2.32)*	0.762	1.33 (0.53–3.33)*	0.545

CHD, coronary heart disease; OSA, obstructive sleep apnea. * Reported as ratio of RR and 95% CI.

OSA and stroke risk

Pooled analysis results indicated no association between mild OSA (RR: 1.29; 95% CI: 0.69–2.41; $P = 0.424$; Table 2 and Supplemental 2) and moderate OSA (RR: 1.35; 95% CI: 0.82–2.23; $P = 0.245$; Table 2 and Supplemental 2) and stroke, whereas severe OSA was associated with an increased risk of stroke (RR: 2.15; 95% CI: 1.42–3.24; $P < 0.001$; Table 2 and Supplemental 2). Subgroup analysis on the basis of gender indicated that severe OSA had a harmful effect on the risk of stroke in men (RR: 2.86; 95% CI: 1.10–7.41; $P = 0.031$; Table 4).

OSA and cardiac death risk

The summary RRs showed that mild OSA (RR: 1.80; 95% CI: 0.68–4.76; $P = 0.236$; Table 2 and Supplemental 2) and moderate OSA (RR: 1.11; 95% CI: 0.53–2.35; $P = 0.781$; Table 2 and Supplemental 2) were not associated with cardiac death risk,

For peer review only

whereas severe OSA significantly increased the risk of cardiac death (RR: 2.96; 95% CI: 1.45–6.01; P = 0.003; Table 2 and Supplemental 2). Subgroup analysis showed that severe OSA was associated with an increased risk of cardiac death in men (RR: 2.87; 95% CI: 1.13–7.27; P = 0.026; Table 4).

OSA and all-cause mortality risk

No significant association was found between mild OSA (RR: 1.26; 95% CI: 0.77–2.07; P = 0.354; Table 2 and Supplemental 2), moderate OSA (RR: 1.04; 95% CI: 0.60–1.79; P = 0.895; Table 2 and Supplemental 2), and all-cause mortality risk. However, severe OSA had a harmful impact on the all-cause mortality (RR: 1.54; 95% CI: 1.21–1.97; P < 0.001; Table 2 and Supplemental 2). Stratified analysis suggested that severe OSA increased the risk of all-cause mortality in men (RR: 1.72; 95% CI: 1.22–2.43; P = 0.002) and women (RR: 3.50; 95% CI: 1.23–9.97; P = 0.019; Table 4).

OSA and heart failure risk

The summary results indicated no significant differences between mild OSA (RR: 1.02; 95% CI: 0.78–1.34; P = 0.868), moderate OSA (RR: 1.07; 95% CI: 0.74–1.54; P = 0.719), and severe OSA (RR: 1.44; 95% CI: 0.94–2.21; P = 0.097), and the risk of heart failure (Table 2 and Supplemental 2). Subgroup analysis reported similar results compared with the overall analysis (Table 4).

Publication bias

Review of the funnel plots could not rule out the potential publication bias for

For peer review only

MACEs (Fig. 5). The Egger and Begg test results showed no evidence of publication bias for MACEs of mild OSA (*P* value for Egger: 0.132; *P* value for Begg: 0.221) and moderate OSA (*P* value for Egger: 0.052; *P* value for Begg: 0.452). Although the Begg test showed no evidence of publication bias for MACEs of severe OSA (P = 0.118), the Egger test showed potential evidence of publication bias for MACEs of severe OSA ($P < 0.001$). The conclusion did not change after adjustment for publication bias using the trim-and-fill method [37].

Discussion

The present study was based on prospective cohort studies and explored all possible correlations between OSA and the outcomes of MACEs, CHD, stroke, cardiac death, all-cause mortality, and heart failure. This large quantitative study included 24,308 individuals from 16 prospective cohort studies with a broad range of populations. The findings from the present meta-analysis suggested that mild OSA had no significant impact on the risk of vascular outcomes and all-cause mortality, moderate OSA was associated with an increased risk of MACEs and CHD, and severe OSA had a harmful effect on the risk of MACEs, CHD, stroke, cardiac death, and all-cause mortality.

A previous meta-analysis suggested that OSA was associated with stroke, but its relationship with ischemic heart disease and cardiovascular mortality needs further research [38]. However, this study could not illustrate the impact of different levels of OSA on the risk of serious cardiovascular outcomes. Further, Dong et al. suggested that moderate-to-severe OSA significantly increased the risk of cardiovascular

diseases, in particular, the risk of stroke [39]. Similarly, Ge et al. indicated that severe OSA is a strong independent predictor of cardiovascular and all-cause mortality. CPAP treatment was associated with decreased cardiovascular mortality [40]. However, these two studies could not evaluate the association of OSA with the risk of vascular outcomes and all-cause mortality in specific subpopulations. In addition, Wang et al. suggested that severe OSA significantly increased the risk of CHD and stroke, and all-cause mortality. A positive association with CHD was observed for moderate OSA but not for mild OSA [41]. However, whether this relationship differs according to the characteristics of participants remains unclear. Finally, Xie et al. conducted a meta-analysis to evaluate the relationship between OSA and recurrent vascular events and all-cause mortality [42]. However, they just compared the highest AHI versus lowest AHI, whereas the degree of OSA and subsequent adverse outcomes were not available. Therefore, a comprehensive meta-analysis of these prospective cohort studies was performed to evaluate any possible correlates between OSA and vascular outcomes.

No significant difference was observed between mild OSA and the risk of vascular outcomes. However, several studies included in this study reported inconsistent results. Young et al. suggested that mild OSA significantly increased the risk of CHD by 92% [25], whereas Punjabi et al. indicated that mild OSA might have a harmful effect on the risk of CHD [28]. This might be because these two studies used healthy individuals as controls, which may make them more susceptible to acquired significant conclusion. Furthermore, most of these studies did not take into account

For peer review only

potential confounders for the risk of cardiovascular disease. Moderate-to-severe OSA might play an important role in the risk of vascular outcomes. Shah et al. concluded that OSA increased the risk of coronary events or death from cardiovascular causes [29]. Nearly all included studies reported adverse outcomes for severe OSA. Finally, Previous studies indicated that OSA was a cause of diabetes, which was an independent risk factor for MACEs [43].

Subgroup analyses reported similar conclusions. Gender might have an impact on the relationship between OSA and CHD, stroke, or cardiac death, although the sex difference was not statistically significant. The possible reasons could be the lower prevalence of severe OSA in women and the later age of onset of OSA in women than in men. Furthermore, OSA in women always occurred after menopause. Physiological response to OSA is another reason for this nonsignificant difference. Finally, these conclusions might be unreliable because smaller cohorts were included in each subset. Therefore, further large-scale studies were needed to verify this difference. Therefore, a relative result was given, and a synthetic and comprehensive review was provided.

No significant difference was found between mild or moderate OSA and all-cause mortality, while severe OSA was associated with an increased risk of all-cause mortality. Further, these significant associations were also observed in men and women separately. Although the effect estimate in women was larger than that in men, no gender difference was found in the relationship between OSA and all-cause mortality. This might be because the number of studies that reported the relationship between severe OSA and all-cause mortality was smaller than expected, and a broad

For peer review only

95% CI was acquired. Therefore, the association of severe OSA with all-cause mortality in women was variable and should be verified in future large-scale prospective studies.

Three strengths of this study should be highlighted. First, only prospective studies were included, which eliminated selection and recall bias, and could be of concern in retrospective case–control studies. Second, the large sample size allowed us to quantitatively assess the association of OSA with the risk of vascular outcomes and mortality, and thus the findings were potentially more robust than those of any individual study. Third, the summary RRs were calculated to evaluate any potential difference between subsets according to the characteristics of participants.

The limitations of this study were as follows: (1) the adjusted models were different across the included studies, and these factors might have played an important role in developing vascular outcomes; (2) in a meta-analysis of published studies, publication bias was an inevitable problem; and (3) the analysis used pooled data (individual data were not available), which restricted performing a more detailed relevant analysis and obtaining more comprehensive results.

The results of this study suggested that moderate-to-severe OSA might play an important role in the risk of vascular outcomes, especially for men. Future studies should focus on specific populations to analyze the gender difference to study the association between OSA and vascular outcomes.

For peer review only

Author Contributions

Chengjuan Xie carried out the studies, participated in collecting data, and drafted the manuscript. Ruolin Zhu performed the statistical analysis and participated in its design. Yanghua Tian and Kai Wang helped to draft the manuscript. All authors read and approved the final manuscript.

Conflict of interests: All authors declare no conflict of interest.

Funding statement: This research was supported by the National Basic Research Program of China (nos. 973 Program 2015CB856405 and 2012CB720704) and the National Natural Science Foundation of China (nos. 31571149, 91432301, 81301176, and 81171273).

Data sharing statement: No additional data available.

For peer review only

References

1 Young T, Palta M, Dempsey J, et al. The occurrence of sleep-disordered breathing among middle-aged adults. *N Engl J Med* 1993;**328**(17):1230-5 doi: 10.1056/NEJM199304293281704published Online First: Epub Date]|.

2 Young T, Peppard PE, Taheri S. Excess weight and sleep-disordered breathing. *J Appl Physiol (1985)* 2005;**99**(4):1592-9 doi: 10.1152/japplphysiol.00587.2005published Online First: Epub Date]|.

3 Liu S, Lin Y, Liu X. Meta-Analysis of Association of Obstructive Sleep Apnea With Glaucoma. *J Glaucoma* 2016;**25**(1):1-7 doi: 10.1097/IJG.0000000000000357published Online First: Epub Date]|.

4 Leong WB, Jadhakhan F, Taheri S, et al. The Association between Obstructive Sleep Apnea on Diabetic Kidney Disease: A Systematic Review and Meta-Analysis. *Sleep* 2016;**39**(2):301-8 doi: 10.5665/sleep.5432published Online First: Epub Date]|.

5 Xu S, Wan Y, Xu M, et al. The association between obstructive sleep apnea and metabolic syndrome: a systematic review and meta-analysis. *BMC Pulm Med* 2015;**15**:105 doi: 10.1186/s12890-015-0102-3published Online First: Epub Date]|.

6 Kim Y, Koo YS, Lee HY, et al. Can Continuous Positive Airway Pressure Reduce the Risk of Stroke in Obstructive Sleep Apnea Patients? A Systematic Review and Meta-Analysis. *PLoS One* 2016;**11**(1):e0146317 doi: 10.1371/journal.pone.0146317published Online First: Epub Date]|.

For peer review only

7 Bratton DJ, Gaisl T, Wons AM, et al. CPAP vs Mandibular Advancement Devices and Blood Pressure in Patients With Obstructive Sleep Apnea: A Systematic Review and Meta-analysis. *JAMA* 2015;**314**(21):2280-93 doi: 10.1001/jama.2015.16303published Online First: Epub Date]|.

8 Nadeem R, Singh M, Nida M, et al. Effect of CPAP treatment for obstructive sleep apnea hypopnea syndrome on lipid profile: a meta-regression analysis. *J Clin Sleep Med* 2014;**10**(12):1295-302 doi: 10.5664/jcsm.4282published Online First: Epub Date]|.

9 Stroup DF, Berlin JA, Morton SC, et al. Meta-analysis of observational studies in epidemiology: a proposal for reporting. Meta-analysis Of Observational Studies in Epidemiology (MOOSE) group. *JAMA* 2000;**283**(15):2008-12

10 Wells G, Shea B, O'Connell D. The Newcastle-Ottawa Scale (NOS) for assessing the quality of nonrandomised studies in meta-analyses. *Ottawa Hospital Research Institute* 2009

11 Zheng YL, Lian F, Shi Q, et al. Alcohol intake and associated risk of major cardiovascular outcomes in women compared with men: a systematic review and meta-analysis of prospective observational studies. *BMC Public Health* 2015;**15**:773. doi: 10.1186/s12889-015-2081-y.

12 Bradley TD, Floras JS. Obstructive sleep apnoea and its cardiovascular consequences. *Lancet* 2009;**373**(9657):82-93 doi: 10.1016/S0140-6736(08)61622-0published Online First: Epub Date]|.

13 DerSimonian R, Laird N. Meta-analysis in clinical trials. *Control Clin Trials*

For peer review only

1986;**7**(3):177-88

14 Ades AE, Lu G, Higgins JP. The interpretation of random-effects meta-analysis in decision models. *Med Decis Making* 2005;**25**(6):646-54 doi: 10.1177/0272989X05282643published Online First: Epub Date]|.

15 Huxley RR, Woodward M. Cigarette smoking as a risk factor for coronary heart disease in women compared with men: a systematic review and meta-analysis of prospective cohort studies. *Lancet* 2011;**378**(9799):1297-305 doi: 10.1016/S0140-6736(11)60781-2published Online First: Epub Date]|.

16 Deeks JJ, Higgins JP, Altman DG. *Analysing Data and Undertaking Meta‐Analyses*: John Wiley & Sons, Ltd, 2008.

17 Higgins JP, Thompson SG, Deeks JJ, et al. Measuring inconsistency in meta-analyses. *BMJ* 2003;**327**(7414):557-60 doi: 10.1136/bmj.327.7414.557published Online First: Epub Date]|.

18 Altman DG, Bland JM. Interaction revisited: the difference between two estimates. *BMJ* 2003;**326**(7382):219

19 Egger M, Davey Smith G, Schneider M, et al. Bias in meta-analysis detected by a simple, graphical test. *BMJ* 1997;**315**(7109):629-34

20 Begg CB, Mazumdar M. Operating characteristics of a rank correlation test for publication bias. *Biometrics* 1994;**50**(4):1088-101

21 Mooe T, Franklin KA, Wiklund U, et al. Sleep-disordered breathing and myocardial ischemia in patients with coronary artery disease. *Chest* 2000;**117**(6):1597-602

For peer review only

22 Gottlieb DJ, Yenokyan G, Newman AB, et al. Prospective study of obstructive sleep apnea and incident coronary heart disease and heart failure: the sleep heart health study. *Circulation* 2010;**122**(4):352-60 doi: 10.1161/CIRCULATIONAHA.109.901801published Online First: Epub Date]|.

23 Campos-Rodriguez F, Martinez-Garcia MA, de la Cruz-Moron I, et al. Cardiovascular mortality in women with obstructive sleep apnea with or without continuous positive airway pressure treatment: a cohort study. *Ann Intern Med* 2012;**156**(2):115-22 doi: 10.7326/0003-4819-156-2-201201170-00006published Online First: Epub Date]|.

24 Marin JM, Carrizo SJ, Vicente E, et al. Long-term cardiovascular outcomes in men with obstructive sleep apnoea-hypopnoea with or without treatment with continuous positive airway pressure: an observational study. *Lancet* 2005;**365**(9464):1046-53 doi: 10.1016/S0140-6736(05)71141-7published Online First: Epub Date]|.

25 Young T, Finn L, Peppard PE, et al. Sleep disordered breathing and mortality: eighteen-year follow-up of the Wisconsin sleep cohort. *Sleep* 2008;**31**(8):1071-8

26 Redline S, Yenokyan G, Gottlieb DJ, et al. Obstructive sleep apnea-hypopnea and incident stroke: the sleep heart health study. *Am J Respir Crit Care Med* 2010;**182**(2):269-77 doi: 10.1164/rccm.200911-1746OCpublished Online

For peer review only

First: Epub Date]|.

27 Arzt M, Young T, Finn L, et al. Association of sleep-disordered breathing and the occurrence of stroke. *Am J Respir Crit Care Med* 2005;**172**(11):1447-51 doi: 10.1164/rccm.200505-702OCpublished Online First: Epub Date]|.

28 Punjabi NM, Newman AB, Young TB, et al. Sleep-disordered breathing and cardiovascular disease: an outcome-based definition of hypopneas. *Am J Respir Crit Care Med* 2008;**177**(10):1150-5 doi: 10.1164/rccm.200712-1884OCpublished Online First: Epub Date]|.

29 Shah NA, Yaggi HK, Concato J, et al. Obstructive sleep apnea as a risk factor for coronary events or cardiovascular death. *Sleep Breath* 2010;**14**(2):131-6 doi: 10.1007/s11325-009-0298-7published Online First: Epub Date]|.

30 Yaggi HK, Concato J, Kernan WN, et al. Obstructive sleep apnea as a risk factor for stroke and death. *N Engl J Med* 2005;**353**(19):2034-41 doi: 10.1056/NEJMoa043104published Online First: Epub Date]|.

31 Martinez-Garcia MA, Soler-Cataluna JJ, Ejarque-Martinez L, et al. Continuous positive airway pressure treatment reduces mortality in patients with ischemic stroke and obstructive sleep apnea: a 5-year follow-up study. *Am J Respir Crit Care Med* 2009;**180**(1):36-41 doi: 10.1164/rccm.200808-1341OCpublished Online First: Epub Date]|.

32 Munoz R, Duran-Cantolla J, Martinez-Vila E, et al. Severe sleep apnea and risk of ischemic stroke in the elderly. *Stroke* 2006;**37**(9):2317-21 doi: 10.1161/01.STR.0000236560.15735.0fpublished Online First: Epub Date]|.

For peer review only

33 Leao S, Conde B, Fontes P, et al. Effect of Obstructive Sleep Apnea in Acute Coronary Syndrome. *Am J Cardiol* 2016;**117**(7):1084-7 doi: 10.1016/j.amjcard.2015.12.053published Online First: Epub Date]|.

34 Fornadi K, Ronai KZ, Turanyi CZ, et al. Sleep apnea is not associated with worse outcomes in kidney transplant recipients. *Sci Rep* 2014;**4**:6987 doi: 10.1038/srep06987published Online First: Epub Date]|.

35 Kendzerska T, Gershon AS, Hawker G, et al. Obstructive sleep apnea and risk of cardiovascular events and all-cause mortality: a decade-long historical cohort study. *PLoS Med* 2014;**11**(2):e1001599 doi: 10.1371/journal.pmed.1001599published Online First: Epub Date]|.

36 Won CH, Chun HJ, Chandra SM, et al. Severe obstructive sleep apnea increases mortality in patients with ischemic heart disease and myocardial injury. *Sleep Breath* 2013;**17**(1):85-91 doi: 10.1007/s11325-012-0653-ypublished Online First: Epub Date]|.

37 Duval S, Tweedie R. A Nonparametric "Trim and Fill" Method of Accounting for Publication Bias in Meta-Analysis. *Journal of the American Statistical Association* 2000;**95**(449):89-98

38 Loke YK, Brown JW, Kwok CS, et al. Association of obstructive sleep apnea with risk of serious cardiovascular events: a systematic review and meta-analysis. *Circ Cardiovasc Qual Outcomes* 2012;**5**(5):720-8 doi: 10.1161/CIRCOUTCOMES.111.964783published Online First: Epub Date]|.

39 Dong JY, Zhang YH, Qin LQ. Obstructive sleep apnea and cardiovascular risk:

For peer review only

meta-analysis of prospective cohort studies. *Atherosclerosis* 2013;**229**(2):489-95 doi: 10.1016/j.atherosclerosis.2013.04.026published Online First: Epub Date]|.

40 Ge X, Han F, Huang Y, et al. Is obstructive sleep apnea associated with cardiovascular and all-cause mortality? *PLoS One* 2013;**8**(7):e69432 doi: 10.1371/journal.pone.0069432published Online First: Epub Date]|.

41 Wang X, Ouyang Y, Wang Z, et al. Obstructive sleep apnea and risk of cardiovascular disease and all-cause mortality: a meta-analysis of prospective cohort studies. *Int J Cardiol* 2013;**169**(3):207-14 doi: 10.1016/j.ijcard.2013.08.088published Online First: Epub Date]|.

42 Xie W, Zheng F, Song X. Obstructive sleep apnea and serious adverse outcomes in patients with cardiovascular or cerebrovascular disease: a PRISMA-compliant systematic review and meta-analysis. *Medicine (Baltimore)* 2014;**93**(29):e336. doi: 10.1097/MD.0000000000000336.

43 Wang X, Bi Y, Zhang Q, et al. Obstructive sleep apnoea and the risk of type 2 diabetes: a meta-analysis of prospective cohort studies. *Respirology* 2013;**18**(1):140-6. doi: 10.1111/j.1440-1843.2012.02267.x.

For peer review only

Figure legends:

Figure 1. Study-selection process.

Figure 2. Association between mild OSA and MACEs.

Figure 3. Association between moderate OSA and MACEs.

Figure 4. Association between severe OSA and MACEs.

Figure 5. Funnel plots.

Supplemental legends:

Checklist S1. MOOSE Checklist.

For peer review only

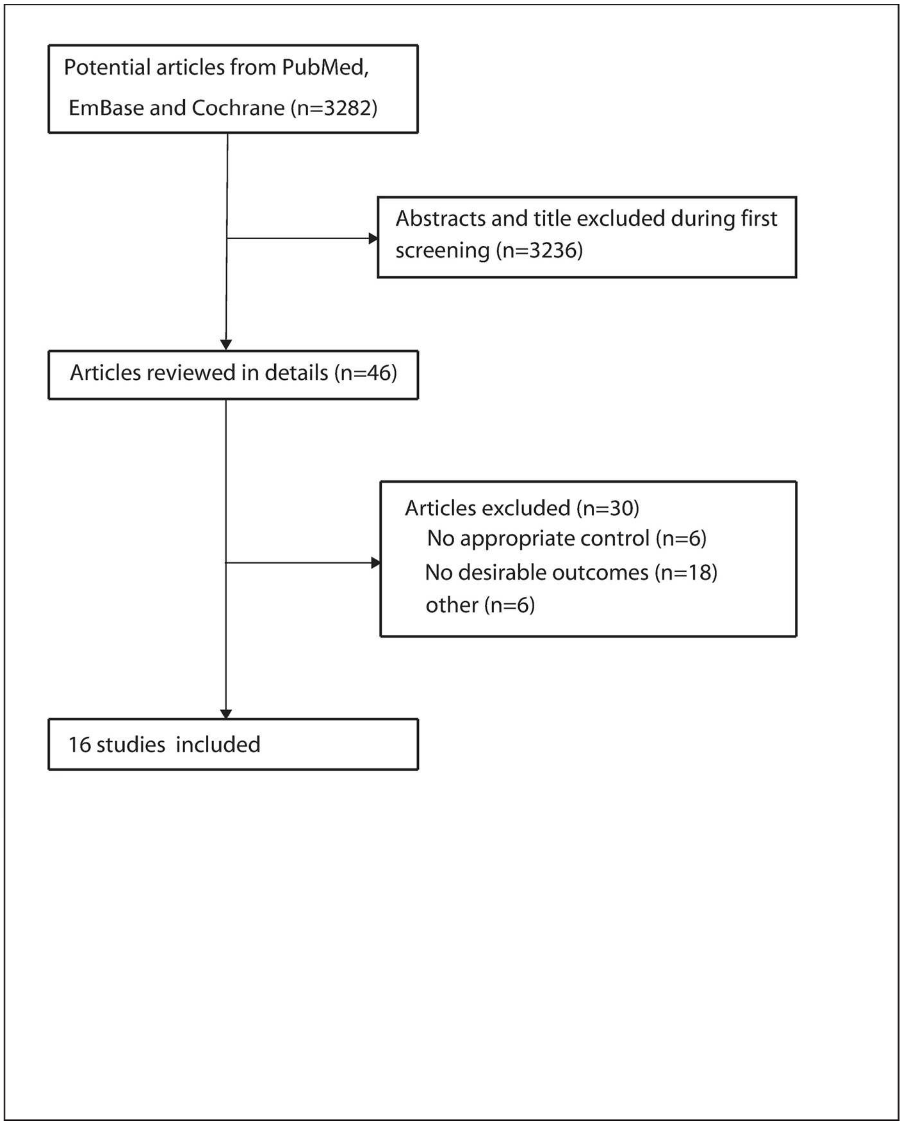

168x188mm (300 x 300 DPI)

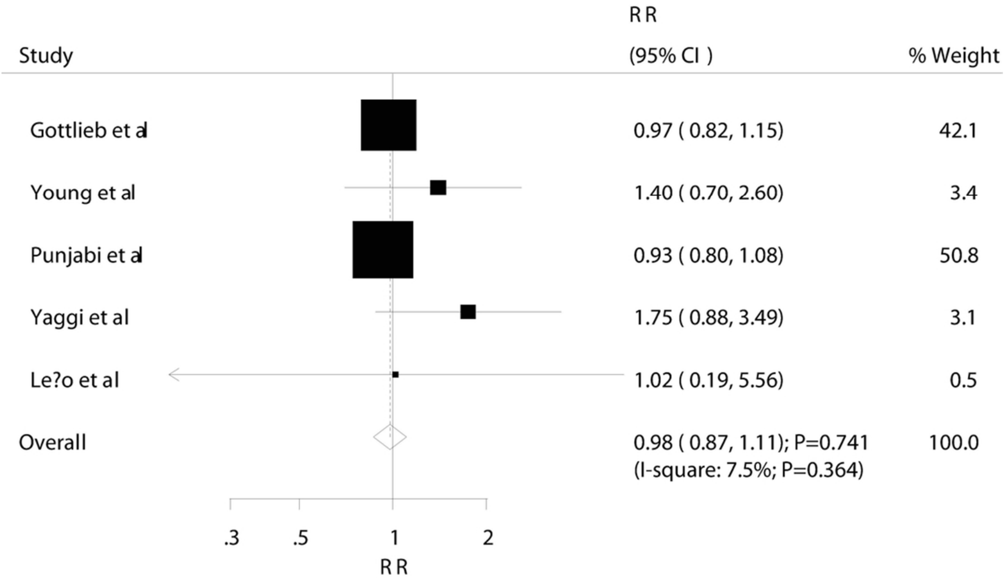

94x54mm (300 x 300 DPI)

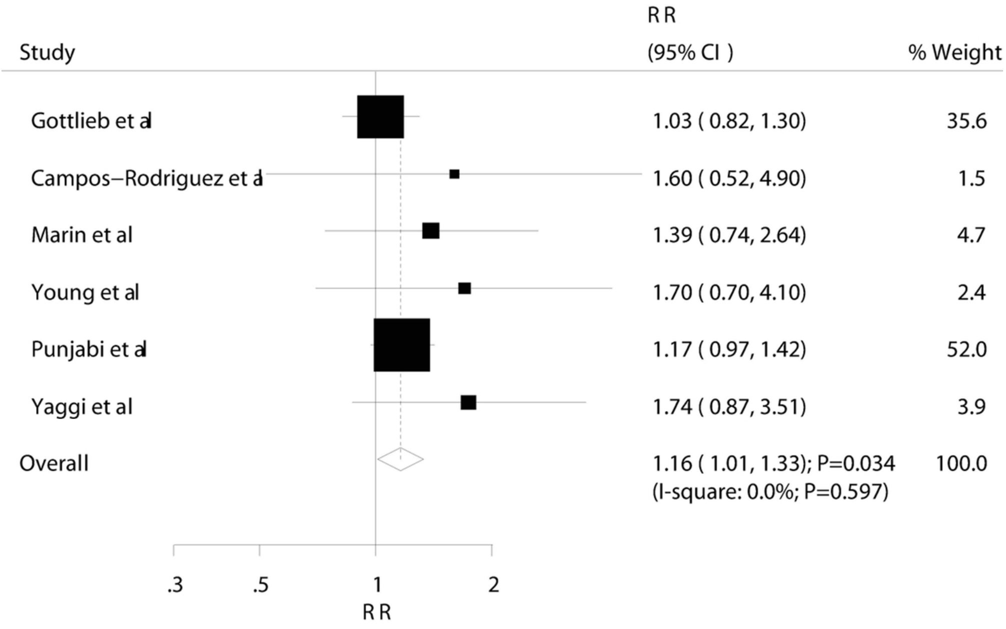

98x61mm (300 x 300 DPI)

review only

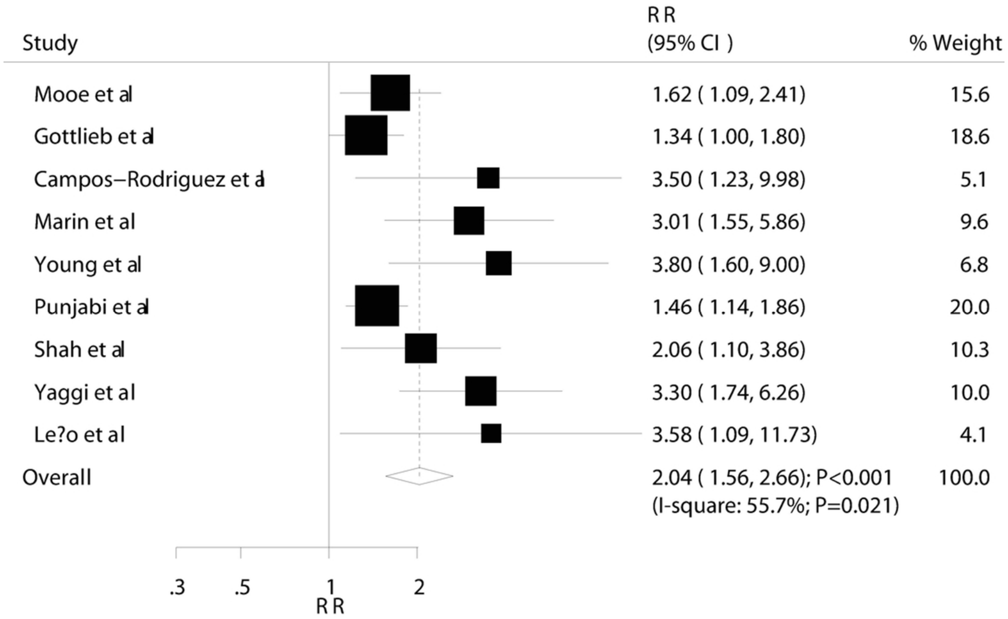

98x60mm (300 x 300 DPI)

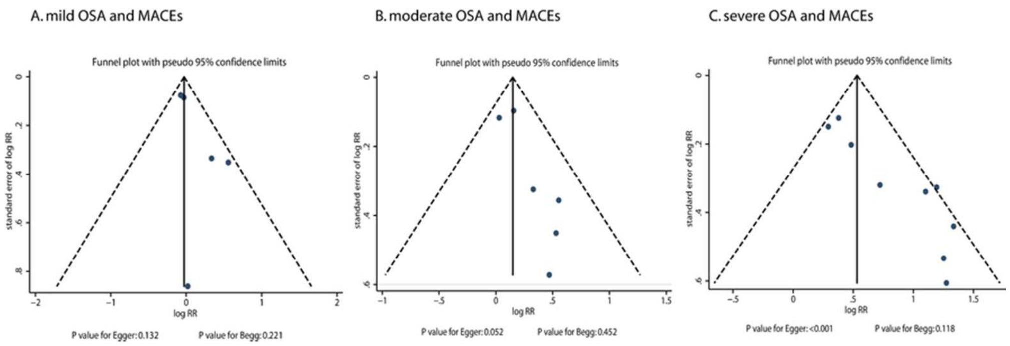

75x25mm (300 x 300 DPI)

peer review only

Title:

	**Search strategy**
**#1**	"Sleep Apnea, Obstructive" [Mesh] OR "OSA" [All fields] OR "OHS" [All fields]
**#2**	Apneas, Obstructive Sleep OR Obstructive Sleep Apneas OR Sleep Apneas, Obstructive OR Obstructive Sleep Apnea Syndrome OR Obstructive Sleep Apnea OR OSAHS OR Syndrome, Sleep Apnea, Obstructive OR Sleep Apnea Syndrome, Obstructive OR Apnea, Obstructive Sleep OR Sleep Apnea Hypopnea Syndrome OR Syndrome, Obstructive Sleep Apnea OR Upper Airway Resistance Sleep Apnea Syndrome OR Syndrome, Upper Airway Resistance, Sleep Apnea OR Hypoventilation Syndrome, Obesity OR Syndrome, Obesity Hypoventilation OR Pickwickian Syndrome OR Syndrome, Pickwickian OR Obesity-Hypoventilation Syndrome
**#3**	"Sleep Apnea Syndromes" [Mesh] OR "SAS" [All fields]
**#4**	Apnea Syndrome, Sleep OR Apnea Syndromes, Sleep OR Sleep Apnea Syndrome OR Apnea, Sleep OR Apneas, Sleep OR Sleep Apnea OR Sleep Apneas OR Sleep Hypopnea OR Hypopnea, Sleep OR Hypopneas, Sleep OR Sleep Hypopneas OR Sleep-Disordered Breathing OR Breathing, Sleep-Disordered OR Sleep Disordered Breathing OR Sleep Apnea, Mixed Central and Obstructive OR Mixed Central and Obstructive Sleep Apnea OR Sleep Apnea, Mixed OR Mixed Sleep Apnea OR Mixed Sleep Apneas OR Sleep Apneas, Mixed OR Hypersomnia with Periodic Respiration
**#5**	"Sleep Apnea, Central" [Mesh] OR "CSA"[All fields]
**#6**	Apneas, Central Sleep OR Central Sleep Apneas OR Sleep Apneas, Central OR Apnea, Central OR Apneas, Central OR Central Apnea OR Central Apneas OR Apnea, Central Sleep OR Apnea, Sleep, Central OR Sleep Apnea, Lethal Central OR Central Sleep Apnea OR Central Sleep Apnea Syndrome OR Central Sleep Disordered Breathing OR Hypoventilation, Central Alveolar OR Alveolar Hypoventilation, Central OR Alveolar Hypoventilations, Central OR Central Alveolar Hypoventilation OR Hypoventilations, Central Alveolar OR Ondine Syndrome OR Sleep-Disordered Breathing, Central OR Breathing, Central Sleep-Disordered OR Breathings, Central Sleep-Disordered OR Central Sleep-Disordered Breathing OR Central Sleep-Disordered Breathings OR Sleep Disordered Breathing, Central OR Sleep-Disordered Breathings, Central OR Central Alveolar Hypoventilation Syndrome OR Central Sleep Apnea, Secondary OR Secondary Central Sleep Apnea OR Sleep Apnea, Newborn, Primary OR Primary Sleep Apneas of Newborn OR Newborn Primary Sleep Apneas OR Central Sleep Apnea, Primary OR Primary Central Sleep Apnea
**#7**	"Continuous Positive Airway Pressure" [Mesh] OR "CPAP" [All fields] OR "Continuous Positive Airway Pressure/therapy" [Mesh]
**#8**	CPAP Ventilation OR Ventilation, CPAP OR Biphasic Continuous Positive Airway Pressure OR Bilevel Continuous Positive Airway Pressure OR Nasal Continuous Positive Airway Pressure OR nCPAP Ventilation OR Ventilation, nCPAP OR Airway Pressure Release Ventilation OR APRV Ventilation Mode OR APRV Ventilation Modes OR Ventilation Mode, APRV OR Ventilation Modes, APRV

For peer review only

**#9**	#1 OR #2 OR #3 OR #4 OR #5 OR #6 OR #7 OR #8
#10	"Cardiovascular System" [Mesh]
#11	"Death" [Mesh] OR Determination of Death OR Near-Death Experience OR Cardiac Death OR Death, Cardiac OR "Mortality" [Mesh] OR "mortality" [All fields] OR "Disease" [Mesh] OR "disease*"
**#12**	#10 AND #11
**#13**	"Cardiovascular Diseases" [Mesh] OR "CVD" OR Cardiovascular Disease OR Disease, Cardiovascular OR Diseases, Cardiovascular
**#14**	"Myocardial Infarction" [Mesh] OR "MI" OR Infarction, Myocardial OR Infarctions, Myocardial OR Myocardial Infarctions OR Cardiovascular Stroke OR Cardiovascular Strokes OR Stroke, Cardiovascular OR Strokes, Cardiovascular OR Heart Attack OR Heart Attacks OR Myocardial Infarct OR Infarct, Myocardial OR Infarcts, Myocardial OR Myocardial Infarcts
**#15**	"Angina Pectoris" [Mesh] OR "Angina, Stable" [Mesh] OR "Microvascular Angina" [Mesh] OR "Angina, Unstable" [Mesh] OR Stenocardia OR Stenocardias OR Angor Pectoris OR "angina" [All fields] OR "Coronary Artery Disease" [Mesh] OR "CAD" OR "ischemic heart disease" [All fields] OR "Heart Failure" [Mesh] OR "Heart Failure, Diastolic" [Mesh] OR "Heart Failure, Systolic" [Mesh]
**#16**	"Cerebrovascular Disorders" [Mesh] OR "cerebrovascular" [All fields] OR "stroke*"
**#17**	"Death" [Mesh] OR Determination of Death OR Near-Death Experience OR Cardiac Death OR Death, Cardiac OR "Mortality" [Mesh] OR "mortality" [All fields] OR "mortality*"
**#18**	#12 OR #13 OR #14 OR #15 OR #16 OR #17
**#19**	"Prospective Studies" [Mesh] OR "Cohort Studies" [Mesh] OR "Follow-Up Studies" [Mesh] OR "prospective study" OR "cohort study" OR "follow-up study"
**#20**	#9 AND #18 AND #19

For peer review only

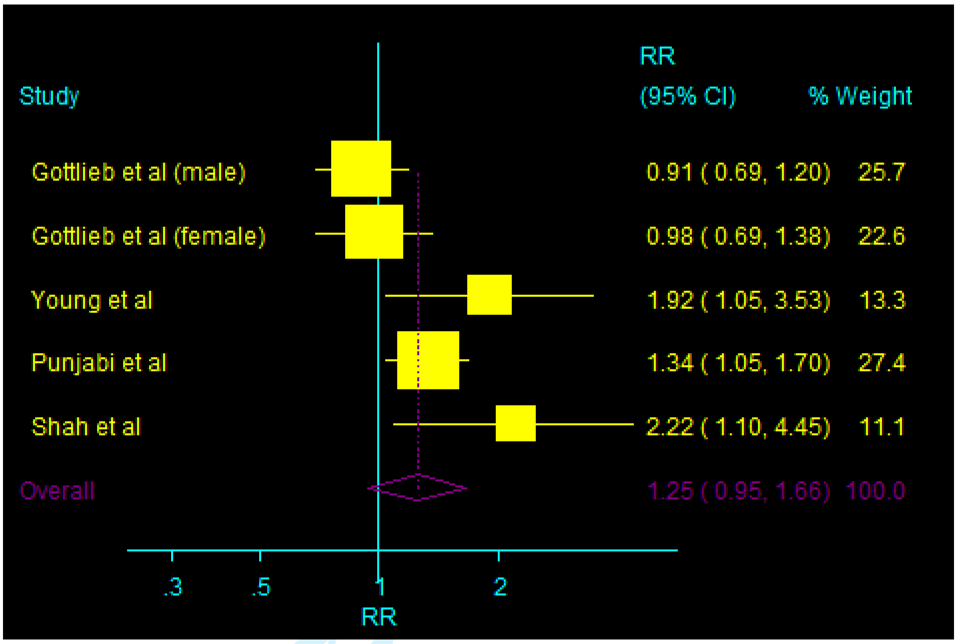

Figure S1. Association between mild OSA and CHD.

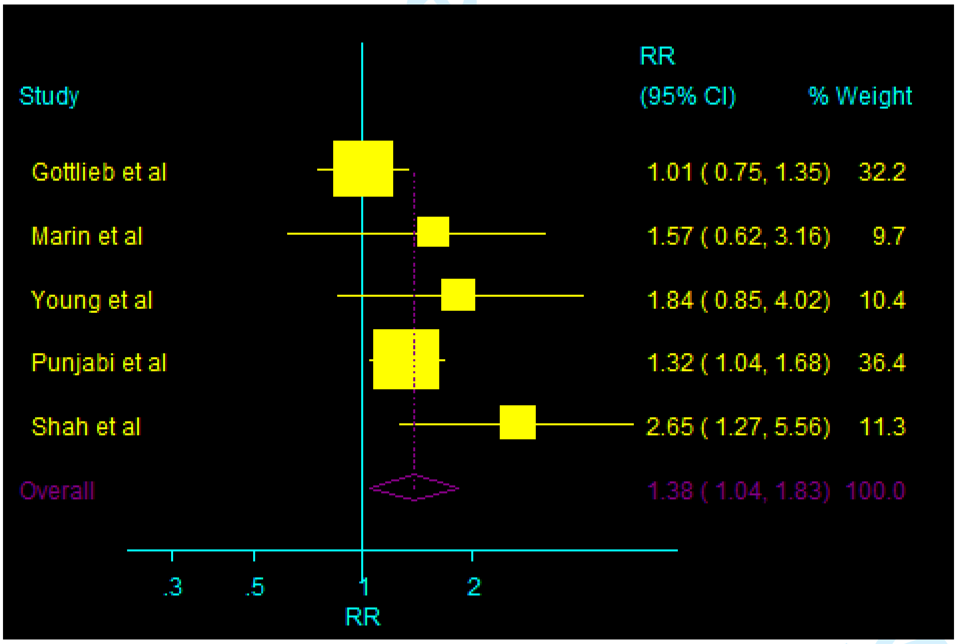

Figure S2. Association between moderate OSA and CHD.

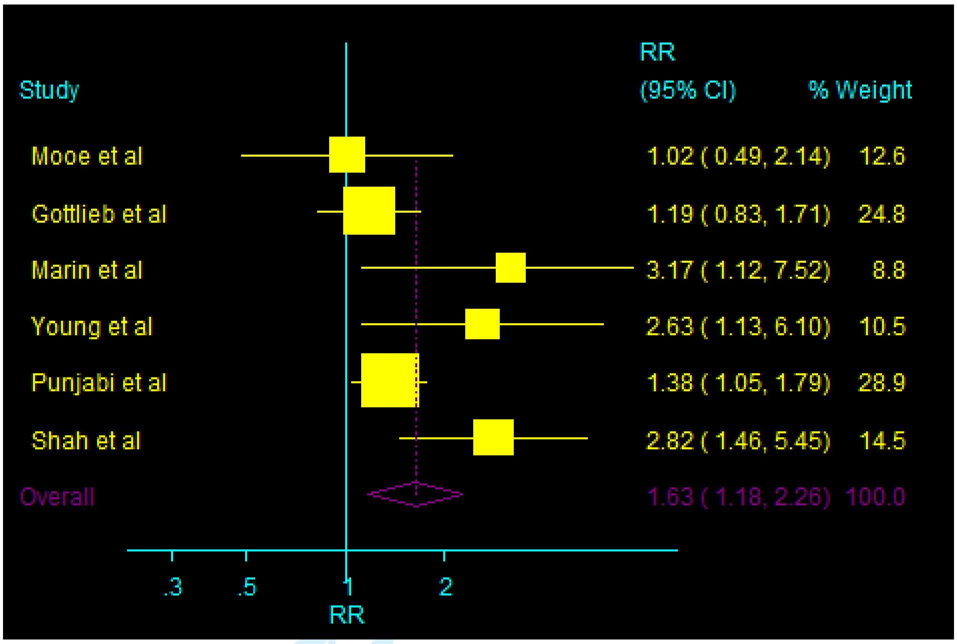

Figure S3. Association between severe OSA and CHD.

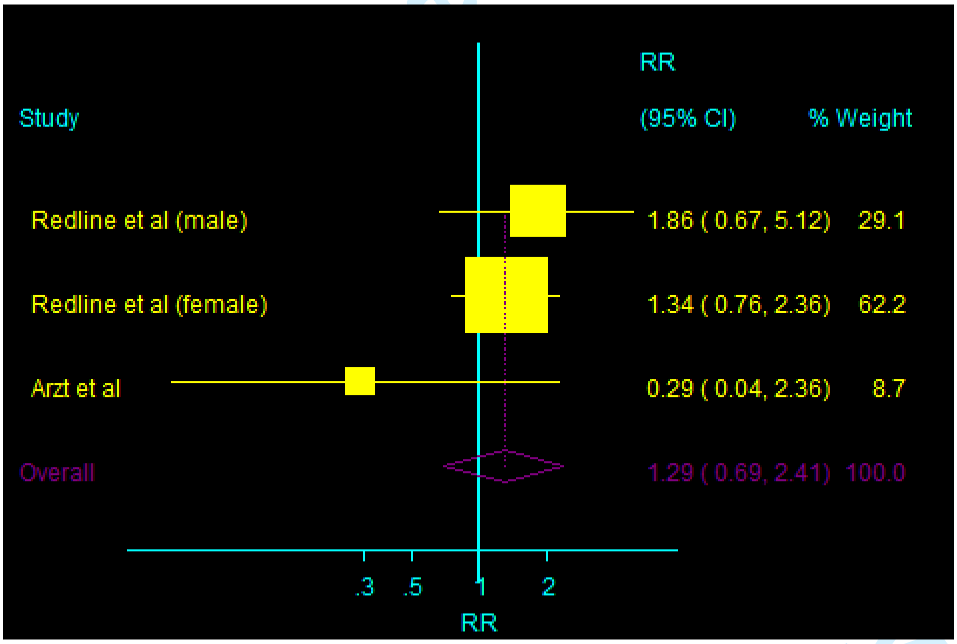

Figure S4. Association between mild OSA and stroke.

For peer review only - http://bmjopen.bmj.com/site/about/guidelines.xhtml

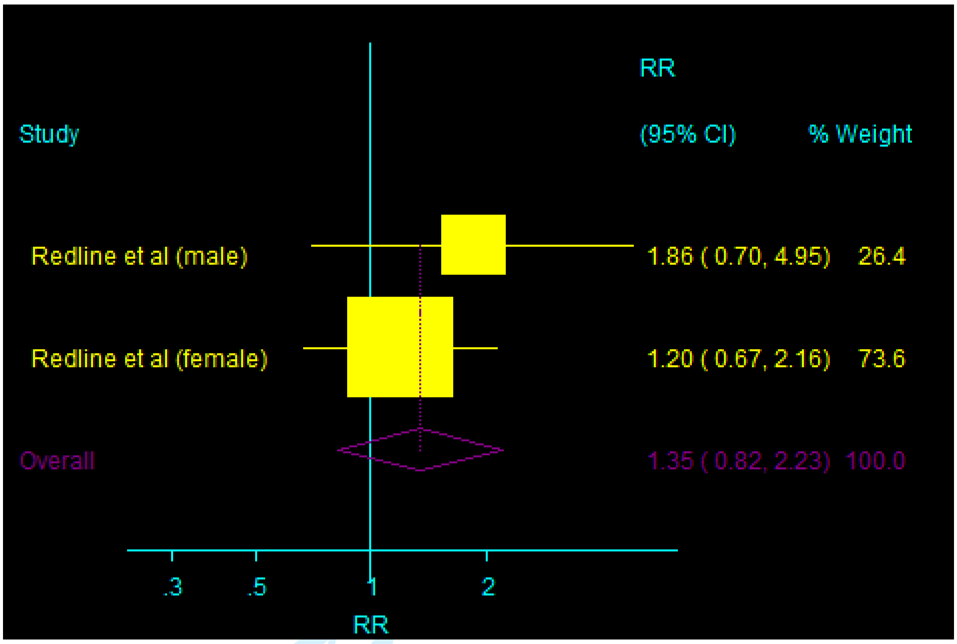

Figure S5. Association between moderate OSA and stroke.

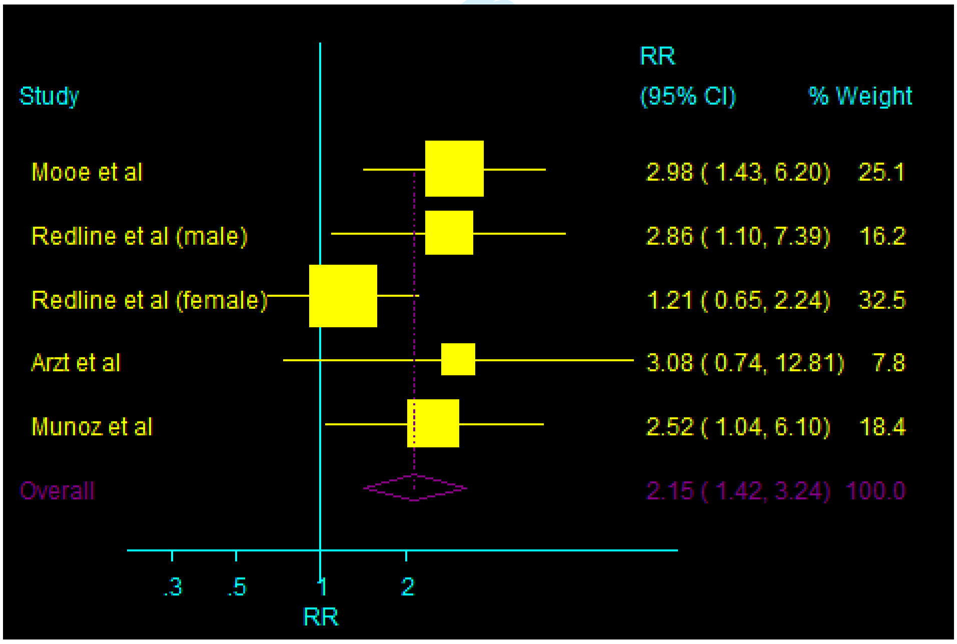

Figure S6. Association between severe OSA and stroke

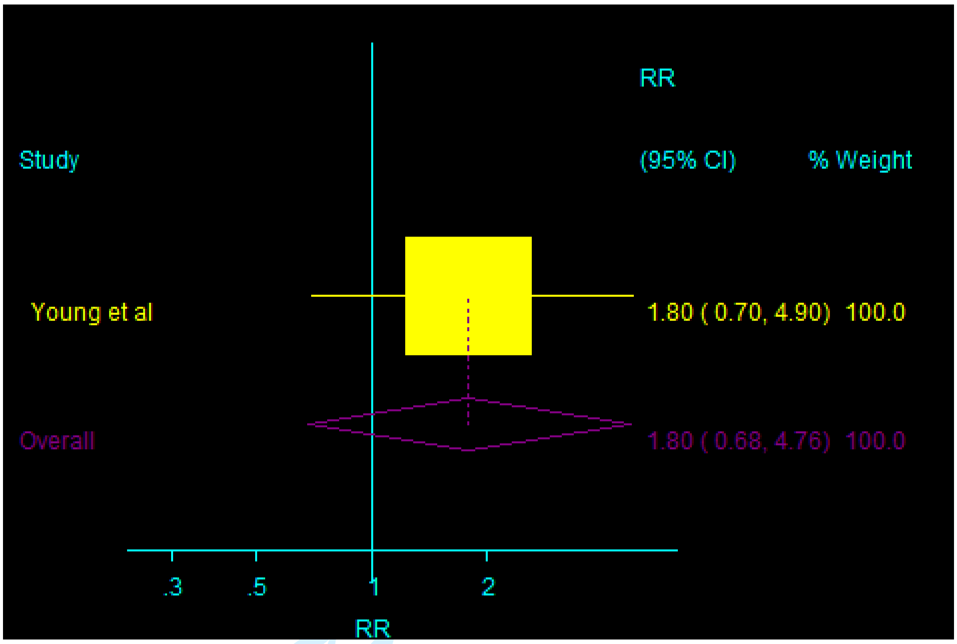

Figure S7. Association between mild OSA and cardiac death.

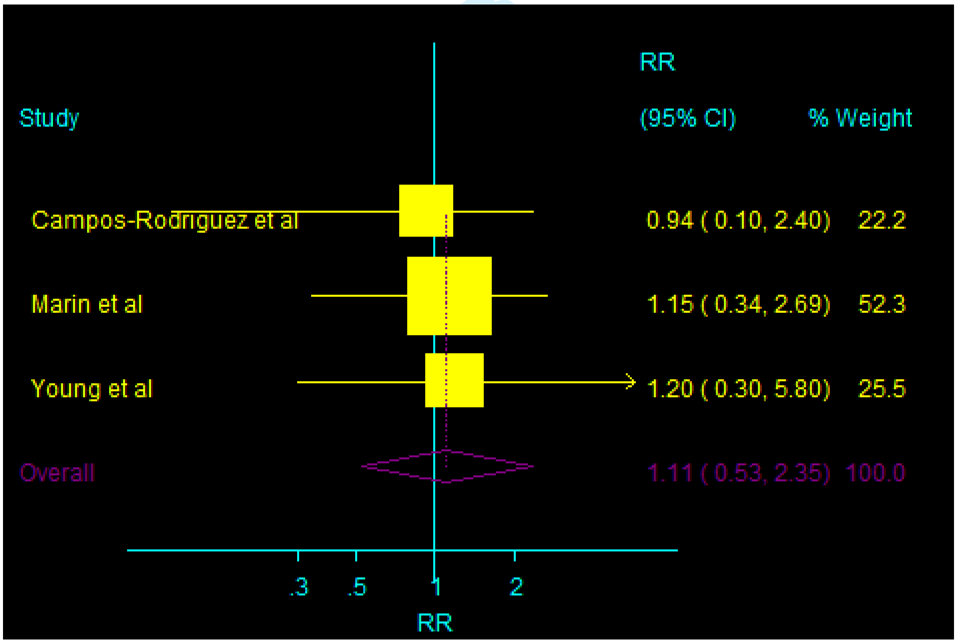

Figure S8. Association between moderate OSA and cardiac death.

For peer review only - http://bmjopen.bmj.com/site/about/guidelines.xhtml

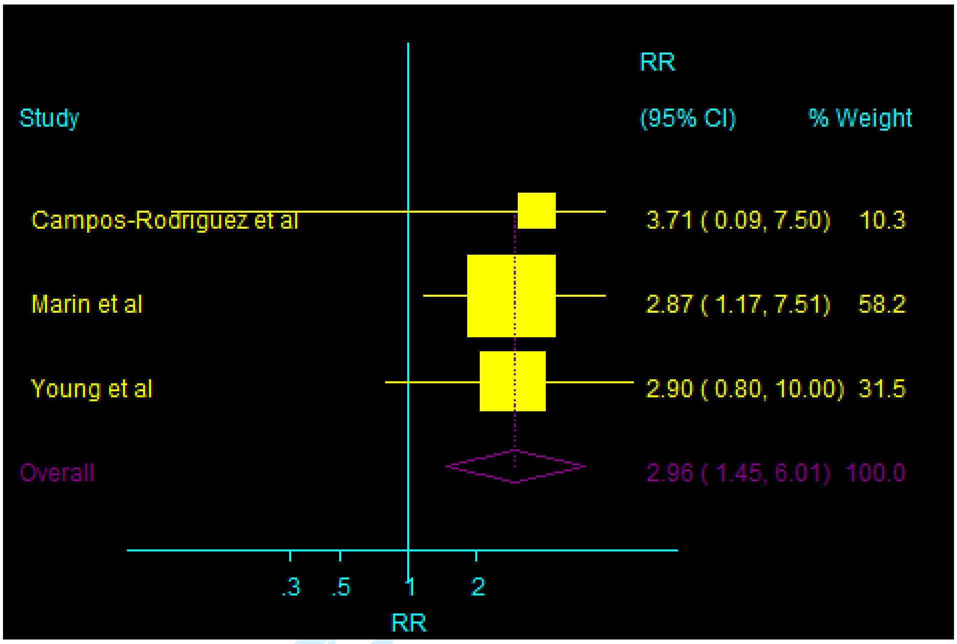

Figure S9. Association between severe OSA and cardiac death.

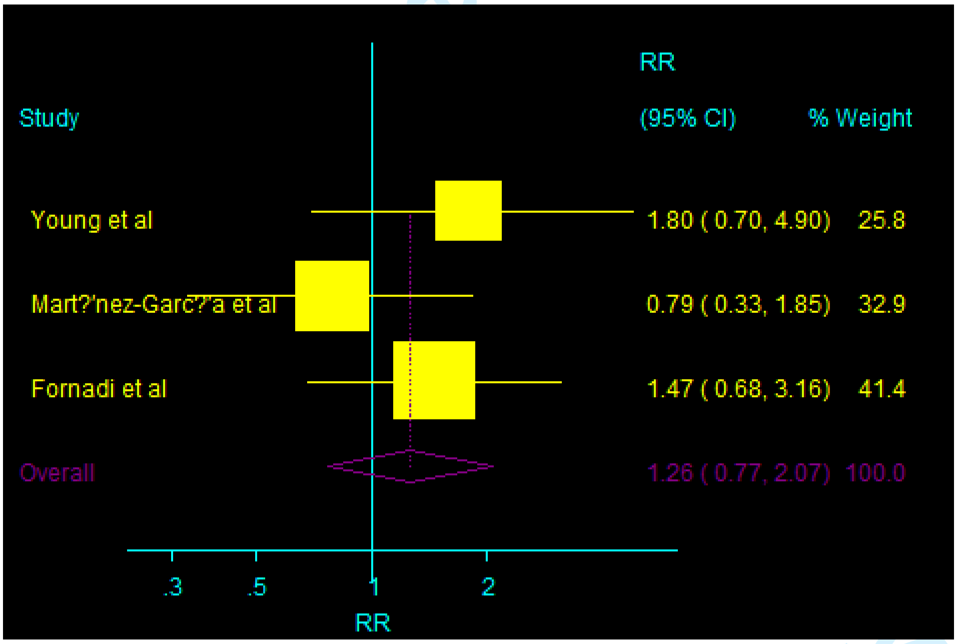

Figure S10. Association between mild OSA and all-cause death.

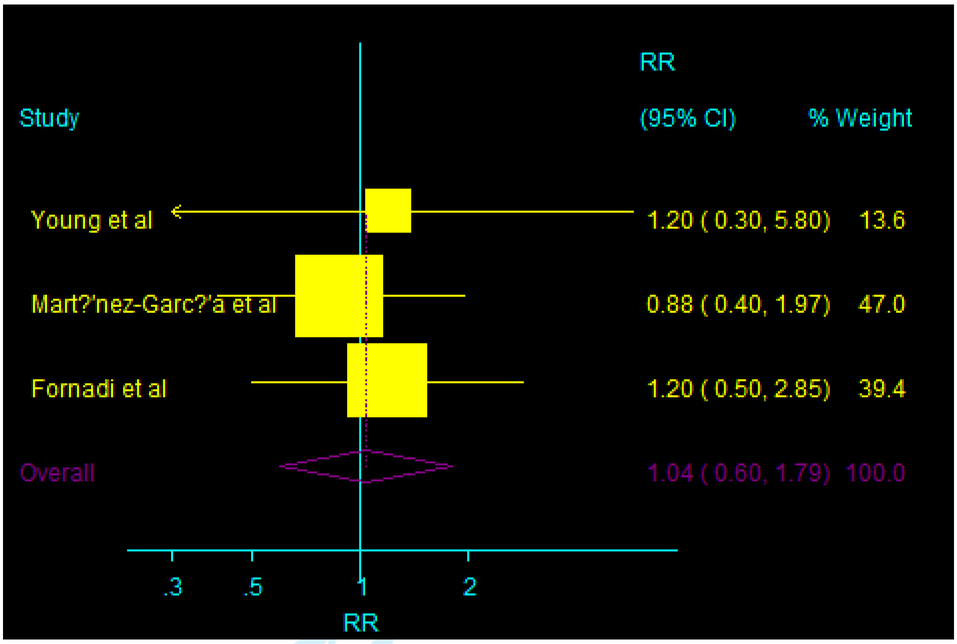

Figure S11. Association between moderate OSA and all-cause death.

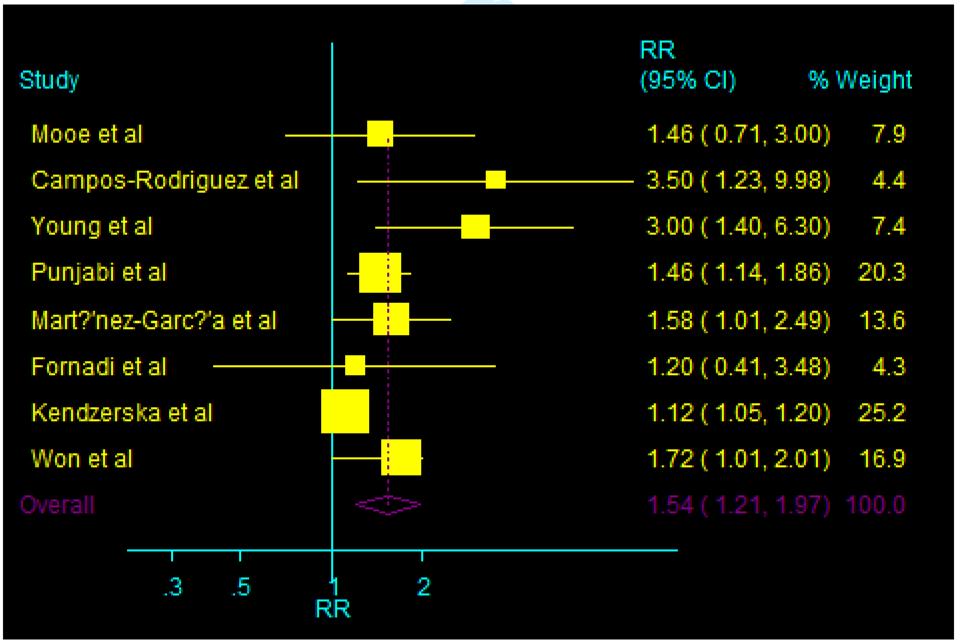

Figure S12. Association between severe OSA and all-cause death.

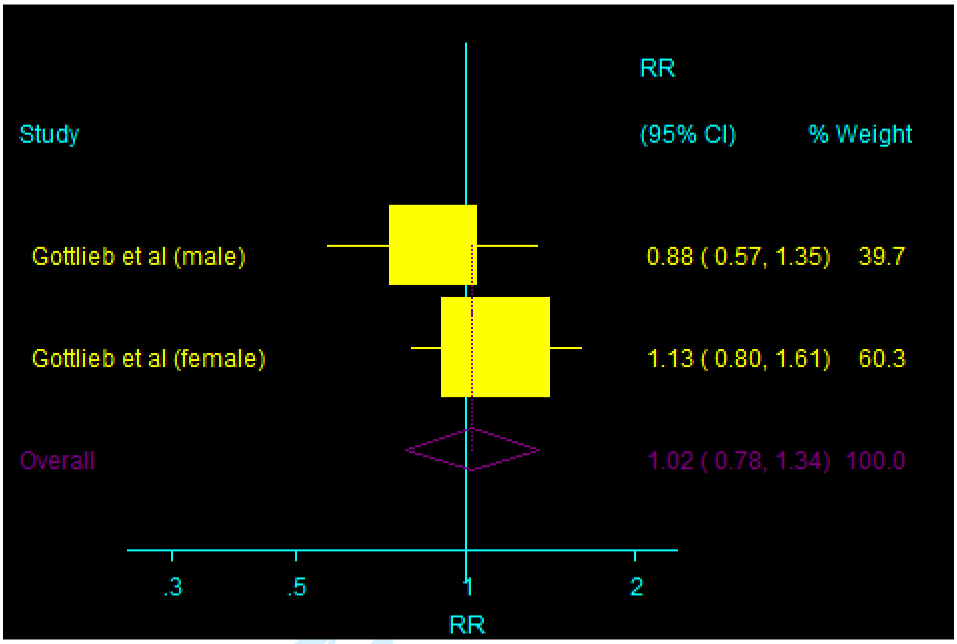

Figure S13. Association between mild OSA and heart failure.

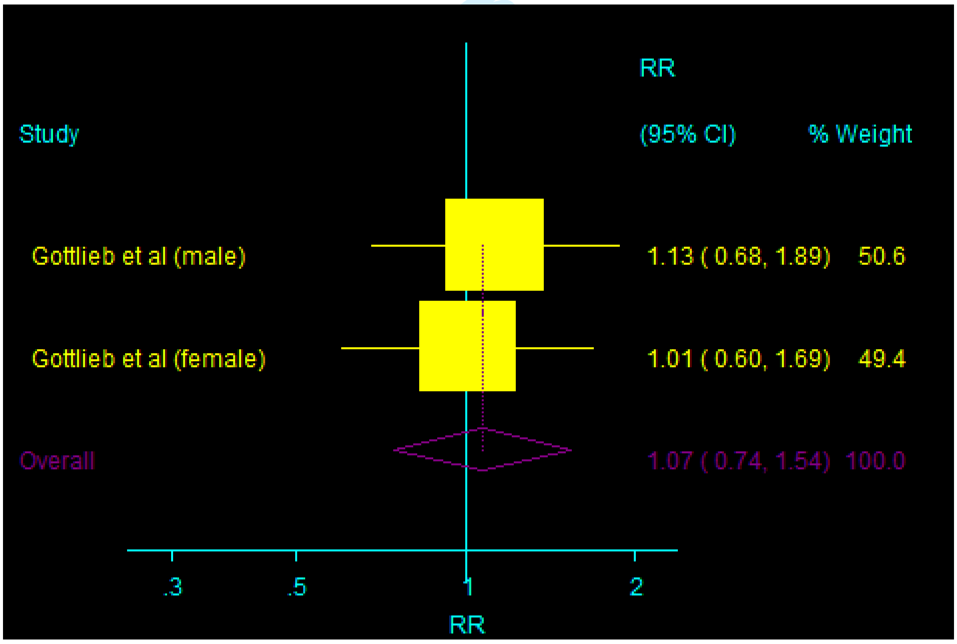

Figure S14. Association between moderate OSA and heart failure.

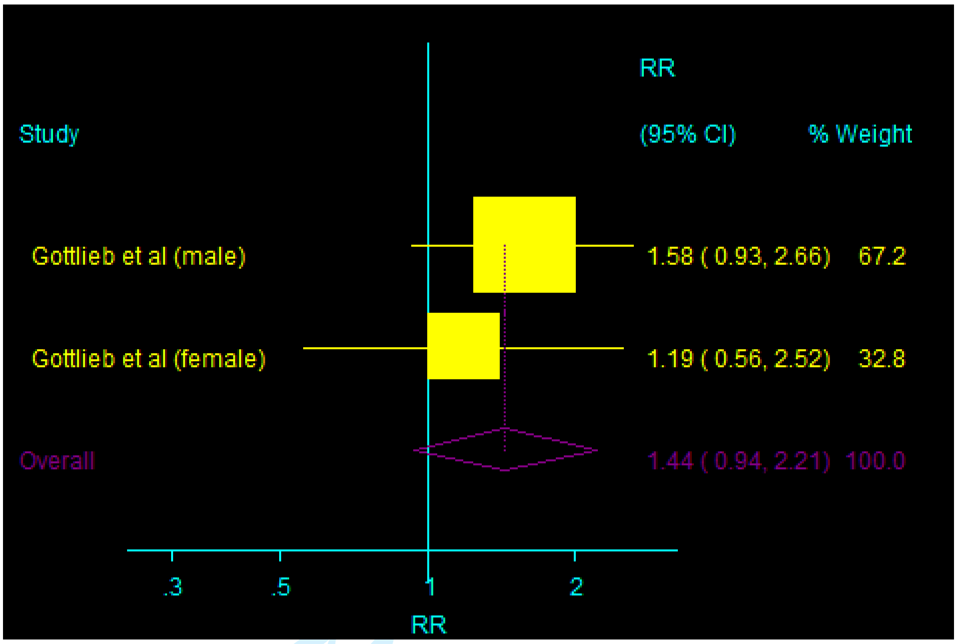

Figure S15. Association between severe OSA and heart failure.

MOOSE Statement: Reporting Checklist for Authors, Editors, and Reviewers of Meta-analyses of Observational Studies

Reporting Criteria	Reported (Yes/No)	Reported on Page
**Reporting of background should include**		
Problem definition	Yes	3
Hypothesis statement	Yes	3
Description of study outcomes	Yes	3
Type of exposure or intervention used	Yes	3 – 4
Type of study designs used	Yes	4
Study population	Yes	4
**Reporting of search strategy should include**		
Qualifications of searchers (e.g., librarians and investigators)	Yes	4 – 5
Search strategy, including time period used in the synthesis and key words	Yes	5
Effort to include all available studies, including contact with authors	Yes	5
Databases and registries searched	Yes	4–5
Search software used, name and version, including special features used (e.g., explosion)	Yes	4–5
Use of hand searching (e.g., reference lists of obtained articles)	Yes	5
List of citations located and those excluded, including justification	Yes	8
Method of addressing articles published in languages other than English	Yes	4
Method of handling abstracts and unpublished studies	Yes	4–5
Description of any contact with authors	No	NA
**Reporting of methods should include**		
Description of relevance or appropriateness of studies assembled for assessing the hypothesis to be tested	No	5
Rationale for the selection and coding of data (e.g., sound clinical principles or convenience)	Yes	5–6
Documentation of how data were classified and coded (e.g., multiple raters, blinding and inter-rater reliability)	Yes	5–6
Assessment of confounding (e.g., comparability of cases and controls in studies where appropriate)	Yes	6
Assessment of study quality, including blinding of quality assessors, and stratification or regression on possible predictors of study results	Yes	6

For peer review only

Assessment of heterogeneity	Yes	7
Description of statistical methods (e.g., complete description of fixed- or random-effects models, justification of whether the chosen models account for predictors of study results, dose–response models, or cumulative meta-analysis) in sufficient detail to be replicated	Yes	6–7
Provision of appropriate tables and graphics	Yes	6–7
**Reporting of results should include**		
Graphic summarizing individual study estimates and overall estimate	Yes	8
Table giving descriptive information for each study included	Yes	8–14
Results of sensitivity testing (e.g., subgroup analysis)	Yes	15–20
Indication of statistical uncertainty of findings	Yes	20
**Reporting of discussion should include**		
Quantitative assessment of bias (e.g., publication bias)	Yes	20
Justification for exclusion (e.g., exclusion of non-English language citations)	No	21
Assessment of quality of included studies	Yes	Table 1
Strengths and weaknesses	Yes	23
**Reporting of conclusions should include**		
Consideration of alternative explanations for observed results	Yes	20–23
Generalization of the conclusions (e.g., appropriate for the data presented and within the domain of the literature review)	Yes	23
Guidelines for future research	Yes	23
Disclosure of funding source	Yes	24

NA, Not applicable.

For peer review only
